# Comparative efficacy of five approved Janus kinase inhibitors as monotherapy and combination therapy in patients with moderate-to-severe active rheumatoid arthritis: a systematic review and network meta-analysis of randomized controlled trials

**DOI:** 10.3389/fphar.2024.1387585

**Published:** 2024-04-24

**Authors:** Wenting Cai, Rui Tong, Yue Sun, Yao Yao, Jinping Zhang

**Affiliations:** ^1^ Department of Pharmacy, Nanjing Drum Tower Hospital, School of Basic Medicine and Clinical Pharmacy, China Pharmaceutical University, Nanjing, China; ^2^ Department of Pharmacy, Nanjing Drum Tower Hospital, Affiliated Hospital of Medical School, Nanjing University, Nanjing, China

**Keywords:** Janus kinase inhibitors, conventional synthetic disease-modifying anti-rheumatic drugs, network meta-analysis, rheumatoid arthritis, efficacy, safety

## Abstract

**Background:**

The European League of Rheumatology(EULAR)guidelines recommend Janus kinase (JAK) inhibitors for patients with moderate to severe rheumatoid arthritis (RA) who are insensitive or under-responsive to conventional synthetic disease-modifying anti-rheumatic drugs (csDMARDs). But there was no recommendation for which one was preferred in five currently approved JAK inhibitors. The objective of this network meta-analysis study was to evaluate the efficacy of five JAK inhibitors as monotherapy and combination therapy in patients with moderate-to-severe active rheumatoid arthritis.

**Methods:**

The randomized controlled trials (RCTs) of tofacitinib, baricitinib, upadacitinib, filgotinib and peficitinib as monotherapy or combined with csDMARD in the treatment of active RA were searched in database of PubMed, Embase, Web of Science and Cochrane Library, up to December 2023. The control group included placebo or csDMARD. Outcome indicators included American College of Rheumatology 20% response (ACR20), ACR50, ACR70 and the percentage of patients achieving 28-joint disease activity score using C-reactive protein (DAS28(CRP))<2.6 at 12 weeks and 24 weeks. The statistical analysis was performed by Stata14 and RevMan5.4. Data processing, network evidence plots, surface under the cumulative ranking curve (SUCRA) ranking, league plots and funnel plots were generated. Risk ratio (RR) and 95% confidence interval (95%CI) as effect sizes to analyze the statistics.

**Results:**

This study included thirty-six RCTs with 16,713 patients. All JAK inhibitors were more effective than placebo in ACR20 (RRs ranging between 1.74 and 3.08), ACR50 (RRs ranging between 2.02 and 7.47), ACR70 (RRs ranging between 2.68 and 18.13), DAS28(CRP) < 2.6 (RRs ranging between 2.70 and 7.09) at 12 weeks. Upadacitinib 30 mg and upadacitinib 15 mg showed relatively good efficacy according to their relative SUCRA ranking. All JAK inhibitors were more effective than csDMARD or placebo in ACR20 (RRs ranging between 1.16 and 1.86), ACR50 (RRs ranging between 1.69 and 2.84), ACR70 (RRs ranging between 1.50 and 4.47), DAS28(CRP) < 2.6 (RRs ranging between 2.28 and 7.56) at 24 weeks. Upadacitinib 15 mg + csDMARD and baricitinib 4 mg + csDMARD showed relatively good efficacy according to their relative SUCRA ranking. The safety analysis results such as serious infection, malignancy, major adverse cardiovascular event (MACE), and venous thromboembolic events (VTE) showed no statistical difference.

**Conclusion:**

This NMA study indicated that all JAK inhibitors performed better than placebo. Based on the results of this study, upadacitinib 30 mg, upadacitinib 15 mg, upadacitinib 15 mg + csDMARD and baricitinib 4 mg + csDMARD were recommended treatment options with relatively good efficacy and safety. However, attention should be paid to monitoring the occurrence of adverse events in high-risk RA patients with medication. Combination therapy with csDMARD might be more suitable for the maintenance of long-term efficacy. However, in clinical practice, it is still necessary to select the appropriate therapeutic regimen based on the actual clinical situation.

## 1 Introduction

RA is defined as a systemic autoimmune disease associated with chronic inflammatory processes that can damage joints and extra-articular organs, including the heart, kidneys, lungs, digestive system, eyes, skin, and nervous system (Conforti et al., 2021). Its pathological features include inflammatory cell infiltration, synovial hyperplasia, and progressive damage to articular cartilage and subchondral bone (Mcinnes and Schett, 2011). Epidemiological survey shows that the prevalence of RA in China is 0.39%–0.45%, and it is more common in women, usually between the ages of 30–60 years old, and its epidemiological burden and economic burden are heavy (Cross et al., 2014). Systemic manifestations caused by chronic inflammatory state in RA patients seriously affect patients’ quality of life and lead to increased mortality (Lassere et al., 2013). The most effective treatment requires early diagnosis as well as optimal medication and regular evaluation of the efficacy and safety of the treatment.

csDMARDs are the first-line drugs for RA treatment, of which methotrexate (MTX) is the cornerstone drug and monotherapy is recommended as the first choice (Smolen et al., 2023). However, previous studies had shown that 50% of RA patients had poor treatment effect on methotrexate or inadequate response to re-medication after relapse, resulting in drug resistance (Lima et al., 2015), resulting in no significant relief of symptoms and still high disease activity. EULAR had indicated that interleukin-6 (IL-6) receptor inhibitors and JAK inhibitors may have advantages over other biological disease-modifying anti-rheumatic drugs (bDMARDs) in patients who were not suitable for csDMARDs (Gremese et al., 2019). Therefore, JAK inhibitors is used as monotherapy or combination therapy, which would provide a new strategy for clinical treatment (Guo et al., 2018).

There are four main subtypes of JAK, including JAK1, JAK2, JAK3, and tyrosine kinase 2 (Tyk2) (Kubo et al., 2023). By inhibiting JAK, multiple cytokine signals and inflammatory pathways can be simultaneously suppressed, reducing the attack of immune cells on the joints, which can slow the progression of the disease. The application of JAK inhibitors in the treatment of RA is developing rapidly. From the emergence of the first JAK inhibitors to their subsequent development, they have provided a new targeted oral therapy that has greatly improved compliance of patient (Atzeni et al., 2018). Moreover, a number of clinical studies have shown the comparable efficacy and safety of JAK inhibitors and tumor necrosis factor inhibitors (TNFi), making JAK inhibitors to be a particularly attractive new treatment option for RA patients (Gremese et al., 2019).

Currently approved JAK inhibitors for the treatment of RA are tofacitinib, baricitinib, upadacitinib, filgotinib and peficitinib. Tofacitinib is a selective inhibitor of JAK1 and JAK3 and is recommended to be taken 5 mg twice a day orally. It was the first small molecule JAK inhibitors approved by the U.S. Food and Drug Administration (FDA) for the treatment of RA in 2012. Baricitinib is a selective inhibitor of JAK1 and JAK2 and is recommended to be taken 2 mg once a day orally, with an increased dose to 4 mg daily for patients with moderate-to-severe disease activity or poor response to RA. Upadacitinib is a selective inhibitor of JAK1 and is recommended as an oral dose of 15 mg or 30 mg once a day. When the response to MTX is insufficient, the adjustment to upadacitinib monotherapy can quickly relieve the disease, and the incidence of adverse reactions is also low (Van Vollenhoven et al., 2020). Filgotinib selectively targets JAK1 to maximize efficacy and safety. The recommended dose of filgotinib in adults is 200 mg once daily. For RA patients with multiple adverse prognostic factors, filgotinib is also effective, with a safety period of up to 4 years (Kavanaugh et al., 2021). Peficitinib is a novel JAK inhibitor that selectively inhibits JAK3 and is approved for the treatment of RA in Japan and South Korea. In Japan, the recommended dose of peficitinib is 150 mg once daily, which could be adjusted to 100 mg once daily. Pain and activity scores would be rapidly reduced by peficitinib after 12 weeks of administration with safety for 2 years (Takeuchi et al., 2021; Tanaka et al., 2022). Although there are different types of JAK inhibitors, there is no optimal choice in current RA treatment guidelines. As a result, it is necessary to study the comparative efficacy of JAK inhibitors in RA patients.

However, based on the latest trial data of ORAL-Surveillance, tofacitinib has a higher incidence of MACEs and malignant tumors when used in the treatment of RA patients who have cardiovascular risk factors compared with TNFi (Agency, 2021). This experimental substudy also showed that compared with TNFi, tofacitinib also significantly increased the rate of infection outside herpes zoster (Balanescu et al., 2022). Although JAK inhibitors has comparable efficacy over TNFi, safety concerns have been identified in RA patient populations. The U.S. FDA has also added warnings about “the increased risk of serious heart-related events, cancer, VTE, and death associated with the treatment of JAK inhibitors to certain chronic inflammatory” (Ufad, 2021). Therefore, the safety of JAK inhibitors also needs to be further verified.

There are no available direct comparative studies among JAK inhibitors. NMA has evolved from the conventional pairwise meta-analysis (Lin et al., 2020). Based on the findings of the present study, NMA has the capability to concurrently compare various JAK inhibitors treatment regimens through both direct and indirect comparisons. By conducting a thorough analysis of the results from these comparisons, the study was able to rank the effects of different JAK inhibitors treatment regimens. Therefore, the NMA method was employed in this study to compare the efficacy and safety of various JAK inhibitors treatment regimens in the treatment of RA, offering a foundation for selecting the optimal treatment regimen for clinical treatment of RA.

## 2 Methods

### 2.1 Search strategy

We conducted a comprehensive search on databases PubMed, Embase, Web of Science and Cochrane Library, with a search deadline of December 2023 for each database since its establishment. The following descriptors were used: “Tofacitinib OR Xeljanz OR CP-690,550” “Baricitinib OR INCB-28050 OR LY-3009104” “Upadacitinib OR ABT-494 OR Rinvoq” “Filgotinib” “Peficitinib OR ASP015K” “Rheumatoid arthritis” “Randomized controlled trial”. Expand the search of the included references in the database.

### 2.2 Inclusion criteria

(1) Participants: patients were adults (≥18 years) diagnosed with moderate-to-severe active RA (Aletaha et al., 2010).
(2) Interventions: patients in the experimental group received tofacitinib (5 mg,bid), baricitinib (2 mg or 4 mg, qd), upadacitinib (15 mg or 30 mg, qd), filgotinib (200 mg, qd) or peficitinib (100 mg or 150 mg, qd), whether monotherapy or in combination with (+)csDMARD. Patients in the control group received placebo or csDMARD.
(3) Outcome indicators: the efficacy indicators mainly included the response rate of ACR20, ACR50, ACR70 and the percentage of patients achieving DAS28(CRP) < 2.6 at 12 weeks (12–14 weeks) or 24 weeks (24–26 weeks). ACR20 is defined by American College of Rheumatology as a minimum of 20% improvement both in the number of swollen and tender joints and in three of the additional five measures: patient global assessment, physician global assessment, Health Assessment Questionnaire, visual analog pain scale, and erythrocyte sedimentation rate or CRP (Felson et al., 1993). The definition of ACR50 and ACR70 were similar, with a level of improvement of 50% and 70%, respectively. The safety indicators mainly include adverse events (AE), serious adverse events (SAE), AE leading to discontinuation, infection, serious infection, malignancy, MACE, and VTE.
(4) Research type: only randomized controlled trials (RCT).
(5) Language type: unlimited.

### 2.3 Exclusion criteria

(1) Researchs such as review, abstract, report, editorials and animal experiments.
(2) Researchs with incomplete data.
(3) Researchs published repeatedly.
(4) Not RCT.

### 2.4 Literature selection and data collection

We managed all of the literature using NoteExpress software. Two people independently carried out an initial screening of all the literature after defining and harmonizing the screening criteria. A third person would study the entire text of literature containing divergent viewpoints and determine whether or not to include it. Data extraction was done with Excel 2019. One person entered the data and the other checked it. The extracted content includes the name of the first author, the year of publication, study region, the number of patients, intervention measure, dose (mg), concomitant medication, treatment duration (wk), participants, trial identifier.

### 2.5 Quality assessment

We evaluated the bias risk of the included literature according to the RCT bias assessment tool in Cochrane’s manual. The bias risk assessment comprehensively considered seven aspects, including random sequence generation, allocation concealment, blinding of participants and personnel, blinding of outcome assessment, incomplete outcome data, selective reporting, and other bias. Two individuals separately assessed the risk of bias in the quality of the literature, and for literature with opposing viewpoints, a third person evaluated it.

### 2.6 Statistical analysis

RevMan5.4 was used to assess the quality of the literature and determine the risk of bias map. Stata14 software was used to conduct a network meta-analysis. Data processing, network evidence plots, surface under the cumulative ranking curve (SUCRA) ranking, league plots and funnel plots were generated. The outcome indicators of this study are binary variable data. Risk ratio (RR) and 95% confidence interval (95%CI) were used as effect sizes to analyze the statistics. Global inconsistency tests were performed. *p* > 0.05 indicates no significant inconsistency. SUCRA was used to visually analyze the effects of interventions on each outcome measure. SUCRA values range from 0 to 100, and the closer the value is to 100, the more effective the intervention is considered to be (Rucker and Schwarzer, 2015). A comparison-corrected funnel plot was drawn to assess the presence of small-sample effects or publication bias. Conduct subgroup analysis based on the different duration (12 weeks or 24 weeks).

## 3 Results

### 3.1 Literature screening results

**FIGURE 1:**
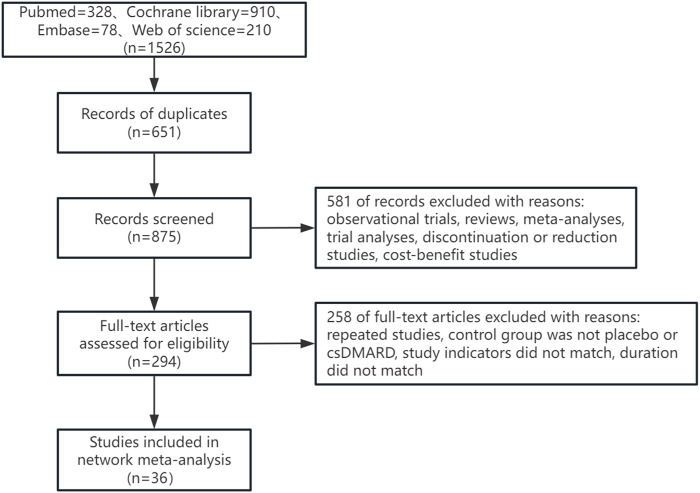
Flow diagram of the selection process of literature.

A total of 1,526 articles were retrieved in this study, with 651 duplicate articles excluded. 875 articles were included in the initial screening. 581 articles were excluded by reading the title and abstract of the articles, and 258 articles were excluded by reading the full text of the articles. Finally, 36 articles were included. The literature retrieval process was shown in Figure 1.

### 3.2 Study and participant characteristics

As shown in Table 1, the 36 RCT studies included 14,841 patients worldwide. All patients were moderate-to-severe active RA, regardless of DMARD-naive or DMARD-inadequate response, such as csDMARD-inadequate response or bDMARD-inadequate response. Tofacitinib was researched in 10 studies, while baricitinib, upadacitinib, filgotinib and peficitinib was researched in 8, 7, six and five studies respectively. There was a total of 13 studies (Fleischmann et al., 2012a; Fleischmann et al., 2012b; Lee et al., 2014; Tanaka et al., 2015; Takeuchi et al., 2016; Fleischmann et al., 2017; Genovese et al., 2017; Smolen et al., 2019; Tanaka et al., 2019; Harigai et al., 2020; Van Vollenhoven et al., 2020; Westhovens et al., 2021; Atsumi et al., 2023) as monotherapy. Three studies (Fleischmann et al., 2017; Westhovens et al., 2021; Atsumi et al., 2023) had interventions that included both monotherapy and combination therapy. However, monotherapy studies of baricitinib 2 mg were lacking at the 12-week study time point. At the 24-week study time point, there was a lack of studies of monotherapy studies of baricitinib 2 mg, peficitinib 100 mg and peficitinib 150 mg. There was also a lack of studies of the combination therapy of upadacitinib 30 mg + csDMARD, peficitinib100 mg + csDMARD and peficitinib 150 mg + csDMARD. As shown in Supplementary Material, Patients’ age, course of disease, sex ratio and DAS28(CRP) score at baseline were balanced and comparable.

**TABLE 1:** Study characteristics of included RCTs.

Author, year	Study region	N	Experimental group	Dose (mg)	Concomitant medication	Control group	Treatment duration (wk)	Participants
Fleischmann et al. (2012a) (NCT00550446)	Worldwide	361	Tofacitinib	5 mg bid	Monotherapy	Placebo	12	DMARD-IR
Tanaka et al. (2015) (NCT00687193)	Japan	158	Tofacitinib	5 mg bid	Monotherapy	Placebo	12	DMARD-IR
^a^ Fleischmann et al. (2012b) (NCT00550446)	Worldwide	108	Tofacitinib	5 mg bid	Monotherapy	Placebo	12/24	DMARD-IR
Lee et al. (2014) (NCT01039688)	Worldwide	553	Tofacitinib	5 mg bid	Monotherapy	MTX	12/24	MTX-Naive
Van Vollenhoven et al. (2012) (NCT00853385)	Worldwide	302	Tofacitinib	5、10 mg bid	MTX	MTX	12/24	MTX-IR
Van Der Heijde et al. (2019) (NCT00847613)	Worldwide	463	Tofacitinib	5、10 mg bid	MTX	MTX	12/24	MTX-IR
Burmester et al. (2013) (NCT00960440)	Worldwide	263	Tofacitinib	5、10 mg bid	MTX	MTX	12	bDMARD-IR
Kremer et al. (2013) (NCT00856544)	Worldwide	468	Tofacitinib	5、10 mg bid	MTX	MTX	12/24	DMARD-IR
Tanaka et al. (2011)/	Japan	55	Tofacitinib	5、10 mg bid	MTX	MTX	12	MTX-IR
Kremer et al. (2012) (NCT00413660)	Worldwide	140	Tofacitinib	5、10 mg bid	MTX	MTX	12/24	MTX-IR
Harigai et al. (2020) (NCT00902486)	the United States, Czechia	62	Baricitinib	4 mg qd	Monotherapy	Placebo	12	bDMARD-IR
Fleischmann et al. (2017) (NCT01711359)	Worldwide	584	Baricitinib	4 mg qd	Monotherapy/MTX	MTX	12/24	MTX-Naive
Taylor et al. (2017) (NCT01710358)	Worldwide	975	Baricitinib	4 mg qd	MTX	MTX	12/24	MTX-IR
Keystone et al. (2015) (NCT01185353)	Worldwide	202	Baricitinib	2、4 mg qd	MTX	MTX	12/24	MTX-IR
Tanaka et al. (2016) (NCT01469013)	Japan	97	Baricitinib	2、4 mg qd	MTX	MTX	12	MTX-IR
Li et al. (2020) (NCT02265705)	China, Brazil,Argentina	290	Baricitinib	4 mg qd	MTX	MTX	12/24	MTX-IR
Genovese et al. (2016) (NCT01721044)	Worldwide	527	Baricitinib	2、4 mg qd	csDMARD	csDMARD	12/24	bDMARD-IR
Dougados et al. (2017) (NCT01721057)	Worldwide	684	Baricitinib	2、4 mg qd	csDMARD	csDMARD	12/24	csDMARD-IR
Smolen et al. (2019) (NCT02706951)	Worldwide	648	Upadacitinib	15、30 mg qd	Monotherapy	MTX	14	MTX-IR
Van Vollenhoven et al. (2020) (NCT02706873)	Worldwide	945	Upadacitinib	15、30 mg qd	Monotherapy	MTX	12/24	MTX-Naive
Fleischmann et al. (2019) (NCT02629159)	Worldwide	1,302	Upadacitinib	15 mg qd	MTX	MTX	12/26	MTX-IR
Genovese et al. (2018) (NCT02706847)	Worldwide	498	Upadacitinib	15、30 mg qd	csDMARD	csDMARD	12	bDMARD-IR
Burmester et al. (2018) (NCT02675426)	Worldwide	661	Upadacitinib	15、30 mg qd	csDMARD	csDMARD	12	bDMARD-IR
Kameda et al. (2020) (NCT02720523)	Japan	148	Upadacitinib	15、30 mg qd	csDMARD	csDMARD	12	csDMARD-IR
Zeng et al. (2021)(NCT02955212)	China, Brazil, South Korea	338	Upadacitinib	15 mg qd	csDMARD	csDMARD	12	csDMARD-IR
Westhovens et al. (2021) (NCT02886728)	Worldwide	1,042	Filgotinib	200 mg qd	Monotherapy/MTX	MTX	24	MTX-Naive
Atsumi et al. (2023) (NCT03025308)	Japan	60	Filgotinib	200 mg qd	Monotherapy/MTX	MTX	12/24	MTX-Naive
Kavanaugh et al. (2017) (NCT01894516)	Worldwide	141	Filgotinib	200 mg qd	MTX	MTX	12/24	MTX-IR
Genovese et al. (2019) (NCT02873936)	Worldwide	295	Filgotinib	200 mg qd	csDMARD	csDMARD	12/24	DMARD-IR
Combe et al. (2021) (NCT02889796)	Worldwide	950	Filgotinib	200 mg qd	csDMARD	csDMARD	12/24	MTX-IR
Westhovens et al. (2017) (NCT01888874)	Worldwide	172	Filgotinib	200 mg qd	csDMARD	csDMARD	12/24	MTX-IR
Tanaka et al. (2019) (NCT02308163)	Japan, Korea and Taiwan	308	Peficitinib	100、150 mg qd	Monotherapy	Placebo	12	DMARD-IR
Takeuchi et al. (2016) (NCT01649999)	Japan	169	Peficitinib	100、150 mg qd	Monotherapy	Placebo	12	DMARD-Naive
Genovese et al. (2017) (NCT01565655)	Worldwide	173	Peficitinib	100、150 mg qd	Monotherapy	Placebo	12	csDMARD-IR
Takeuchi et al. (2019) (NCT02305849)	Japan	519	Peficitinib	100、150 mg qd	MTX	MTX	12	MTX-IR
Kivitz et al. (2017) (NCT01554696)	Worldwide	234	Peficitinib	100、150 mg qd	MTX	MTX	12	MTX-IR

^a^ Same author and same year but different studies to distinguish.

N: Total number of patients; MTX: Methotrexate; DMARD: Disease-modifying antirheumatic drugs; csDMARD: conventional synthetic disease-modifying antirheumatic drugs; bDMARD: biological disease-modifying antirheumatic drugs; IR: inadequate response.

### 3.3 Literature quality assessment

**FIGURE 2:**
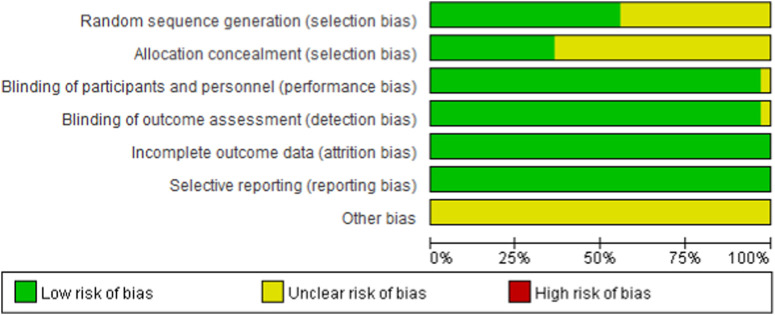
Risk of bias graph for all included studies.

**FIGURE 3:**
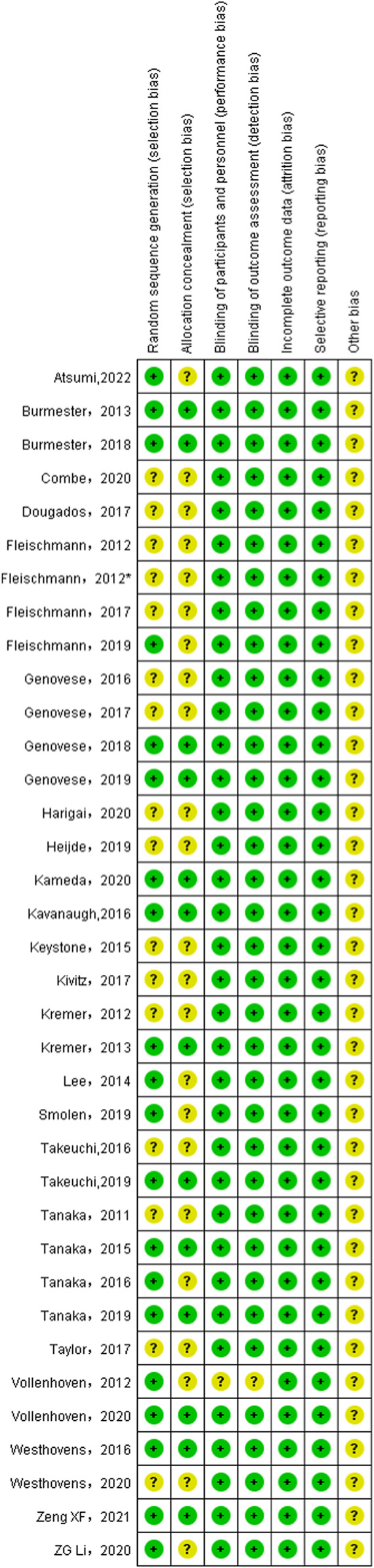
Risk of bias summary for all included study. *Same author and same year but different studies to distinguish.

As shown in Figure 2 and Figure 3, 20 studies detailed the sequence generation, marked as “low risk”, 13 studies specified the allocation concealment and marked as “low risk”. Two RCTs (Van Vollenhoven et al., 2012) were not known to have performance bias or detection bias both labeled as “unclear”. No attrition bias or reporting bias was found, so all RCTs were labeled as “low risk”. On the whole, the literature included in this study was of high quality.

### 3.4 Global inconsistency tests

The global inconsistency test of the response rates of ACR20, ACR50, ACR70 and DAS28(CRP) < 2.6 at 12 weeks showed that *p* values were 0.8769, 0.0585, 0.1834 and 0.7277 respectively. The response rates of ACR20, ACR50, ACR70 and DAS28(CRP) < 2.6 at 24 weeks were tested for global inconsistency, and the corresponding *p* values were 0.3240, 0.5319, 0.7385 and 0.0621. All *p* values were >0.05, indicating that the global inconsistency was not significant.

### 3.5 ACR and DAS28(CRP) < 2.6 response rates at 12 weeks

**FIGURE 4:**
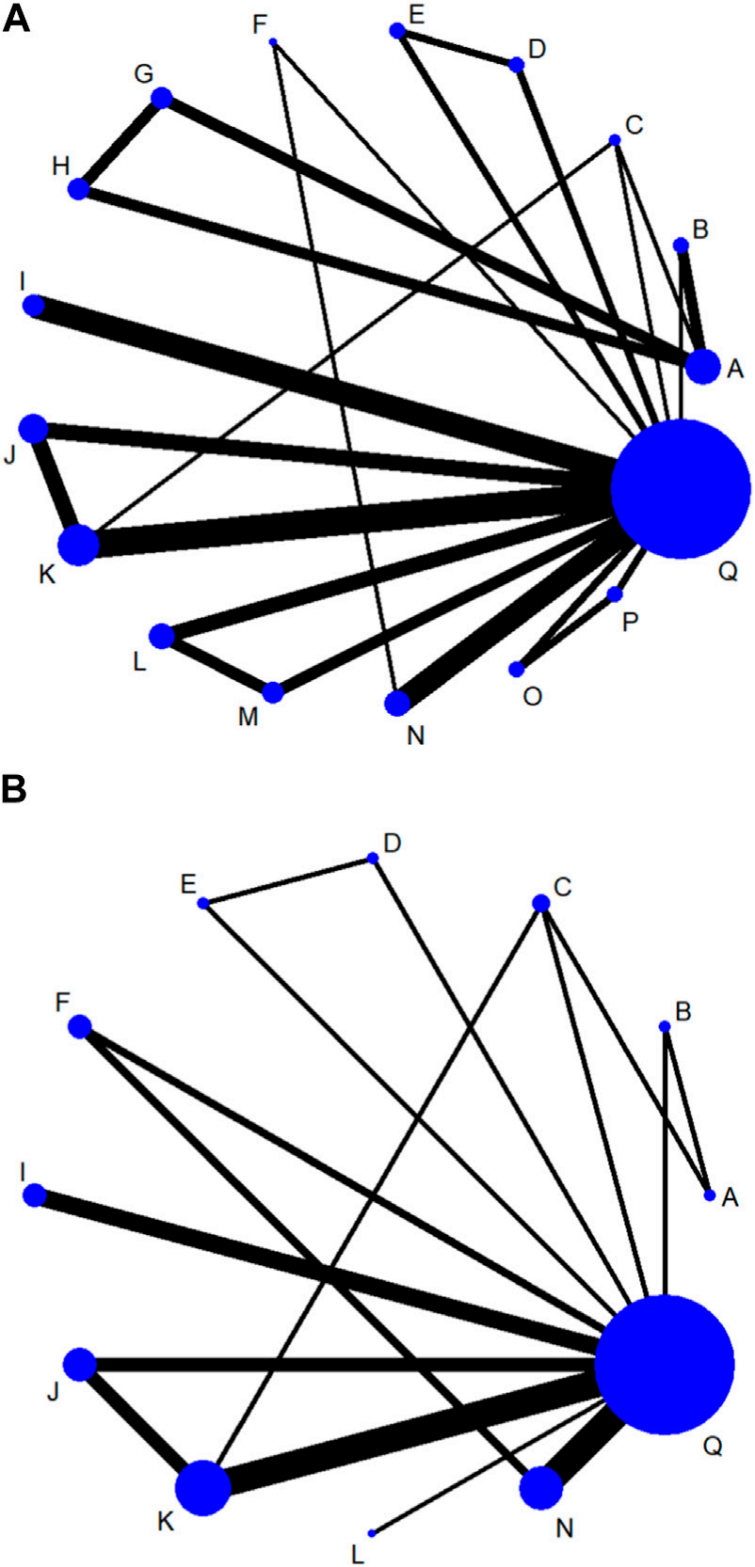
Network evidence plots of efficacy indicators at 12 week **(A)** or 24 week **(B)**. A: Placebo; B: tofacitinib 5 mg; C: baricitinib 4 mg; D: upadacitinib 15 mg; E: upadacitinib 30 mg; F: filgotinib 200 mg; G: peficitinib 100 mg; H: peficitinib 150 mg; I: tofacitinib 5 mg + csDMARD; J: baricitinib 2 mg + csDMARD; K: baricitinib 4 mg + csDMARD; L: upadacitinib 15 mg + csDMARD; M: upadacitinib 30 mg + csDMARD; N: filgotinib 200 mg + csDMARD; O: peficitinib 100 mg+csDMARD; P: peficitinib 150 mg + csDMARD; Q: Conventional synthetic disease-modifying antirheumatic drugs (csDMARD).

**FIGURE 5:**
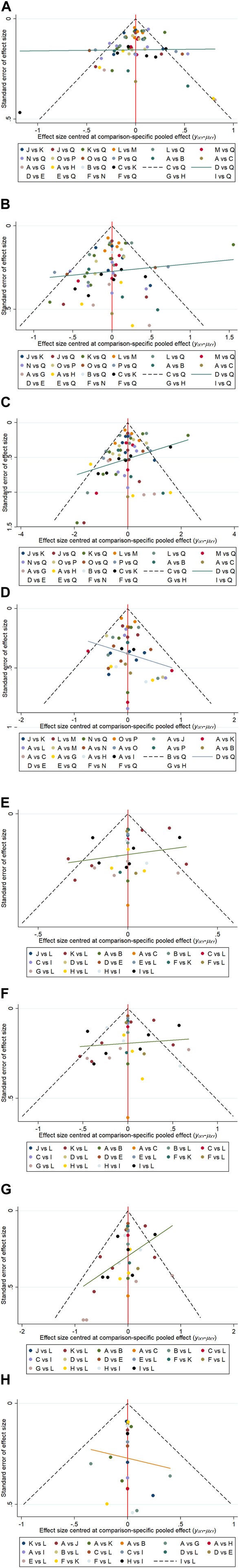
Funnel plots of ACR20 **(A)**, ACR50 **(B)**, ACR70 **(C)**, DAS28(CRP) < 2.6 **(D)** response rates at 12 week. Funnel plots of ACR20 **(E)**, ACR50 **(F)**, ACR70 **(G)** DAS28(CRP) < 2.6 **(H)** response rates at 24 week. A: Placebo; B: tofacitinib 5 mg; C: baricitinib 4 mg; D: upadacitinib 15 mg; E: upadacitinib 30 mg; F: filgotinib 200 mg; G: peficitinib 100 mg; H: peficitinib 150 mg; I: tofacitinib 5 mg + csDMARD; J: baricitinib 2 mg + csDMARD; K: baricitinib 4 mg + csDMARD; L: upadacitinib 15 mg + csDMARD; M: upadacitinib 30 mg + csDMARD; N: filgotinib 200 mg + csDMARD; O: peficitinib 100 mg + csDMARD; P: peficitinib 150 mg + csDMARD; Q: Conventional synthetic disease-modifying antirheumatic drugs (csDMARD).

(1) Network evidence plots. The bolder the lines in the diagram indicates the more the number of studies that directly compared the two interventions. The two interventions without wires are indirectly compared using network meta-analysis. The size of the dots indicates the size of the total sample size for the intervention. The results showed that the number of studies comparing placebo with baricitinib 4 mg + csDMARD was the largest, followed by comparing placebo with tofacitinib 5 mg + csDMARD(Figure 4A).
(2) SUCRA ranking. The higher the SUCRA value, the better the intervention effect. The probability ranking of treatment protocols based on SUCRA values is shown in Table 2. For the efficacy indicator ACR20, the rankings of the top three were as follows: upadacitinib 30 mg (90.9%)>upadacitinib 15 mg (88.0%)>tofacitinib 5 mg (84.8%). For the efficacy indicator of ACR50, the rankings of the top three were as follows: upadacitinib 30 mg (97.5%)>upadacitinib 15 mg (92.2%)>tofacitinib 5 mg (76.3%). The top three for the effectiveness indicator ACR70 were as follows: upadacitinib 30 mg (95.9%)>upadacitinib 15 mg (90.8%)>tofacitinib 5 mg (60.9%). The top three therapeutic regiments of the efficacy indicator were consistent and ranked the same, only the SUCRA values were different. In terms of DAS28(CRP) < 2.6, the rankings of the top three were as follows: upadacitinib 30 mg (93.8%)vupadacitinib 15 mg (84.7%)>peficitinib 150 mg + csDMARD (75.5%) (The SUCRA ranking diagram is shown in Supplementary Appendix S2)
(3) League plots. Network analysis was performed for all treatment regimens, and a total of 17 comparisons were statistically significant for ACR20. Among them, the RR and 95% CI of upadacitinib 30 mg were 3.08 [1.84,5.16] compared with placebo and were 1.82 [1.00,3.31] compared with csDMARD. The RR and 95% CI of the upadacitinib 15 mg were 2.98 [1.78,4.99] when compared to placebo. For ACR50, compared to placebo, the RR and 95% CI of upadacitinib 30 mg and upadacitinib 15 mg were 7.47 [4.31,12.96] and 6.45 [3.71,11.21], respectively. The RR and 95% CI of upadacitinib 30 mg compared with csDMARD were 3.69 [1.76,7.77]. For the indicator ACR70, the RR and 95% CI of upadacitinib 30 mg and upadacitinib 15 mg were 18.13 [5.24,62.68] and 14.05 [4.05,48.72], respectively. In terms of DAS28(CRP) < 2.6, the RR and 95% CI of upadacitinib 30 mg were 7.09 [3.14,16.02] compared with placebo and were 4.69 [3.43,6.41] compared with csDMARD. (See Supplementary Appendix S3 for the results)
(4) Publication bias. Funnel plot results for four efficacy indicators at 12 weeks showed that most scattering points were clustered at the top of the graph and distributed symmetrically. A small number of studies went beyond the funnel plot, suggesting possible publication bias or small sample effects in the included studies (Figure 5A, Figure 5B, Figures 5C,D).

**TABLE 2:** SUCRA ranking of ACR20、ACR50、ACR70、DAS28(CRP) < 2.6 response rates at 12 weeks.

Intervention measure	ACR20		ACR50		ACR70		DAS28(CRP) < 2.6	
	SUCRA/%	Rank	SUCRA/%	Rank	SUCRA/%	Rank	SUCRA/%	Rank
A	0.6	17	0.0	17	0.4	17	2.7	17
**B**	**84.8**	**3**	**76.3**	**3**	**60.9**	**3**	61.1	6
C	30.6	14	53.2	8	60.6	4	15.4	15
**D**	**88.0**	**2**	**92.2**	**2**	**90.8**	**2**	**84.7**	**2**
**E**	**90.9**	**1**	**97.5**	**1**	**95.9**	**1**	**93.8**	**1**
F	45.4	10	60.6	5	46.4	11	44.9	12
G	55.4	5	60.1	6	50.7	9	52.7	9
H	80.8	4	71.4	4	52.7	7	40.9	13
I	42.2	12	51.9	10	23.0	16	63.0	5
J	30.2	15	18.0	15	49.6	10	19.7	14
K	38.1	13	30.7	14	52.4	8	51.4	10
L	47.9	8	40.1	12	43.3	13	50.8	11
M	44.8	9	54.5	7	54.9	6	53.7	8
N	43.5	11	52.4	9	59.4	5	57.8	7
O	29.0	16	13.9	16	23.8	15	67.0	4
P	49.4	6	35.5	13	44.1	12	**75.5**	**3**
Q	48.4	7	41.7	11	40.9	14	14.8	16

The bold sections are the top three based on SUCRA, values. ACR:american college of rheumatology; SUCRA:surface under the cumulative ranking curve; A:Placebo; B:tofacitinib 5 mg; C:baricitinib 4 mg; D:upadacitinib 15 mg; E:upadacitinib 30 mg; F:filgotinib 200    mg; G:peficitinib 100 mg; H:peficitinib 150 mg; I:tofacitinib 5 mg + csDMARD; J:baricitinib 2 mg + csDMARD; K:baricitinib 4 mg + csDMARD; L:upadacitinib 15 mg + csDMARD; M:upadacitinib 30 mg + csDMARD; N:filgotinib 200 mg + csDMARD; O:peficitinib 100 mg + csDMARD; P:peficitinib 150 mg + csDMARD; Q:Conventional synthetic disease-modifying antirheumatic drugs(csDMARD).

### 3.6 ACR and DAS28(CRP) < 2.6 response rates at 24 weeks

(1) Network evidence plots. The results showed that the number of studies comparing placebo with baricitinib 4 mg + csDMARD was the largest (Figure 4B). The comparison of placebo and upadacitinib 15 mg + csDMARD was the least studied.
(2) SUCRA ranking. At 24 weeks, the largest SUCRA value was upadacitinib 15 mg + csDMARD (81.1%)for the efficacy indicator of ACR20. For the efficacy indicator of ACR50 and ACR70, the largest SUCRA value was baricitinib 4 mg + csDMARD (85.4%)and upadacitinib 30 mg (92.4%), respectively. All three efficacy measures with minimum SUCRA values were csDMARD (11.2%、15.9%、9.1%), except for placebo. As shown in Table 3. The largest SUCRA value was baricitinib 4 mg + csDMARD(95.7%)for the efficacy indicator of DAS28(CRP) < 2.6. (The SUCRA ranking diagram is shown in Supplementary Appendix S2).
(3) League plots. Look at Supplementary Appendix S3, the results of league plots showed that 19 comparisons, 14 comparisons and 26 comparisons were statistically significant for the three efficacy measures, respectively. Compared with placebo, the RR and 95% CI of upadacitinib 15 mg + csDMARD were 1.86 [1.50,2.31] and baricitinib 4 mg + csDMARD were 1.78 [1.59,2.00] for ACR20. Also compared with placebo, the RR and 95% CI of baricitinib 4 mg + csDMARD were 2.84 [2.23,3.60] and baricitinib 4 mg were 2.57 [1.67,3.96] for ACR50. For the indicator ACR70, the RR and 95% CI of upadacitinib 30 mg were 4.47 [3.18,6.27], while the RR and 95% CI of tofacitinib 5 mg + csDMARD were 4.13 [2.45,6.97] when compared with placebo. Compared with csDMARD, the RR and 95% CI of baricitinib 4 mg + csDMARD were 7.56 [2.37,24.07] and upadacitinib 15 mg + csDMARD were 6.24 [2.44,16.01] for the efficacy indicator of DAS28(CRP) < 2.6.
(4) Publication bias. Similar to the result of funnel plot analysis at 12 weeks, most scattering points were symmetrically distributed at 24 weeks. And there were possible publication bias and small sample effect due to the presence of few scattering points beyond the funnel plot (Figure 5E, Figure 5F, Figures 5G,H).

**TABLE 3:** SUCRA ranking of ACR20、ACR50、ACR70、DAS28(CRP) < 2.6 response rates at 24 weeks.

Intervention measure	ACR20		ACR50		ACR70		DAS28(CRP) < 2.6	
	SUCRA/%	Rank	SUCRA/%	Rank	SUCRA/%	Rank	SUCRA/%	Rank
A	0.0	12	0.0	12	0.0	12	9.6	11
B	47.3	7	55.5	5	46.4	8	29.6	10
C	61.6	6	70.6	2	61.2	4	54.8	6
D	73.2	3	55.0	6	78.2	3	33.6	9
E	71.1	4	53.8	7	**92.4**	**1**	47.1	7
F	25.9	10	38.0	10	20.2	10	45.5	8
I	69.4	5	68.4	4	85.2	2	63.9	4
J	43.3	8	38.9	9	57.3	7	60.1	5
K	76.6	2	**85.4**	**1**	59.4	5	**95.7**	**1**
L	81.1	**1**	68.7	3	57.4	6	91.3	2
N	39.3	9	49.8	8	33.1	9	65.5	3
Q	11.2	11	15.9	11	9.1	11	3.4	12

The bold sections are ranked first based on SUCRA, values. ACR:american college of rheumatology; SUCRA:surface under the cumulative ranking curve; A:Placebo; B:tofacitinib 5 mg; C:baricitinib 4 mg; D:upadacitinib 15 mg; E:upadacitinib 30 mg; F:filgotinib 200 mg; I:tofacitinib 5 mg + csDMARD; J:baricitinib 2 mg + csDMARD; K:baricitinib 4 mg + csDMARD; L:upadacitinib 15 mg + csDMARD; N:filgotinib 200 mg + csDMARD; Q:Conventional synthetic disease-modifying antirheumatic drugs(csDMARD).

### 3.7 Safety analysis

Based on SUCRA values, the lowest incidences of adverse event, serious adverse event and adverse event leading to discontinuation of study were placebo, baricitinib 2 mg + csDMARD, and filgotinib 200 mg, respectively (Table 4, Supplementary Appendix S4). Compared with placebo, upadacitinib 15 mg + csDMARD (RR 0.75, 95%CI 0.62–0.91) and upadacitinib 30 mg + csDMARD (RR 0.70, 95%CI 0.55–0.89) had a slightly higher incidence of infection. Peficitinib 150 mg + csDMARD (RR0.20, 95%CI 0.04–0.91) was associated with a higher incidence of serious infection when compared to placebo (Supplementary Appendix S5D,E). Based on SUCRA values, upadacitinib 30 mg had the best safety considering the incidence of malignancy. However, most of the differences among the treatment measures were not statistically significant (Supplementary Appendix S5). Due to the lack of relevant data such as MACE and VTE, the analysis of these two indicators based on SUCRA was only for reference. The analysis also showed that the difference was not statistically significant.

**TABLE 4:** SUCRA ranking of safety indicators.

Intervention measure		A	B	C	D	E	F	G	H	I	J	K	L	M	N	O	P	Q
Adverse Event (AE)	SUCRA/%	83.8	63.8	54.0	67.2	30.1	82.6	43.1	75.2	32.7	70.7	30.6	21.8	6.1	33.8	67.4	24.1	63.0
	Rank	1	7	9	6	14	2	10	3	12	4	13	16	17	11	5	15	8
Serious Adverse Event (SAE)	SUCRA/%	65.4	72.1	66.8	27.8	27.0	27.9	53.8	72.2	48.9	85.7	50.8	30.6	13.9	53.3	36.8	63.3	53.8
	Rank	5	3	4	15	16	14	7	2	11	1	10	13	17	9	12	6	8
AE leading to discontinua-tion of study	SUCRA/%	41.2	80.9	56.1	63.5	76.4	88.0	47.6	70.4	16.9	45.8	19.2	33.6	1.7	44.8	55.1	44.4	64.3
	Rank	13	2	7	6	3	1	9	4	16	10	15	14	17	11	8	12	5
Infection	SUCRA/%	89.4	66.9	72.3	74.6	47.3	53.6	20.9	22.1	17.4	69.6	39.7	41.8	31.6	49.2	48.8	48.7	56.3
	Rank	1	5	3	2	11	7	16	15	17	4	13	12	14	8	9	10	6
Serious infection	SUCRA/%	78.7	62.0	74.0	54.5	39.1	41.6	56.1	43.7	47.1	76.3	70.3	40.1	15.2	62.8	13.7	13.5	61.1
	Rank	1	6	3	9	14	12	8	11	10	2	4	13	15	5	16	17	7
Malignancy	SUCRA/%	47.6	39.8	62.5	36.0	77.4	76.5	23.8	49.9	16.3	65.3	33.7	53.1	30.4	66.5	47.6	69.0	54.8
	Rank	11	12	6	13	1	2	16	9	17	5	14	8	15	4	10	3	7
MACE	SUCRA/%	42.1	—	24.6	64.1	46.4	47.6	—	—	20.3	77.0	40.8	51.2	40.9	65.2	—	—	79.9
	Rank	8	—	11	4	7	6	—	—	12	2	10	5	9	3	—	—	1
VTE	SUCRA/%	73.0	—	—	28.5	32.9	49.7	—	—	—	—	—	58.6	62.8	65.5	—	—	29.0
	Rank	1	—	—	8	6	5	—	—	—	—	—	4	3	2	—	—	7

—: Data on the safety indicators of the relevant intervention measures were not published in the study and therefore could not be analyzed.

MACE: major adverse cardiovascular event; VTE: venous thromboembolic events; A:Placebo; B:tofacitinib 5 mg; C:baricitinib 4 mg; D:upadacitinib 15 mg; E:upadacitinib 30mg; F:filgotinib 200 mg; G:peficitinib 100 mg; H:peficitinib 150 mg; I:tofacitinib 5 mg + csDMARD; J:baricitinib 2 mg + csDMARD; K:baricitinib 4 mg + csDMARD; L:upadacitinib 15 mg + csDMARD; M:upadacitinib 30 mg + csDMARD; N:filgotinib 200 mg + csDMARD; O:peficitinib 100 mg + csDMARD; P:peficitinib 150 mg + csDMARD; Q:Conventional synthetic disease-modifying antirheumatic drugs(csDMARD).

## 4 Discussion

RA is currently managed from three perspectives according to the treatment regimen recommended by ACR and EULAR: 1) symptomatic treatment, including Nonsteroidal Antiinflammatory Drugs (NSAIDs) and Glucocorticosteroid (GCs); 2) disease modifying management, including DMARDs; 3) treatment of comorbidity, such as interstitial lung disease (Smolen et al., 2020; Fraenkel et al., 2021). DMARDs are drugs that promote remission by inhibiting autoimmune activity and delaying or preventing joint degeneration. In consideration of DMARDs are slow-acting drugs that generally take 6 weeks to 6 months to take effect, treatment should be started as soon as possible, as early implementation leads to better outcomes. DMARDs have been classified as conventional synthetic DMARDs (csDMARDs), biologic DMARDs (bDMARDs) and targeted synthetic DMARDs (tsDMARDs) (Monti et al., 2017). The 2021 ACR RA Treatment Guidelines updated the recommendation to use JAK inhibitors when csDMARDs are ineffective (Fraenkel et al., 2021). JAK inhibitors belongs to tsDMARDs. According to its selectivity, it can be divided into two groups: the first group is a low-selective inhibitor that inhibits a variety of cytokines signaling, and the second generation can selectively inhibit the signaling process. JAK inhibitors are metabolized and excreted through liver and kidney making them vulnerable to drug interactions and changes in blood drug concentrations caused by liver or kidney impairment. Since the pharmacological activity of JAK inhibitors is based on competitive binding to ATP binding sites, even though they are highly selective, they may not retain their selectivity if their blood concentrations are too high (Kubo et al., 2023). The five JAK inhibitors approved for the treatment of RA were initiated primarily based on the results of Phase III (P3) trials. Not only its clinical efficacy, but also its inhibition of the progression of joint injury was evaluated in these clinical trials, and all JAK inhibitors showed favorable results.

This NMA focused on comparative efficacy of the five JAK inhibitors currently approved worldwide (tofacitinib 5 mg,bid; baricitinib 2 mg or 4 mg, qd; upadacitinib 15 mg or 30 mg, qd; filgotinib 200 mg,qd; peficitinib 100 mg or 150 mg,qd), including monotherapy and combination therapy. The results showed that at 12 weeks, there was a lack of studies on baricitinib 2 mg monotherapy. SUCRA ranking of the three efficacy indicators indicated that upadacitinib 30 mg monotherapy had the best efficacy. The second was upadacitinib 15 mg. At 24 weeks, there was a lack of studies on baricitinib 2 mg, peficitinib100 mg, peficitinib 150 mg, upadacitinib 30 mg + csDMARD, peficitinib100 mg + csDMARD, and peficitinib 150 mg + csDMARD. According to the SUCRA ranking of ACR20, ACR50, ACR70 and DAS28(CRP) < 2.6, the results showed that upadacitinib 15 mg + csDMARD, baricitinib 4 mg + csDMARD, upadacitinib 30 mg and baricitinib 4 mg + csDMARD were the most effective, respectively. No matter 12 weeks or 24 weeks, treatment with JAK inhibitors monotherapy or combination therapy was both more effective than placebo or csDMARD. The ACR response rate of monotherapy at 24 weeks was lower than that of monotherapy at 12 weeks. The combination treatment showed better efficacy outcomes at 24 weeks than at 12 weeks. The results suggested that combination therapy may be more suitable for long-term treatment. This study is the first to use a NMA to compare the effectiveness of five approved JAK inhibitors as monotherapy or combination therapy in the treatment of RA, providing references for the clinical use of JAK inhibitors and the design of subsequent related studies.

In summary, upadacitinib 15 mg and upadacitinib 30 mg showed better efficacy at 12 weeks. At 24 weeks, upadacitinib 15 mg + csDMARD and baricitinib 4 mg + csDMARD showed better efficacy. In a systematic review and NMA, three JAK inhibitors (tofacitinib, baricitinib, upadacitinib) were evaluated in comparison to ACR response rates in patients treated with moderate-to-severe csDMARD-IR as monotherapy or in combination. Upadacitinib 15 mg displayed more remarkable efficacy either as monotherapy or in combination (Pope et al., 2020). In another NMA, the efficacy of three JAK inhibitors (tofacitinib, baricitinib, upadacitinib)and bDMARDs were compared in patients with RA who had an inadequate response to at least one DMARD. Upadacitinib, tocilizumab and certolizumab showed relatively good efficacy in three efficacy outcomes, such as ACR20, DAS28, and HAQ-DI (Weng et al., 2021). In RA patients with an inadequate response to cs-or b-DMARDs, upadacitinib 15 mg + MTX and upadacitinib 30 mg + MTX were more effective than tofacitinib 10 mg + MTX and tofacitinib 5 mg + MTX without any significant risk of serious adverse events in another NMA, which aims to compare the efficacy and safety of tofacitinib and upadacitinib in combination with MTX (Song et al., 2019). Similarly, in another NMA, the relative efficacy of four JAK inhibitors (tofacitinib, baricitinib, upadacitinib, and filgotinib) and adalimumab in MTX-IR RA patients was evaluated. Baricitinib 4 mg + MTX and upadacitinib 15 mg + MTX resulted in significantly improved ACR response rates (Lee and Song, 2020). These data suggested that upadacitinib had a therapeutic advantage, which was basically consistent with the conclusions of this NMA study. In subgroup studies of treatment duration, reaching the efficacy endpoint at 24 weeks was more recommended for combination therapy. It was demonstrated that combined therapy was more helpful to maintain long-term curative effect.

With the exception of ORAL surveillance, most phase II and III registries, meta-analyses, and post-analysis clinical trial programs have not shown an increased incidence of ASCVD (arteriosclerotic cardiovascular disease), such as MACE, and VTE associated with JAK inhibitors. In terms of infections, treatment with JAK inhibitors was associated with an increased risk of herpes zoster, but not with severe infections (Winthrop, 2017; Atzeni et al., 2021). Although this study also showed no statistical difference in safety, it is necessary to be vigilant about the occurrence of adverse reactions, especially in high-risk patients. Extensive post-analysis of data from the ORAL surveillance trial had been performed to understand exactly which patients were most at risk for malignancy, MACE, VTE, and death, and had yielded important observations. Firstly, these events were more likely to occur in older patients (≥65 years of age) with a history of smoking. Secondly, infections were more likely to occur in older patients, smokers, and those with active disease. Thirdly, the increased incidence of malignancy was associated with a high risk of MACE or a history of ASCVD, the risk of developing another MACE was also highest and should be appropriately treated with a statin. Finally, RA patients were at an increased risk of developing VTE if they had a prior history of VTE, persistently active RA, older or obese, or taking hormone replacement therapy. These adverse events occurred almost exclusively in high-risk patients (≥65 years of age or former smokers) and rarely in patients without these risk factors (low-risk patients) (Szekanecz et al., 2024). However, it was not conclusively known whether treatment with JAK inhibitors was directly related to the occurrence of these adverse events.

There are still some limitations of our study. First, both DMARD-naive and DMARD-IR moderate-to-severe RA patients were included in the analysis, which allowed us to include more relevant studies. But it may also increase the risk of bias. However, patient baseline characteristics and disease activity were balanced to reduce this bias. Second, we selected all the recommended doses of the five JAK inhibitors for analysis, but non-recommended doses are also common in clinical practice. More commonly used clinical doses can be selected for subsequent analysis. Third, due to article length limitations, some other efficacy endpoints were not included in the analysis. For example, Clinical Disease Activity Index (CDAI), Simplified Disease Activity Index (SDAI), and Health Assessment Questionnaire-Disability Index (HAQ-DI), etc. It is worth mentioning that in clinical practice, absolute changes in DAS28 and patients’ subjective perceptions, such as pain and fatigue, are often used to confirm whether early treatment in RA patients is effective. The safety of the five JAK inhibitors will not change but well better documented in the future. Fourth, due to the lack of relevant clinical studies, the included studies were limited and there may be a risk of bias. Future studies of baricitinib 2 mg monotherapy and peficitinib for the 24-week treatment of RA can be advanced. And most studies are from RCT with placebo or csDMARD controls, head-to-head comparisons between JAK inhibitors are still lacking. Future RCT should focus more on direct comparisons between JAK inhibitors. Long-term safety data and real-world data from patients may be more clinically significant. This will continue to be determined in future studies. Finally, although upadacitinib is recommended in this study, tofacitinib and baricitinib are more commonly used than upadacitinib in clinical practice for the earlier approve and more extensive safety data. Clinicians need to consider specific clinical characteristics to make the best choice.

Nevertheless, this study also has some advantages. First of all, we conducted a comprehensive search of existing databases, which allowed us to conduct a more complete analysis of the existing research evidence. Secondly, the RCTs included in this study were of high quality and consistent. A total of nearly 15,000 patients were included. Thirdly, we analyzed and compared the five JAK inhibitors approved for RA treatment and the approved doses, and this is the first NMA to include all five JAK inhibitors simultaneously. Important safety indicators such as serious infection, malignancy, MACE, and VTE for five JAK inhibitors were also included in the analysis. Finally, simultaneous comparison of different treatment regimens was conducted including monotherapy and combination regimens, despite the direct head-to-head comparisons was lack. Efficacy was ranked by SUCRA values to increase the reliability of statistical results with more accurate data.

## 5 Conclusion

In conclusion, this NMA study showed that all JAK inhibitors performed better than placebo in moderate-to-severe active RA patients. At 24 weeks, all JAK inhibitors also performed better than csDMARD. Analysis of three efficacy indicators showed that upadacitinib 30 mg and upadacitinib 15 mg at 12 weeks and upadacitinib 15 mg + csDMARD and baricitinib 4 mg + csDMARD at 24 weeks were recommended treatment options with relatively good efficacy. Safety indicators evaluation showed no statistical difference, but still need to pay attention to adverse events, especially in high-risk RA patientes with risk factors. Based on the conclusions of this study, clinicians can select the most appropriate drug regimen for patients with RA according to their specific disease characteristics and drug tolerance.
