# Supplementary material for: Comparative efficacy of five approved Janus kinase inhibitors as monotherapy and combination therapy in patients with moderate-to-severe active rheumatoid arthritis: a systematic review and network meta-analysis of randomized controlled trials
Source: Front Pharmacol. 2024 Apr 24;15:1387585. doi: 10.3389/fphar.2024.1387585 (PMC11080655; doi:10.3389/fphar.2024.1387585)
Supplement: Supplementary file 1 [file DataSheet1.docx]

**Supplementary appendix 1. Participant baseline characteristics of included RCTs**

| Author, year | Intervention measure | Age，mean  (years) | Course of disease  (years) | Female, N(%) | DAS28(CRP) |
| --- | --- | --- | --- | --- | --- |
| Fleischmann,2012 | Tofacitinib 5mg bid  Placebo | 52.2  49.7 | 8.0  7.7 | 207(85.2)  105 (86.1) | 5.68  5.56 |
| Tanaka,2015 | Tofacitinib 5mg bid  Placebo | 52.6  53.3 | 11.0  6.4 | 44 (84.6)  43 (82.7) | 6.41  5.83 |
| Fleischmann,2012* | Tofacitinib 5mg bid  Placebo | 54  53 | 8.1  10.8 | 43 (87.8)  52 (88.1) | 5.6  5.6 |
| Lee,2014 | Tofacitinib 5mg bid  MTX | 50.3  48.8 | 2.9  2.7 | 286(76.7)  145(78.0) | 6.6  6.6 |
| Vollenhoven,2012 | Tofacitinib 5mg bid+MTX  MTX | 53.0  53.7 | 7.6  8.0 | 174 (85.3)  82(75.9) | 5.4  5.5 |
| Heijde,2019 | Tofacitinib 5mg bid+MTX  MTX | 53.7  52.3 | 8.9  9.2 | 269(83.8)  68(85.7) | 6.33  6.27 |
| Burmester,2013 | Tofacitinib 5mg bid+MTX  MTX | 55.4  54.4 | 13.0  11.3 | 113 (85.0)  106 (80.3) | 5.4  5.4 |
| Kremer,2013 | Tofacitinib 5mg bid+MTX  MTX | 50.8  52.1 | 9.5  9.9 | 264(83.8)  123(77.4) | 6.27  6.29 |
| Tanaka,2011 | Tofacitinib 5mg bid+MTX  MTX | 50.0  50.6 | 8.3  8.4 | 22 (81.5)  25 (89.3) | 5.0  4.9 |
| Kremer,2012 | Tofacitinib 5mg bid+MTX  MTX | 52  53 | 9.0  9.2 | 57 (80.3)  56 (81.2) | 5.1  5.3 |
| Harigai,2020 | Baricitinib 4mg qd  Placebo | 56.8  55.2 | 5.6  7.7 | 29(90.6)  22(71.0) | 4.58  5.05 |
| Fleischmann,2017 | Baricitinib 4mg qd  Baricitinib 4mg qd+MTX  MTX | 49  51 | 1.3  1.3 | 156 (73)  148 (70) | 5.9  5.9 |
| Taylor,2017 | Baricitinib 4mg qd+MTX  MTX | 54  53 | 10  10 | 375 (77)  382 (78) | 5.8  5.7 |
| Keystone,2015 | Baricitinib 2mg qd+MTX  Baricitinib 4mg qd+MTX  MTX | 51  53  49 | 5.5  5.3  5.4 | 44(85)  37(71)  85(87) | 5.4  5.3  5.5 |
| Tanaka,2016 | Baricitinib 2mg qd+MTX  Baricitinib 4mg qd+MTX  MTX | 56.1  57.5  51.1 | 6.32  5.86  5.06 | 88 (21)  79 (19)  80 (39) | 4.94  4.96  4.72 |
| ZG Li,2020 | Baricitinib 4mg qd+MTX  MTX | 49.5  48.9 | 10.7  9.1 | 127 (87.6)  106 (73.1) | 5.9  6.0 |
| Genovese,2016 | Baricitinib 2mg qd+csDMARD  Baricitinib 4mg qd+csDMARD  csDMARD | 55  56  56 | 14  14  14 | 137 (79)  149 (84)  145 (82) | 6.0  5.9  5.9 |
| Dougados,2017 | Baricitinib 2mg qd+csDMARD  Baricitinib 4mg qd+csDMARD  csDMARD | 52  52  51 | 8  8  7 | 184 (80)  187 (82)  189 (83) | 5.6  5.6  5.5 |
| Smolen,2019 | Upadacitinib 15mg qd  Upadacitinib 30mg qd  MTX | 54.5  53.1  55.3 | 7.5  6.5  5.8 | 174 (80)  170 (79)  179 (83) | 5.6  5.6  5.6 |
| Vollenhoven,2020 | Upadacitinib 15mg qd  Upadacitinib 30mg qd  MTX | 51.9  54.9  53.3 | 2.9  2.8  2.6 | 241 (76.0)  240 (76.4)  240 (76.4) | 5.9  5.8  5.9 |
| Fleischmann,2019 | Upadacitinib 15mg qd+MTX  MTX | 54  54 | 8  8 | 521 (80)  512 (79) | 5.8  5.8 |
| Genovese,2018 | Upadacitinib 15mg qd+csDMARD  Upadacitinib 30mg qd+csDMARD  csDMARD | 56.3  57.3  57.6 | 12.4  12.7  14.5 | 137 (84)  138 (84)  143 (85) | 5.9  5.8  5.8 |
| Burmester,2018 | Upadacitinib 15mg qd+csDMARD  Upadacitinib 30mg qd+csDMARD  csDMARD | 55.3  55.8  56.0 | 7.3  7.3  7.2 | 182 (82)  172 (79)  166 (75) | 5.7  5.7  5.6 |
| Kameda,2020 | Upadacitinib 15mg qd+csDMARD  Upadacitinib 30mg qd+csDMARD  csDMARD | 56.0  54.7  54.3 | 2.9  2.8  2.1 | 36 (73.5)  43 (86.0)  42 (85.7) | 5.1  5.0  5.2 |
| Zeng XF,2021 | Upadacitinib 15mg qd+csDMARD  csDMARD | 51.7  51.7 | 7.2  7.5 | 135 (79.9)  139 (82.2) | 5.6  5.6 |
| Westhovens,2020  （NCT02886728） | Filgotinib 200mg qd  Filgotinib 200mg qd+MTX  MTX | 52  53  53 | 2.6  1.9  2.3 | 166 (79)  325 (78)  312 (75) | 5.8  5.7  5.7 |
| Atsumi,2022  （NCT03025308） | Filgotinib 200mg qd  Filgotinib 200mg qd+MTX  MTX | 52  52  58 | 1.8  1.8  1.8 | 5 (55.6)  13 (72.2)  4 (44.4) | 2.3  1.5  2.6 |
| Kavanaugh,2016  （NCT01894516） | Filgotinib 200mg qd+MTX  MTX | 52  52 | 9  10 | 60 (87.0)  56 (77.8) | 6.09  6.22 |
| Genovese,2019  （NCT02873936） | Filgotinib 200mg qd+csDMARD  csDMARD | 56  56 | 9.8  9.9 | 120 (81.6)  121 (81.8) | 5.9  5.9 |
| Combe,2020  （NCT02889796） | Filgotinib 200mg qd+csDMARD  csDMARD | 52  53 | 7.3  7.3 | 379 (79.8)  391 (82.3) | 5.8  5.7 |
| Westhovens,2016  （NCT01888874） | Filgotinib 200mg qd+csDMARD  csDMARD | 55  52 | 9  8 | 74 (86.0)  70 (81.4) | 6.22  5.98 |
| Tanaka,2019  （NCT02308163） | Peficitinib 100mg qd  Peficitinib 150mg qd  Placebo | 54.1  55.0  56.3 | 8.75  10.39  6.98 | 77 (74.0)  78 (76.5)  73 (72.3) | 5.29  5.41  5.43 |
| Takeuchi,2016  （NCT01649999） | Peficitinib 100mg qd  Peficitinib 150mg qd  Placebo | 52.1  51.6  54.2 | 7.58  6.95  6.92 | 42 (76.4)  51 (87.9)  43 (76.8) | 5.34 5.41  5.10 |
| Genovese,2017  （NCT01565655） | Peficitinib 100mg qd  Peficitinib 150mg qd  Placebo | 54.9  54.4  52.7 | 11.0  10.5  9.8 | 51 (87.9)  50 (78.1)  42 (82.4) | 5.7  5.9  5.9 |
| Takeuchi,2019  （NCT02305849） | Peficitinib 100mg qd  Peficitinib 150mg qd  Placebo | 58.5  56.2  55.3 | 4.41  4.37  4.30 | 118 (67.8)  125 (71.8)  121 (71.2) | 5.21  5.36  5.41 |
| Kivitz,2017  （NCT01554696） | Peficitinib 100mg qd+MTX  Peficitinib 150mg qd+MTX  MTX | 54.5  54.2  52.6 | 7.5  7.3  7.2 | 68 (81.0)  64 (82.1)  63 (87.5) | 5.6  5.6  5.4 |

*Same author and same year but different studies to distinguish.

N: Number of patients；MTX：Methotrexate；csDMARD：conventional synthetic disease-modifying antirheumatic drugs；DAS28(CRP)：28-joint disease activity score based on CRP

A


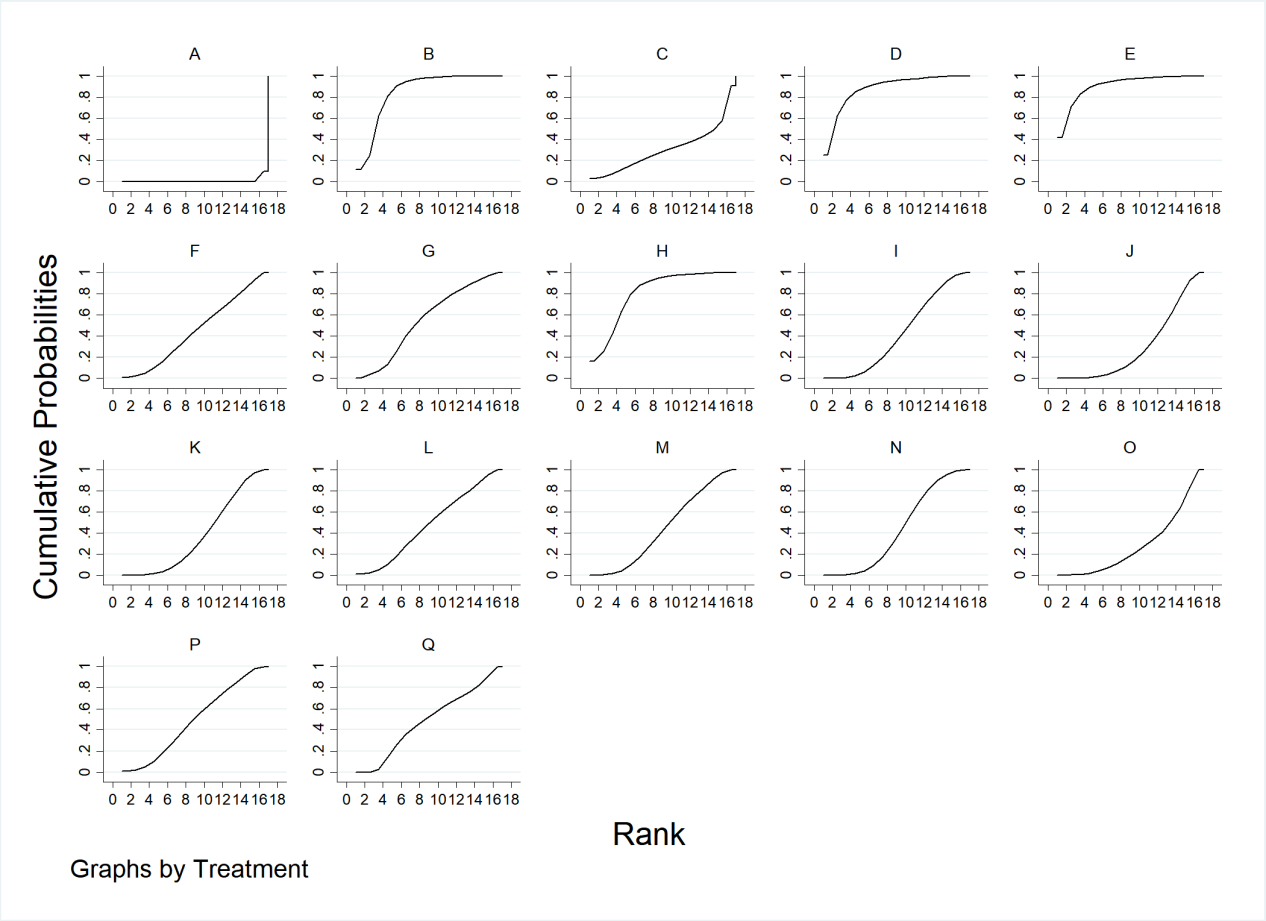


B


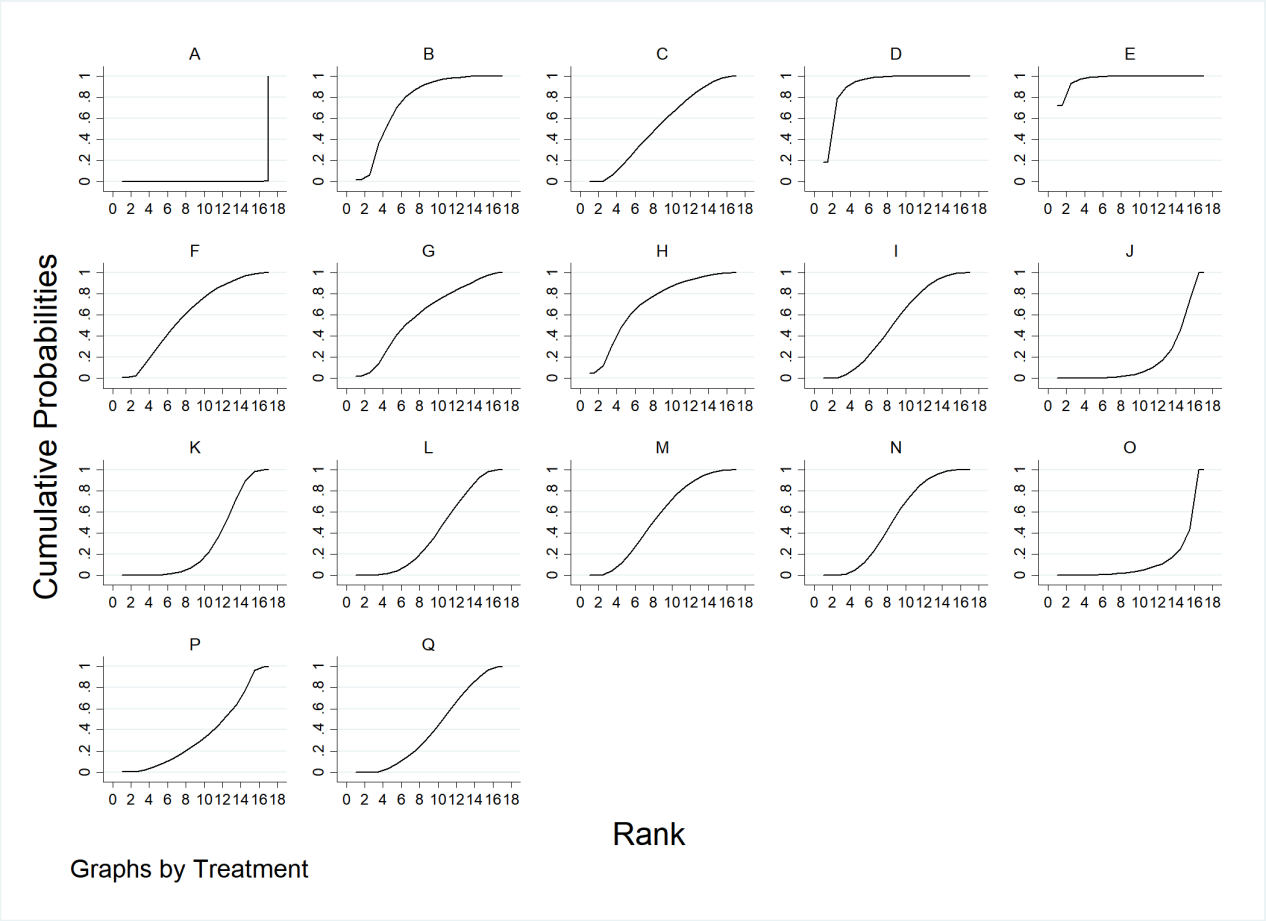


C


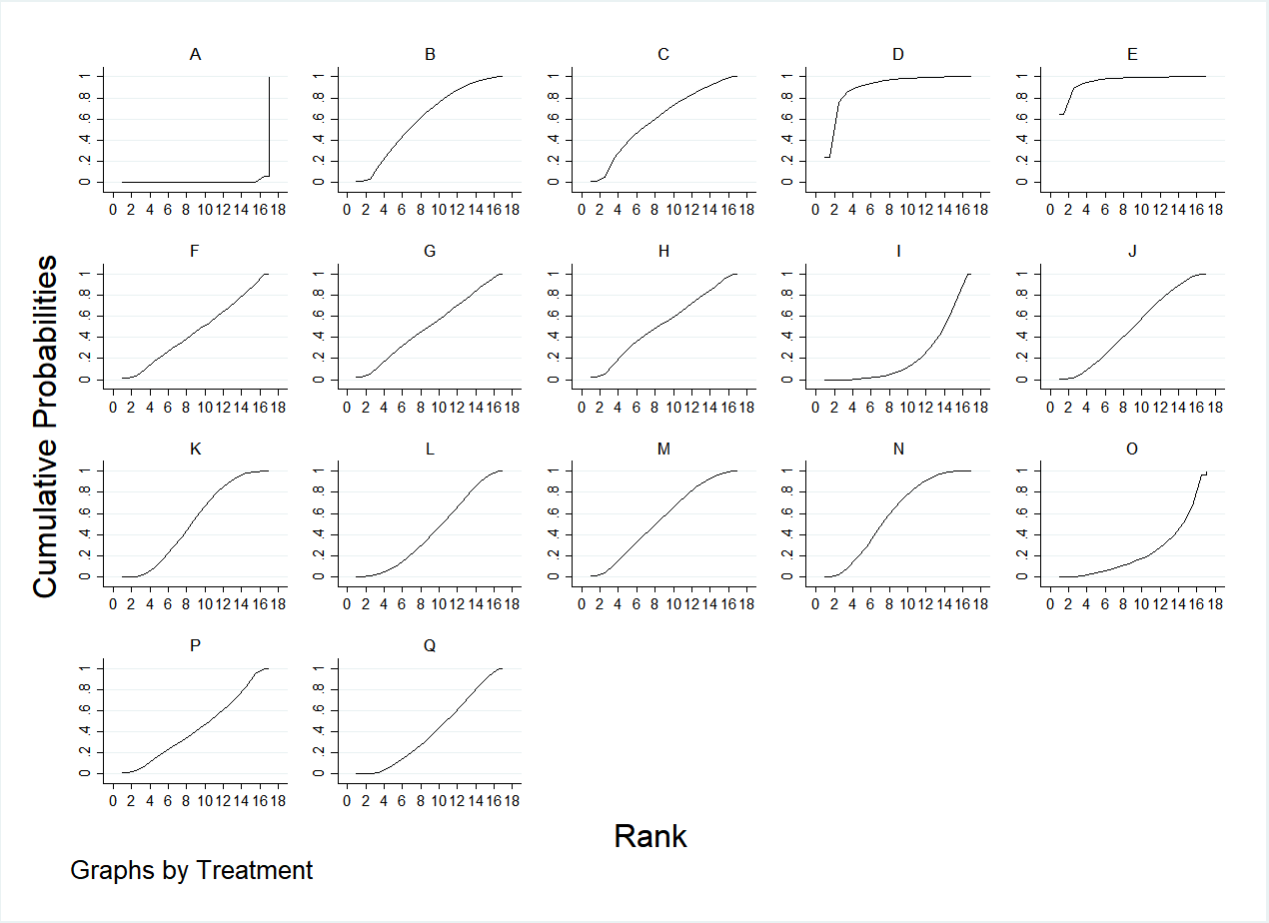


D


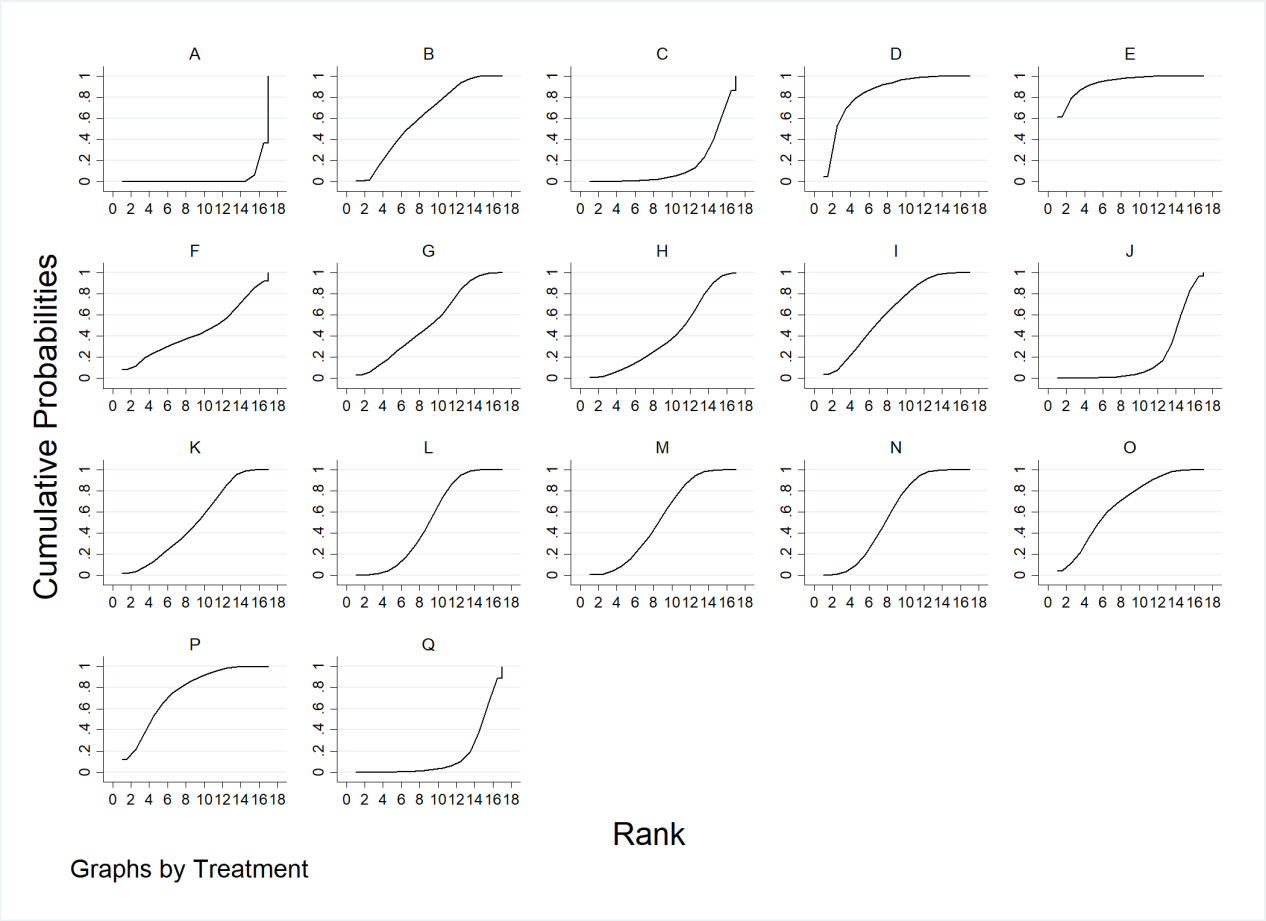


E


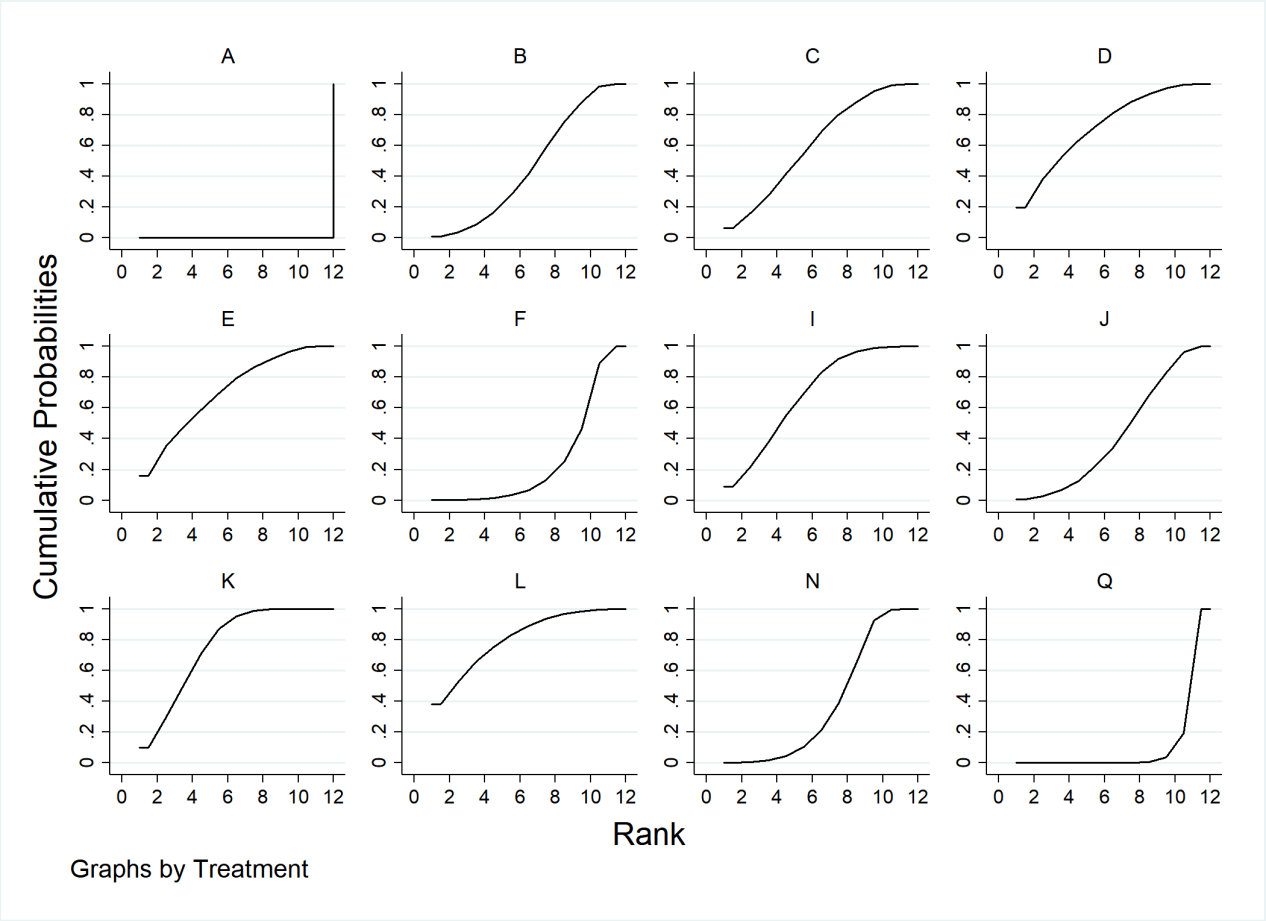


F
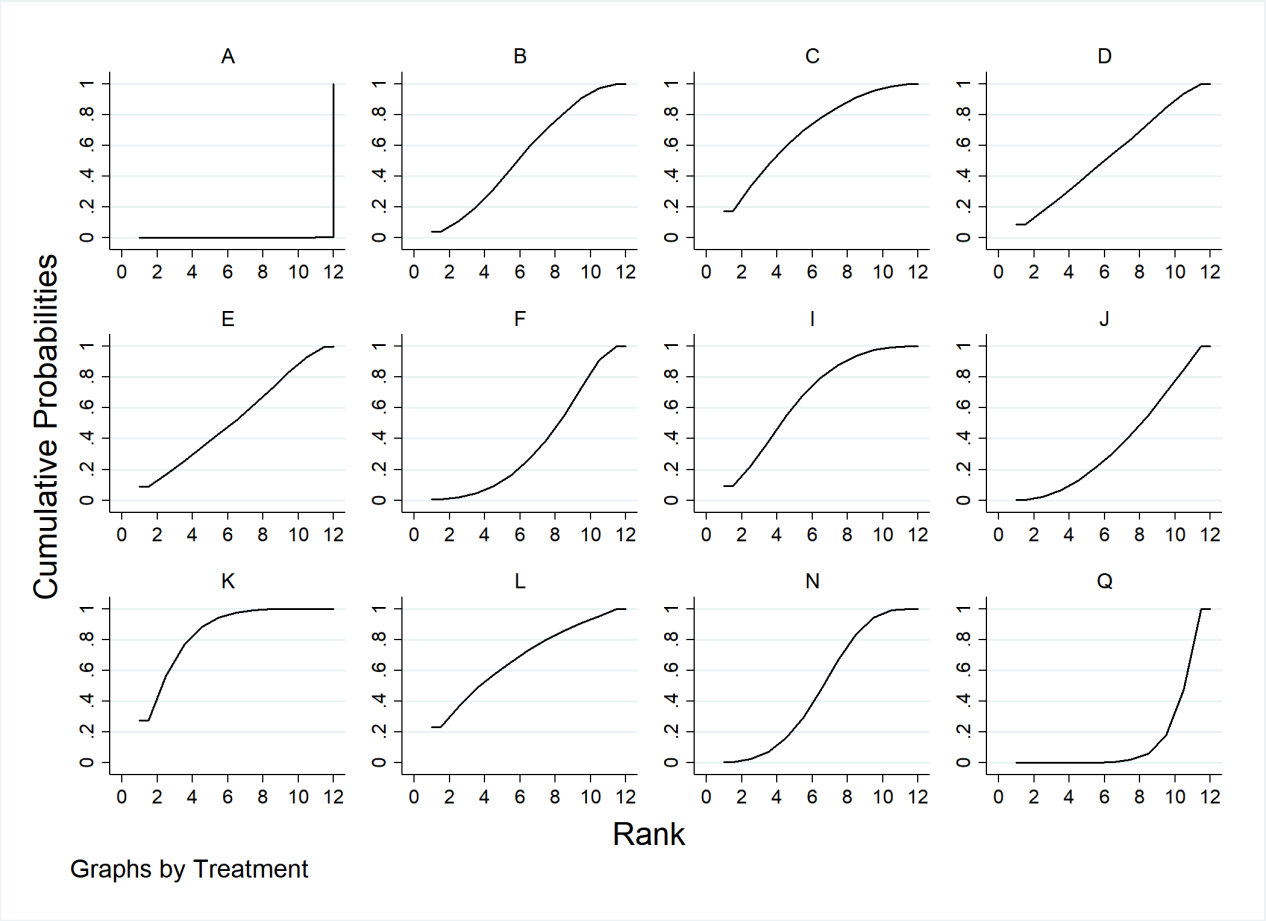


G
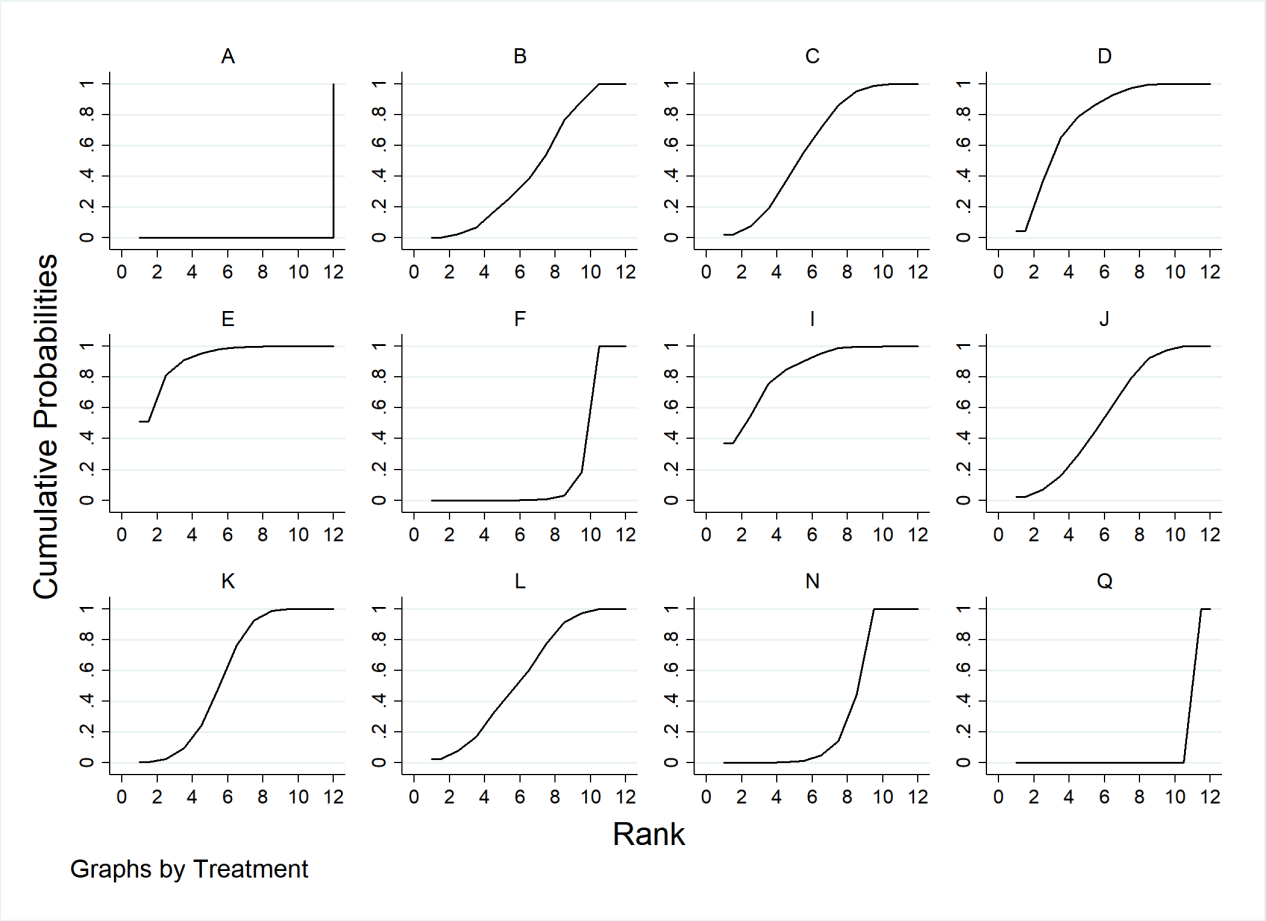


H


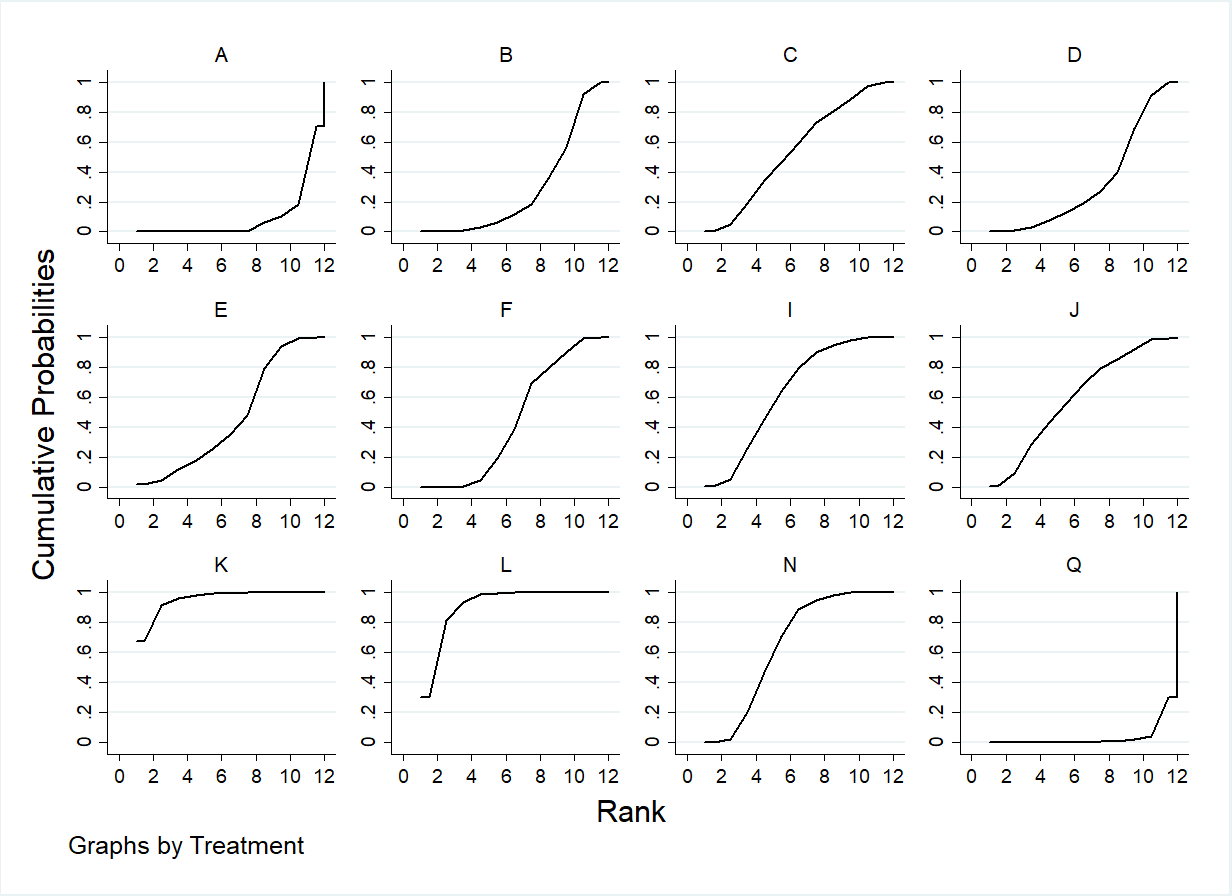


**Supplementary appendix 2. SUCRA ranking of ACR20（A）、ACR50（B）、ACR70（C）、DAS28(CRP)＜2.6（D） response rates at 12 week. SUCRA ranking of ACR20（E）、ACR50（F）、ACR70（G）、DAS28(CRP)＜2.6（H） response rates at 24 week.** ACR:American College of Rheumatology; SUCRA:surface under the cumulative ranking curve; A:Placebo; B:tofacitinib 5mg; C:baricitinib 4mg; D:upadacitinib 15mg; E:upadacitinib 30mg; F:filgotinib 200mg; G:peficitinib 100mg; H:peficitinib 150mg; I:tofacitinib 5mg+csDMARD; J:baricitinib 2mg+csDMARD; K:baricitinib 4mg+csDMARD; L:upadacitinib 15mg+csDMARD; M:upadacitinib 30mg+csDMARD; N:filgotinib 200mg+csDMARD; O:peficitinib 100mg+csDMARD; P:peficitinib 150mg+csDMARD; Q:Conventional synthetic disease-modifying antirheumatic drugs(csDMARD)

A

| E | 0.97 (0.78,1.21) | 0.87 (0.58,1.31) | 0.82 (0.45,1.50) | 0.67 (0.36,1.23) | 0.63 (0.35,1.15) | **0.64 (0.51,0.81)** | 0.63 (0.34,1.15) | 0.61 (0.35,1.08) | 0.61 (0.33,1.14) | 0.61 (0.36,1.05) | 0.61 (0.35,1.05) | 0.59 (0.34,1.03) | 0.52 (0.22,1.22) | **0.56 (0.32,0.98)** | 0.55 (0.30,1.00) | **0.32 (0.19,0.54)** |
| --- | --- | --- | --- | --- | --- | --- | --- | --- | --- | --- | --- | --- | --- | --- | --- | --- |
| 1.03 (0.83,1.29) | D | 0.90 (0.60,1.35) | 0.85 (0.47,1.55) | 0.69 (0.38,1.27) | 0.65 (0.36,1.18) | **0.67 (0.53,0.84)** | 0.65 (0.36,1.19) | 0.64 (0.36,1.12) | 0.63 (0.34,1.18) | 0.63 (0.37,1.08) | 0.63 (0.36,1.09) | 0.61 (0.35,1.06) | 0.54 (0.23,1.26) | 0.58 (0.33,1.01) | 0.57 (0.31,1.04) | 0.34 (0.20,0.56) |
| 1.15 (0.76,1.72) | 1.11 (0.74,1.67) | B | 0.94 (0.61,1.47) | 0.77 (0.49,1.20) | 0.72 (0.47,1.12) | 0.74 (0.53,1.03) | 0.72 (0.46,1.13) | 0.71 (0.48,1.04) | 0.70 (0.44,1.12) | 0.70 (0.49,1.00) | 0.70 (0.48,1.01) | **0.68 (0.46,0.98)** | 0.60 (0.28,1.26) | **0.65 (0.44,0.94)** | **0.63 (0.41,0.98)** | **0.37 (0.27,0.51)** |
| 1.22 (0.67,2.22) | 1.18 (0.64,2.15) | 1.06 (0.68,1.65) | H | 0.81 (0.64,1.03) | 0.77 (0.50,1.19) | 0.78 (0.45,1.36) | 0.76 (0.49,1.19) | 0.75 (0.51,1.10) | 0.75 (0.47,1.19) | 0.74 (0.52,1.06) | 0.74 (0.51,1.07) | 0.72 (0.49,1.04) | 0.63 (0.30,1.33) | 0.68 (0.47,1.00) | 0.67 (0.43,1.04) | **0.48 (0.35,0.67)** |
| 1.49 (0.82,2.74) | 1.45 (0.79,2.65) | 1.30 (0.83,2.04) | 1.23 (0.97,1.55) | G | 0.94 (0.60,1.47) | 0.96 (0.55,1.68) | 0.94 (0.60,1.48) | 0.92 (0.62,1.37) | 0.92 (0.57,1.47) | 0.91 (0.63,1.32) | 0.91 (0.62,1.32) | 0.88 (0.60,1.29) | 0.78 (0.37,1.65) | 0.84 (0.57,1.24) | 0.82 (0.53,1.29) | **0.53 (0.38,0.74)** |
| 1.59 (0.87,2.88) | 1.53 (0.84,2.79) | 1.38 (0.89,2.14) | 1.30 (0.84,2.02) | 1.06 (0.68,1.65) | P | 1.02 (0.59,1.77) | 1.00 (0.64,1.54) | 0.97 (0.67,1.43) | 0.97 (0.62,1.54) | 0.97 (0.68,1.37) | 0.96 (0.67,1.38) | 0.93 (0.65,1.35) | 0.82 (0.39,1.73) | 0.89 (0.62,1.29) | 0.87 (0.67,1.14) | **0.52 (0.38,0.71)** |
| **1.55 (1.23,1.96)** | **1.50 (1.19,1.90)** | 1.35 (0.97,1.89) | 1.28 (0.73,2.22) | 1.04 (0.59,1.82) | 0.98 (0.57,1.70) | Q | 0.98 (0.56,1.70) | 0.96 (0.57,1.59) | 0.95 (0.54,1.69) | 0.95 (0.58,1.54) | 0.94 (0.57,1.55) | 0.92 (0.55,1.51) | 0.81 (0.36,1.83) | 0.88 (0.53,1.45) | 0.86 (0.49,1.49) | **0.53 (0.42,0.66)** |
| 1.59 (0.87,2.91) | 1.54 (0.84,2.82) | 1.39 (0.89,2.17) | 1.31 (0.84,2.04) | 1.06 (0.68,1.67) | 1.00 (0.65,1.55) | 1.02 (0.59,1.79) | L | 0.98 (0.66,1.45) | 0.98 (0.61,1.56) | 0.97 (0.68,1.39) | 0.97 (0.67,1.40) | 0.94 (0.64,1.37) | 0.83 (0.39,1.75) | 0.90 (0.61,1.31) | 0.88 (0.56,1.36) | 0.98 (0.71,1.36) |
| 1.63 (0.93,2.86) | 1.57 (0.90,2.76) | 1.42 (0.96,2.09) | 1.34 (0.91,1.98) | 1.09 (0.73,1.62) | 1.03 (0.70,1.50) | 1.05 (0.63,1.75) | 1.02 (0.69,1.51) | M | 1.00 (0.66,1.51) | 0.99 (0.74,1.32) | 0.99 (0.73,1.33) | 0.96 (0.70,1.31) | 0.84 (0.41,1.73) | 0.92 (0.67,1.25) | 0.90 (0.61,1.32) | 1.17 (0.54,2.55) |
| 1.63 (0.88,3.02) | 1.58 (0.85,2.92) | 1.42 (0.89,2.26) | 1.34 (0.84,2.13) | 1.09 (0.68,1.74) | 1.03 (0.65,1.62) | 1.05 (0.59,1.85) | 1.02 (0.64,1.63) | 1.00 (0.66,1.51) | F | 0.99 (0.74,1.34) | 0.99 (0.67,1.46) | 0.96 (0.64,1.43) | 0.85 (0.39,1.81) | 0.92 (0.61,1.37) | 0.90 (0.56,1.42) | 0.89 (0.52,1.54) |
| 1.64 (0.95,2.81) | 1.58 (0.92,2.72) | 1.43 (1.00,2.04) | 1.35 (0.94,1.93) | 1.10 (0.76,1.58) | 1.03 (0.73,1.47) | 1.05 (0.65,1.72) | 1.03 (0.72,1.48) | 1.01 (0.75,1.34) | 1.01 (0.75,1.35) | N | 0.99 (0.77,1.29) | 0.97 (0.74,1.26) | 0.85 (0.42,1.72) | 0.92 (0.70,1.21) | 0.90 (0.63,1.29) | **0.55 (0.45,0.68)** |
| 1.65 (0.95,2.85) | 1.59 (0.92,2.76) | 1.44 (0.99,2.07) | 1.36 (0.94,1.96) | 1.10 (0.76,1.61) | 1.04 (0.73,1.49) | 1.06 (0.65,1.74) | 1.04 (0.72,1.50) | 1.01 (0.75,1.37) | 1.01 (0.68,1.50) | 1.01 (0.78,1.30) | I | 0.97 (0.73,1.29) | 0.86 (0.42,1.74) | 0.93 (0.70,1.23) | 0.91 (0.63,1.31) | **0.39 (0.29,0.54)** |
| 1.70 (0.98,2.95) | 1.64 (0.94,2.86) | **1.48 (1.02,2.15)** | 1.40 (0.96,2.03) | 1.14 (0.77,1.67) | 1.07 (0.74,1.55) | 1.09 (0.66,1.80) | 1.07 (0.73,1.55) | 1.04 (0.76,1.42) | 1.04 (0.70,1.55) | 1.04 (0.79,1.36) | 1.03 (0.78,1.37) | K | 0.88 (0.43,1.79) | 0.96 (0.79,1.15) | 0.93 (0.64,1.36) | **0.58 (0.47,0.71)** |
| 1.93 (0.82,4.52) | 1.86 (0.79,4.37) | 1.68 (0.79,3.55) | 1.59 (0.75,3.35) | 1.29 (0.61,2.74) | 1.22 (0.58,2.56) | 1.24 (0.55,2.81) | 1.21 (0.57,2.56) | 1.18 (0.58,2.43) | 1.18 (0.55,2.53) | 1.18 (0.58,2.37) | 1.17 (0.58,2.37) | 1.14 (0.56,2.31) | C | 1.09 (0.53,2.21) | 1.06 (0.50,2.24) | 0.63 (0.32,1.23) |
| **1.78 (1.02,3.09)** | 1.72 (0.99,2.99) | **1.55 (1.06,2.26)** | 1.46 (1.00,2.13) | 1.19 (0.81,1.75) | 1.12 (0.77,1.62) | 1.14 (0.69,1.89) | 1.12 (0.76,1.63) | 1.09 (0.80,1.49) | 1.09 (0.73,1.63) | 1.08 (0.83,1.42) | 1.08 (0.81,1.43) | 1.05 (0.87,1.26) | 0.92 (0.45,1.88) | J | 0.98 (0.67,1.42) | **0.53 (0.44,0.65)** |
| 1.82 (1.00,3.31) | 1.76 (0.96,3.20) | **1.58 (1.02,2.46)** | 1.49 (0.96,2.32) | 1.21 (0.78,1.90) | 1.15 (0.88,1.49) | 1.17 (0.67,2.03) | 1.14 (0.73,1.78) | 1.12 (0.76,1.64) | 1.11 (0.70,1.77) | 1.11 (0.78,1.58) | 1.10 (0.76,1.59) | 1.07 (0.74,1.56) | 0.94 (0.45,1.99) | 1.02 (0.70,1.49) | O | 0.98 (0.66,1.44) |
| **3.08 (1.84,5.16)** | **2.98 (1.78,4.99)** | **2.69 (1.96,3.68)** | **2.06 (1.49,2.85)** | **1.89 (1.34,2.67)** | **1.94 (1.41,2.65)** | **1.90 (1.51,2.39)** | 1.02 (0.73,1.41) | 0.85 (0.39,1.86) | 1.12 (0.65,1.93) | **1.82 (1.48,2.24)** | **2.54 (1.85,3.47)** | **1.74 (1.41,2.14)** | 1.60 (0.81,3.16) | **1.87 (1.54,2.27)** | 1.02 (0.69,1.51) | A |

B

| E | 0.86 (0.61,1.22) | 0.58 (0.32,1.03) | 0.55 (0.25,1.22) | **0.48 (0.24,0.96)** | 0.49 (0.22,1.08) | **0.45 (0.23,0.86)** | **0.44 (0.25,0.80)** | **0.44 (0.24,0.80)** | **0.44 (0.23,0.83)** | **0.40 (0.27,0.58)** | **0.39 (0.21,0.74)** | **0.37 (0.18,0.76)** | **0.36 (0.21,0.63)** | **0.31 (0.17,0.57)** | **0.27 (0.13,0.57)** | **0.13 (0.08,0.23)** |
| --- | --- | --- | --- | --- | --- | --- | --- | --- | --- | --- | --- | --- | --- | --- | --- | --- |
| 1.16 (0.82,1.64) | D | 0.67 (0.38,1.19) | 0.64 (0.29,1.42) | 0.56 (0.28,1.11) | 0.57 (0.25,1.26) | 0.52 (0.27,1.00) | **0.51 (0.28,0.93)** | **0.51 (0.28,0.92)** | **0.51 (0.27,0.97)** | **0.46 (0.32,0.68)** | **0.46 (0.24,0.86)** | **0.42 (0.20,0.89)** | **0.42 (0.24,0.73)** | **0.36 (0.19,0.66)** | **0.31 (0.15,0.66)** | **0.16 (0.09,0.27)** |
| 1.73 (0.97,3.08) | 1.49 (0.84,2.66) | B | 0.96 (0.48,1.93) | 0.83 (0.45,1.54) | 0.84 (0.42,1.72) | 0.78 (0.45,1.33) | 0.77 (0.44,1.35) | 0.76 (0.47,1.24) | 0.76 (0.45,1.29) | 0.69 (0.45,1.06) | 0.68 (0.41,1.14) | 0.63 (0.33,1.20) | 0.63 (0.39,1.00) | **0.53 (0.32,0.90)** | **0.47 (0.24,0.90)** | **0.23 (0.15,0.35)** |
| 1.81 (0.82,3.98) | 1.56 (0.71,3.44) | 1.04 (0.52,2.10) | H | 0.87 (0.42,1.82) | 0.88 (0.60,1.30) | 0.81 (0.42,1.57) | 0.80 (0.38,1.68) | 0.80 (0.42,1.50) | 0.79 (0.41,1.52) | 0.72 (0.36,1.44) | 0.71 (0.37,1.36) | 0.66 (0.31,1.39) | 0.65 (0.35,1.22) | 0.56 (0.29,1.08) | 0.49 (0.23,1.04) | **0.24 (0.14,0.43)** |
| **2.07 (1.05,4.12)** | 1.79 (0.90,3.56) | 1.20 (0.65,2.21) | 1.15 (0.55,2.41) | F | 1.01 (0.48,2.13) | 0.93 (0.52,1.67) | 0.92 (0.48,1.76) | 0.91 (0.62,1.35) | 0.91 (0.52,1.61) | 0.83 (0.46,1.47) | 0.82 (0.47,1.44) | 0.76 (0.39,1.49) | 0.75 (0.45,1.27) | 0.64 (0.36,1.14) | 0.56 (0.28,1.11) | **0.28 (0.17,0.45)** |
| 2.05 (0.92,4.54) | 1.77 (0.80,3.93) | 1.18 (0.58,2.40) | 1.13 (0.77,1.68) | 0.99 (0.47,2.08) | G | 0.92 (0.47,1.79) | 0.91 (0.43,1.92) | 0.90 (0.48,1.71) | 0.90 (0.47,1.74) | 0.82 (0.40,1.65) | 0.81 (0.42,1.55) | 0.75 (0.35,1.59) | 0.74 (0.39,1.39) | 0.63 (0.32,1.24) | 0.55 (0.26,1.18) | **0.27 (0.15,0.49)** |
| **2.23 (1.17,4.28)** | 1.93 (1.00,3.70) | 1.29 (0.75,2.21) | 1.24 (0.64,2.40) | 1.08 (0.60,1.93) | 1.09 (0.56,2.13) | M | 0.99 (0.55,1.79) | 0.98 (0.63,1.53) | 0.98 (0.61,1.57) | 0.89 (0.52,1.51) | 0.88 (0.66,1.18) | 0.82 (0.45,1.48) | 0.81 (0.52,1.25) | 0.69 (0.42,1.12) | 0.60 (0.33,1.11) | **0.30 (0.21,0.42)** |
| **2.25 (1.25,4.07)** | **1.94 (1.07,3.52)** | 1.30 (0.74,2.29) | 1.25 (0.60,2.62) | 1.09 (0.57,2.07) | 1.10 (0.52,2.32) | 1.01 (0.56,1.82) | C | 0.99 (0.58,1.70) | 0.99 (0.56,1.76) | 0.90 (0.57,1.42) | 0.89 (0.50,1.57) | 0.82 (0.42,1.63) | 0.82 (0.52,1.29) | 0.70 (0.41,1.19) | 0.61 (0.31,1.21) | **0.30 (0.19,0.49)** |
| **2.27 (1.26,4.10)** | **1.96 (1.08,3.55)** | 1.31 (0.80,2.14) | 1.26 (0.67,2.37) | 1.09 (0.74,1.62) | 1.11 (0.58,2.10) | 1.02 (0.65,1.59) | 1.01 (0.59,1.72) | N | 1.00 (0.65,1.53) | 0.90 (0.57,1.43) | 0.90 (0.59,1.36) | 0.83 (0.47,1.46) | 0.82 (0.57,1.19) | 0.70 (0.45,1.08) | 0.61 (0.35,1.08) | **0.30 (0.23,0.40)** |
| **2.27 (1.20,4.30)** | **1.96 (1.04,3.72)** | 1.31 (0.78,2.22) | 1.26 (0.66,2.42) | 1.10 (0.62,1.94) | 1.11 (0.58,2.14) | 1.02 (0.64,1.63) | 1.01 (0.57,1.79) | 1.00 (0.65,1.53) | I | 0.91 (0.54,1.52) | 0.90 (0.58,1.40) | 0.83 (0.47,1.49) | 0.82 (0.54,1.25) | 0.70 (0.44,1.12) | 0.62 (0.34,1.11) | **0.30 (0.22,0.42)** |
| **2.51 (1.72,3.67)** | **2.17 (1.48,3.17)** | 1.45 (0.94,2.24) | 1.39 (0.69,2.79) | 1.21 (0.68,2.15) | 1.22 (0.61,2.47) | 1.12 (0.66,1.91) | 1.11 (0.71,1.76) | 1.11 (0.70,1.75) | 1.10 (0.66,1.85) | Q | 0.99 (0.60,1.64) | 0.92 (0.49,1.73) | 0.91 (0.60,1.37) | 0.78 (0.48,1.26) | 0.68 (0.36,1.29) | **0.34 (0.22,0.50)** |
| **2.53 (1.35,4.76)** | **2.19 (1.16,4.11)** | 1.46 (0.87,2.45) | 1.40 (0.74,2.67) | 1.22 (0.70,2.14) | 1.24 (0.65,2.37) | 1.13 (0.84,1.53) | 1.12 (0.64,1.98) | 1.12 (0.74,1.69) | 1.11 (0.72,1.73) | 1.01 (0.61,1.68) | L | 0.93 (0.52,1.65) | 0.92 (0.61,1.37) | 0.78 (0.50,1.24) | 0.69 (0.38,1.23) | **0.34 (0.25,0.46)** |
| **2.73 (1.31,5.70)** | **2.36 (1.13,4.93)** | 1.58 (0.83,2.99) | 1.51 (0.72,3.19) | 1.32 (0.67,2.59) | 1.33 (0.63,2.83) | 1.22 (0.67,2.22) | 1.21 (0.61,2.40) | 1.20 (0.69,2.11) | 1.20 (0.67,2.15) | 1.09 (0.58,2.05) | 1.08 (0.61,1.91) | P | 0.99 (0.57,1.72) | 0.84 (0.47,1.53) | 0.74 (0.49,1.11) | **0.37 (0.22,0.59)** |
| **2.76 (1.59,4.81)** | **2.39 (1.37,4.16)** | 1.60 (1.00,2.54) | 1.53 (0.82,2.86) | 1.33 (0.79,2.25) | 1.35 (0.72,2.54) | 1.24 (0.80,1.91) | 1.23 (0.77,1.94) | 1.22 (0.84,1.76) | 1.21 (0.80,1.84) | 1.10 (0.73,1.66) | 1.09 (0.73,1.63) | 1.01 (0.58,1.76) | K | 0.85 (0.63,1.16) | 0.75 (0.43,1.31) | **0.37 (0.28,0.48)** |
| **3.24 (1.75,5.97)** | **2.79 (1.51,5.17)** | **1.87 (1.11,3.15)** | 1.79 (0.92,3.48) | 1.56 (0.88,2.77) | 1.58 (0.81,3.08) | 1.45 (0.89,2.36) | 1.44 (0.84,2.45) | 1.43 (0.92,2.20) | 1.42 (0.89,2.27) | 1.29 (0.79,2.10) | 1.28 (0.81,2.02) | 1.18 (0.65,2.15) | 1.17 (0.86,1.59) | J | 0.88 (0.48,1.60) | **0.43 (0.31,0.61)** |
| **3.69 (1.76,7.77)** | **3.19 (1.52,6.72)** | **2.13 (1.12,4.08)** | 2.05 (0.96,4.35) | 1.78 (0.90,3.52) | 1.80 (0.84,3.85) | 1.65 (0.90,3.03) | 1.64 (0.82,3.26) | 1.63 (0.92,2.87) | 1.62 (0.90,2.93) | 1.47 (0.77,2.80) | 1.46 (0.82,2.61) | 1.35 (0.90,2.04) | 1.34 (0.76,2.34) | 1.14 (0.63,2.08) | O | **0.49 (0.30,0.81)** |
| **7.47 (4.31,12.96)** | **6.45 (3.71,11.21)** | **4.32 (2.85,6.53)** | **4.14 (2.35,7.30)** | **3.60 (2.24,5.78)** | **3.65 (2.05,6.48)** | **3.35 (2.37,4.73)** | **3.32 (2.06,5.34)** | **3.29 (2.48,4.36)** | **3.29 (2.38,4.53)** | **2.98 (1.99,4.45)** | **2.95 (2.17,4.00)** | **2.73 (1.68,4.45)** | **2.70 (2.08,3.52)** | **2.31 (1.64,3.25)** | **2.02 (1.23,3.32)** | A |

C

| E | 0.78 (0.34,1.75) | 0.29 (0.08,1.04) | 0.30 (0.08,1.16) | 0.28 (0.08,1.04) | 0.26 (0.06,1.12) | 0.25 (0.05,1.35) | **0.25 (0.07,0.88)** | 0.24 (0.05,1.29) | **0.24 (0.06,0.95)** | 0.23 (0.05,1.08) | 0.21 (0.04,1.04) | **0.22 (0.05,0.89)** | **0.21 (0.08,0.50)** | **0.14 (0.03,0.68)** | **0.15 (0.04,0.60)** | **0.06 (0.02,0.19)** |
| --- | --- | --- | --- | --- | --- | --- | --- | --- | --- | --- | --- | --- | --- | --- | --- | --- |
| 1.29 (0.57,2.91) | D | 0.38 (0.11,1.35) | 0.38 (0.10,1.50) | 0.36 (0.10,1.34) | 0.34 (0.08,1.45) | 0.32 (0.06,1.74) | 0.32 (0.09,1.14) | 0.31 (0.06,1.66) | 0.31 (0.08,1.22) | 0.29 (0.06,1.40) | 0.27 (0.05,1.35) | 0.28 (0.07,1.15) | **0.27 (0.11,0.65)** | **0.18 (0.04,0.88)** | **0.19 (0.05,0.77)** | **0.07 (0.02,0.25)** |
| 3.42 (0.96,12.17) | 2.65 (0.74,9.46) | B | 1.01 (0.30,3.45) | 0.95 (0.35,2.58) | 0.89 (0.29,2.79) | 0.86 (0.21,3.53) | 0.86 (0.33,2.26) | 0.83 (0.20,3.37) | 0.83 (0.28,2.44) | 0.77 (0.20,2.92) | 0.72 (0.19,2.69) | 0.74 (0.25,2.18) | 0.71 (0.29,1.75) | 0.47 (0.12,1.77) | 0.51 (0.18,1.46) | **0.19 (0.08,0.44)** |
| 3.38 (0.86,13.29) | 2.62 (0.66,10.32) | 0.99 (0.29,3.37) | C | 0.94 (0.29,3.07) | 0.88 (0.24,3.28) | 0.85 (0.18,4.01) | 0.85 (0.30,2.38) | 0.82 (0.17,3.83) | 0.82 (0.25,2.65) | 0.76 (0.17,3.33) | 0.71 (0.16,3.09) | 0.73 (0.21,2.58) | 0.70 (0.25,1.99) | 0.46 (0.11,2.03) | 0.50 (0.14,1.73) | **0.19 (0.06,0.54)** |
| 3.58 (0.97,13.30) | 2.78 (0.75,10.34) | 1.05 (0.39,2.83) | 1.06 (0.33,3.45) | N | 0.94 (0.36,2.46) | 0.90 (0.25,3.22) | 0.90 (0.40,2.04) | 0.87 (0.25,3.07) | 0.87 (0.34,2.21) | 0.81 (0.32,2.06) | 0.76 (0.23,2.43) | 0.77 (0.31,1.90) | 0.74 (0.28,1.96) | 0.49 (0.15,1.60) | 0.53 (0.22,1.26) | **0.20 (0.11,0.36)** |
| 3.83 (0.89,16.48) | 2.97 (0.69,12.80) | 1.12 (0.36,3.50) | 1.13 (0.31,4.21) | 1.07 (0.41,2.81) | M | 0.96 (0.25,3.78) | 0.96 (0.36,2.55) | 0.93 (0.24,3.60) | 0.92 (0.32,2.70) | 0.86 (0.23,3.27) | 0.81 (0.23,2.87) | 0.83 (0.42,1.63) | 0.79 (0.25,2.53) | 0.52 (0.15,1.89) | 0.57 (0.21,1.54) | **0.21 (0.10,0.46)** |
| 3.97 (0.74,21.29) | 3.08 (0.57,16.54) | 1.16 (0.28,4.77) | 1.18 (0.25,5.56) | 1.11 (0.31,3.96) | 1.04 (0.26,4.07) | H | 1.00 (0.28,3.58) | 0.96 (0.44,2.12) | 0.96 (0.25,3.72) | 0.90 (0.19,4.31) | 0.84 (0.18,3.82) | 0.86 (0.23,3.21) | 0.82 (0.20,3.44) | 0.54 (0.12,2.50) | 0.59 (0.16,2.15) | **0.22 (0.07,0.68)** |
| **3.98 (1.14,13.94)** | 3.09 (0.88,10.83) | 1.16 (0.44,3.06) | 1.18 (0.42,3.30) | 1.11 (0.49,2.51) | 1.04 (0.39,2.75) | 1.00 (0.28,3.59) | K | 0.96 (0.27,3.41) | 0.96 (0.51,1.82) | 0.90 (0.27,3.04) | 0.84 (0.26,2.72) | 0.86 (0.35,2.13) | 0.82 (0.34,2.02) | 0.55 (0.17,1.78) | 0.59 (0.25,1.41) | **0.22 (0.12,0.40)** |
| 4.13 (0.78,21.97) | 3.20 (0.60,17.06) | 1.21 (0.30,4.92) | 1.22 (0.26,5.73) | 1.15 (0.33,4.08) | 1.08 (0.28,4.19) | 1.04 (0.47,2.29) | 1.04 (0.29,3.68) | G | 1.00 (0.26,3.82) | 0.93 (0.20,4.44) | 0.87 (0.19,3.93) | 0.89 (0.24,3.30) | 0.86 (0.21,3.55) | 0.57 (0.12,2.58) | 0.61 (0.17,2.21) | **0.23 (0.07,0.70)** |
| **4.14 (1.06,16.24)** | 3.21 (0.82,12.62) | 1.21 (0.41,3.58) | 1.23 (0.38,3.98) | 1.16 (0.45,2.95) | 1.08 (0.37,3.16) | 1.04 (0.27,4.04) | 1.04 (0.55,1.97) | 1.00 (0.26,3.84) | J | 0.93 (0.25,3.45) | 0.87 (0.25,3.07) | 0.89 (0.32,2.46) | 0.86 (0.30,2.44) | 0.57 (0.16,2.02) | 0.61 (0.23,1.63) | **0.23 (0.11,0.48)** |
| 4.43 (0.93,21.21) | 3.44 (0.72,16.48) | 1.30 (0.34,4.91) | 1.31 (0.30,5.73) | 1.24 (0.48,3.16) | 1.16 (0.31,4.38) | 1.11 (0.23,5.35) | 1.11 (0.33,3.77) | 1.07 (0.22,5.11) | 1.07 (0.29,3.94) | F | 0.93 (0.21,4.13) | 0.96 (0.26,3.45) | 0.92 (0.25,3.34) | 0.61 (0.14,2.71) | 0.66 (0.19,2.31) | **0.24 (0.08,0.72)** |
| 4.74 (0.96,23.51) | 3.68 (0.74,18.27) | 1.39 (0.37,5.18) | 1.40 (0.32,6.10) | 1.32 (0.41,4.27) | 1.24 (0.35,4.41) | 1.19 (0.26,5.44) | 1.19 (0.37,3.86) | 1.15 (0.25,5.18) | 1.15 (0.33,4.03) | 1.07 (0.24,4.73) | P | 1.02 (0.30,3.47) | 0.98 (0.26,3.73) | 0.65 (0.27,1.58) | 0.70 (0.21,2.32) | **0.26 (0.10,0.72)** |
| **4.64 (1.13,19.10)** | 3.60 (0.87,14.84) | 1.36 (0.46,4.01) | 1.37 (0.39,4.86) | 1.29 (0.53,3.18) | 1.21 (0.61,2.39) | 1.17 (0.31,4.37) | 1.17 (0.47,2.89) | 1.12 (0.30,4.16) | 1.12 (0.41,3.08) | 1.05 (0.29,3.78) | 0.98 (0.29,3.31) | L | 0.96 (0.32,2.90) | 0.64 (0.19,2.17) | 0.69 (0.27,1.74) | **0.26 (0.13,0.51)** |
| **4.83 (1.98,11.78)** | **3.75 (1.53,9.16)** | 1.41 (0.57,3.50) | 1.43 (0.50,4.07) | 1.35 (0.51,3.56) | 1.26 (0.39,4.03) | 1.22 (0.29,5.08) | 1.21 (0.50,2.97) | 1.17 (0.28,4.85) | 1.17 (0.41,3.32) | 1.09 (0.30,3.97) | 1.02 (0.27,3.87) | 1.04 (0.34,3.15) | Q | 0.66 (0.17,2.55) | 0.72 (0.24,2.11) | **0.27 (0.11,0.64)** |
| **7.30 (1.46,36.49)** | **5.66 (1.13,28.35)** | 2.13 (0.57,8.05) | 2.16 (0.49,9.47) | 2.04 (0.62,6.64) | 1.91 (0.53,6.86) | 1.84 (0.40,8.44) | 1.83 (0.56,6.00) | 1.77 (0.39,8.04) | 1.76 (0.50,6.26) | 1.65 (0.37,7.34) | 1.54 (0.63,3.75) | 1.57 (0.46,5.39) | 1.51 (0.39,5.81) | O | 1.08 (0.32,3.60) | **0.40 (0.14,1.12)** |
| **6.75 (1.68,27.21)** | **5.24 (1.30,21.15)** | 1.97 (0.69,5.69) | 2.00 (0.58,6.91) | 1.88 (0.79,4.47) | 1.76 (0.65,4.77) | 1.70 (0.47,6.20) | 1.70 (0.71,4.05) | 1.63 (0.45,5.89) | 1.63 (0.61,4.34) | 1.52 (0.43,5.37) | 1.42 (0.43,4.70) | 1.46 (0.57,3.70) | 1.40 (0.47,4.13) | 0.92 (0.28,3.08) | I | **0.37 (0.20,0.70)** |
| **18.13 (5.24,62.68)** | **14.05 (4.05,48.72)** | **5.30 (2.29,12.30)** | **5.37 (1.85,15.55)** | **5.06 (2.80,9.12)** | **4.73 (2.19,10.21)** | **4.56 (1.47,14.13)** | **4.56 (2.50,8.31)** | **4.39 (1.43,13.45)** | **4.38 (2.07,9.26)** | **4.09 (1.38,12.13)** | **3.82 (1.39,10.51)** | **3.91 (1.98,7.74)** | **3.75 (1.57,8.95)** | 2.48 (0.89,6.92) | **2.68 (1.42,5.08)** | A |

D

| E | 0.86 (0.70,1.05) | 0.64 (0.23,1.77) | 0.58 (0.21,1.59) | 0.53 (0.20,1.39) | 0.53 (0.29,0.95) | 0.49 (0.21,1.14) | 0.47 (0.19,1.15) | 0.45 (0.15,1.38) | 0.45 (0.17,1.24) | 0.46 (0.19,1.10) | 0.39 (0.08,1.84) | 0.38 (0.12,1.17) | 0.25 (0.09,0.71) | 0.21 (0.07,0.65) | 0.21 (0.16,0.29) | 0.14 (0.06,0.32) |
| --- | --- | --- | --- | --- | --- | --- | --- | --- | --- | --- | --- | --- | --- | --- | --- | --- |
| 1.17 (0.95,1.44) | D | 0.75 (0.27,2.07) | 0.67 (0.24,1.86) | 0.62 (0.24,1.63) | 0.62 (0.34,1.11) | 0.58 (0.25,1.33) | 0.55 (0.23,1.35) | 0.53 (0.17,1.61) | 0.53 (0.19,1.45) | 0.54 (0.23,1.28) | 0.46 (0.10,2.15) | 0.44 (0.15,1.36) | 0.29 (0.10,0.83) | 0.25 (0.08,0.76) | 0.25 (0.18,0.34) | 0.16 (0.07,0.37) |
| 1.55 (0.56,4.29) | 1.33 (0.48,3.67) | P | 0.89 (0.62,1.30) | 0.83 (0.38,1.82) | 0.82 (0.34,1.99) | 0.77 (0.40,1.49) | 0.74 (0.36,1.50) | 0.70 (0.27,1.85) | 0.70 (0.30,1.64) | 0.71 (0.36,1.40) | 0.61 (0.12,3.04) | 0.59 (0.22,1.57) | 0.39 (0.16,0.95) | 0.33 (0.12,0.88) | 0.33 (0.13,0.87) | 0.22 (0.12,0.40) |
| 1.74 (0.63,4.81) | 1.49 (0.54,4.11) | 1.12 (0.77,1.63) | O | 0.93 (0.42,2.05) | 0.92 (0.38,2.23) | 0.86 (0.44,1.67) | 0.82 (0.40,1.68) | 0.78 (0.30,2.08) | 0.79 (0.34,1.85) | 0.80 (0.40,1.58) | 0.68 (0.13,3.41) | 0.66 (0.25,1.76) | 0.44 (0.18,1.07) | 0.37 (0.14,0.99) | 0.37 (0.14,0.97) | 0.25 (0.13,0.45) |
| 1.88 (0.72,4.90) | 1.61 (0.62,4.19) | 1.21 (0.55,2.65) | 1.08 (0.49,2.38) | I | 0.99 (0.44,2.25) | 0.93 (0.52,1.65) | 0.89 (0.47,1.66) | 0.85 (0.34,2.11) | 0.85 (0.39,1.86) | 0.86 (0.48,1.56) | 0.73 (0.15,3.55) | 0.71 (0.28,1.79) | 0.47 (0.21,1.08) | 0.40 (0.16,1.00) | 0.40 (0.16,0.99) | 0.26 (0.16,0.44) |
| **1.89 (1.05,3.41)** | 1.62 (0.90,2.92) | 1.22 (0.50,2.95) | 1.09 (0.45,2.65) | 1.01 (0.44,2.29) | B | 0.94 (0.47,1.85) | 0.90 (0.43,1.89) | 0.86 (0.32,2.32) | 0.86 (0.36,2.06) | 0.87 (0.43,1.77) | 0.74 (0.16,3.40) | 0.72 (0.26,1.97) | 0.48 (0.19,1.19) | 0.40 (0.15,1.10) | 0.40 (0.25,0.66) | 0.27 (0.14,0.51) |
| 2.02 (0.88,4.67) | 1.73 (0.75,3.99) | 1.30 (0.67,2.52) | 1.16 (0.60,2.27) | 1.08 (0.61,1.91) | 1.07 (0.54,2.11) | N | 0.96 (0.60,1.52) | 0.91 (0.41,2.05) | 0.92 (0.48,1.76) | 0.93 (0.62,1.40) | 0.79 (0.18,3.48) | 0.77 (0.34,1.74) | 0.51 (0.25,1.03) | 0.43 (0.19,0.97) | 0.43 (0.20,0.93) | 0.29 (0.22,0.37) |
| 2.11 (0.87,5.15) | 1.81 (0.74,4.42) | 1.36 (0.67,2.76) | 1.22 (0.60,2.49) | 1.13 (0.60,2.11) | 1.12 (0.53,2.35) | 1.05 (0.66,1.66) | M | 0.95 (0.41,2.22) | 0.96 (0.48,1.93) | 0.97 (0.73,1.29) | 0.82 (0.18,3.85) | 0.80 (0.34,1.89) | 0.53 (0.25,1.13) | 0.45 (0.19,1.06) | 0.45 (0.19,1.04) | 0.30 (0.21,0.43) |
| 2.21 (0.73,6.74) | 1.89 (0.62,5.77) | 1.42 (0.54,3.76) | 1.27 (0.48,3.37) | 1.18 (0.47,2.94) | 1.17 (0.43,3.17) | 1.10 (0.49,2.45) | 1.05 (0.45,2.44) | G | 1.00 (0.38,2.63) | 1.02 (0.45,2.30) | 0.86 (0.16,4.62) | 0.84 (0.51,1.39) | 0.56 (0.20,1.52) | 0.47 (0.16,1.39) | 0.47 (0.16,1.37) | 0.31 (0.15,0.67) |
| 2.21 (0.81,6.03) | 1.89 (0.69,5.17) | 1.42 (0.61,3.31) | 1.27 (0.54,2.98) | 1.18 (0.54,2.57) | 1.17 (0.48,2.80) | 1.09 (0.57,2.10) | 1.04 (0.52,2.10) | 1.00 (0.38,2.61) | K | 1.01 (0.52,1.97) | 0.86 (0.17,4.30) | 0.84 (0.32,2.22) | 0.56 (0.32,0.95) | 0.47 (0.18,1.24) | 0.47 (0.18,1.23) | 0.31 (0.17,0.56) |
| 2.18 (0.91,5.19) | 1.86 (0.78,4.44) | 1.40 (0.71,2.75) | 1.25 (0.63,2.48) | 1.16 (0.64,2.10) | 1.15 (0.56,2.35) | 1.08 (0.72,1.62) | 1.03 (0.77,1.37) | 0.98 (0.43,2.23) | 0.99 (0.51,1.92) | L | 0.85 (0.18,3.91) | 0.83 (0.36,1.90) | 0.55 (0.27,1.13) | 0.46 (0.20,1.06) | 0.46 (0.21,1.04) | 0.31 (0.23,0.42) |
| 2.56 (0.54,12.11) | 2.20 (0.47,10.36) | 1.65 (0.33,8.28) | 1.48 (0.29,7.42) | 1.37 (0.28,6.63) | 1.36 (0.29,6.24) | 1.27 (0.29,5.61) | 1.21 (0.26,5.67) | 1.16 (0.22,6.20) | 1.16 (0.23,5.81) | 1.18 (0.26,5.42) | F | 0.98 (0.18,5.25) | 0.65 (0.13,3.30) | 0.54 (0.10,2.93) | 0.55 (0.12,2.50) | 0.36 (0.08,1.61) |
| 2.63 (0.86,8.05) | 2.25 (0.73,6.89) | 1.69 (0.64,4.49) | 1.51 (0.57,4.03) | 1.40 (0.56,3.51) | 1.39 (0.51,3.79) | 1.30 (0.58,2.94) | 1.24 (0.53,2.92) | 1.19 (0.72,1.96) | 1.19 (0.45,3.14) | 1.21 (0.53,2.76) | 1.02 (0.19,5.50) | H | 0.66 (0.24,1.81) | 0.56 (0.19,1.66) | 0.56 (0.19,1.64) | 0.37 (0.17,0.80) |
| **3.97 (1.40,11.27)** | **3.40 (1.20,9.65)** | **2.56 (1.05,6.23)** | 2.29 (0.93,5.60) | 2.12 (0.93,4.85) | 2.10 (0.84,5.26) | 1.97 (0.97,4.00) | 1.88 (0.89,3.98) | 1.79 (0.66,4.89) | **1.80 (1.05,3.10)** | 1.82 (0.89,3.75) | 1.55 (0.30,7.93) | 1.51 (0.55,4.15) | J | 0.84 (0.30,2.32) | 0.85 (0.31,2.29) | 0.56 (0.29,1.08) |
| **4.73 (1.53,14.57)** | **4.05 (1.31,12.46)** | **3.04 (1.14,8.13)** | **2.72 (1.01,7.30)** | **2.52 (1.00,6.37)** | 2.50 (0.91,6.85) | **2.34 (1.03,5.32)** | 2.24 (0.95,5.29) | 2.14 (0.72,6.33) | 2.14 (0.81,5.70) | 2.17 (0.94,5.00) | 1.84 (0.34,9.94) | 1.80 (0.60,5.37) | 1.19 (0.43,3.28) | C | 1.01 (0.34,2.97) | 0.67 (0.31,1.45) |
| **4.69 (3.43,6.41)** | **4.02 (2.94,5.49)** | **3.02 (1.15,7.92)** | **2.70 (1.03,7.11)** | **2.50 (1.01,6.19)** | **2.48 (1.51,4.07)** | **2.32 (1.07,5.03)** | 2.22 (0.96,5.13) | 2.12 (0.73,6.17) | 2.13 (0.82,5.54) | 2.15 (0.96,4.85) | 1.83 (0.40,8.36) | 1.79 (0.61,5.24) | 1.18 (0.44,3.20) | 0.99 (0.34,2.93) | Q | 0.66 (0.31,1.40) |
| **7.09 (3.14,16.02)** | **6.07 (2.69,13.70)** | **4.56 (2.49,8.34)** | **4.08 (2.22,7.51)** | **3.78 (2.28,6.27)** | **3.75 (1.96,7.14)** | **3.51 (2.68,4.60)** | **3.36 (2.31,4.87)** | **3.20 (1.50,6.84)** | **3.21 (1.77,5.82)** | **3.26 (2.40,4.42)** | 2.76 (0.62,12.33) | **2.70 (1.25,5.82)** | 1.78 (0.93,3.43) | 1.50 (0.69,3.26) | 1.51 (0.71,3.21) | A |

E

| L | 0.96 (0.75,1.22) | 0.96 (0.68,1.34) | 0.95 (0.68,1.33) | 0.93 (0.72,1.22) | 0.91 (0.67,1.22) | 0.86 (0.65,1.14) | 0.85 (0.65,1.11) | 0.83 (0.64,1.07) | 0.78 (0.59,1.03) | **0.71 (0.55,0.92)** | **0.54 (0.43,0.67)** |
| --- | --- | --- | --- | --- | --- | --- | --- | --- | --- | --- | --- |
| 1.04 (0.82,1.34) | K | 1.00 (0.77,1.30) | 0.99 (0.76,1.29) | 0.97 (0.81,1.17) | 0.95 (0.78,1.15) | 0.90 (0.73,1.10) | 0.88 (0.76,1.03) | 0.87 (0.74,1.02) | **0.81 (0.67,0.98)** | **0.74 (0.63,0.87)** | **0.56 (0.50,0.63)** |
| 1.04 (0.74,1.46) | 1.00 (0.77,1.30) | D | 0.99 (0.81,1.21) | 0.97 (0.73,1.30) | 0.95 (0.71,1.27) | 0.90 (0.68,1.19) | 0.88 (0.66,1.19) | 0.86 (0.67,1.11) | 0.81 (0.62,1.06) | **0.74 (0.60,0.92)** | **0.56 (0.43,0.73)** |
| 1.06 (0.75,1.48) | 1.01 (0.77,1.32) | 1.01 (0.83,1.24) | E | 0.98 (0.74,1.31) | 0.96 (0.71,1.28) | 0.91 (0.68,1.20) | 0.89 (0.66,1.20) | 0.87 (0.68,1.13) | 0.82 (0.63,1.07) | **0.75 (0.61,0.93)** | **0.57 (0.44,0.73)** |
| 1.07 (0.82,1.40) | 1.03 (0.85,1.24) | 1.03 (0.77,1.37) | 1.02 (0.76,1.36) | I | 0.97 (0.76,1.24) | 0.92 (0.78,1.09) | 0.91 (0.73,1.13) | 0.89 (0.73,1.08) | 0.83 (0.67,1.04) | **0.76 (0.63,0.93)** | **0.58 (0.49,0.67)** |
| 1.10 (0.82,1.48) | 1.06 (0.87,1.28) | 1.06 (0.79,1.42) | 1.04 (0.78,1.40) | 1.03 (0.81,1.31) | C | 0.95 (0.74,1.22) | 0.93 (0.74,1.18) | 0.91 (0.73,1.14) | 0.86 (0.67,1.09) | **0.78 (0.64,0.96)** | **0.59 (0.48,0.72)** |
| 1.16 (0.88,1.54) | 1.11 (0.91,1.36) | 1.12 (0.84,1.48) | 1.10 (0.83,1.46) | 1.09 (0.92,1.28) | 1.06 (0.82,1.36) | B | 0.99 (0.78,1.24) | 0.96 (0.79,1.18) | 0.91 (0.72,1.13) | 0.83 (0.69,1.00) | **0.62 (0.52,0.75)** |
| 1.18 (0.90,1.55) | 1.13 (0.97,1.32) | 1.13 (0.84,1.52) | 1.12 (0.83,1.51) | 1.10 (0.89,1.37) | 1.07 (0.85,1.36) | 1.02 (0.80,1.28) | J | 0.98 (0.80,1.20) | 0.92 (0.73,1.16) | 0.84 (0.69,1.03) | **0.63 (0.54,0.75)** |
| 1.21 (0.94,1.56) | 1.16 (0.98,1.36) | 1.16 (0.90,1.49) | 1.14 (0.89,1.47) | 1.13 (0.93,1.37) | 1.10 (0.88,1.37) | 1.04 (0.85,1.27) | 1.02 (0.83,1.25) | N | 0.94 (0.83,1.07) | **0.86 (0.75,0.99)** | **0.65 (0.57,0.74)** |
| 1.29 (0.97,1.70) | **1.23 (1.02,1.49)** | 1.23 (0.94,1.60) | 1.22 (0.93,1.59) | 1.20 (0.96,1.50) | 1.17 (0.92,1.48) | 1.10 (0.88,1.38) | 1.09 (0.86,1.37) | 1.06 (0.94,1.21) | F | 0.91 (0.78,1.07) | **0.69 (0.58,0.82)** |
| **1.41 (1.08,1.83)** | **1.35 (1.15,1.58)** | **1.35 (1.09,1.67)** | **1.33 (1.08,1.65)** | **1.31 (1.08,1.59)** | **1.27 (1.04,1.56)** | 1.21 (1.00,1.45) | 1.19 (0.97,1.46) | **1.16 (1.01,1.33)** | 1.09 (0.94,1.28) | Q | **0.75 (0.65,0.87)** |
| **1.86 (1.50,2.31)** | **1.78 (1.59,2.00)** | **1.78 (1.38,2.31)** | **1.77 (1.36,2.29)** | **1.74 (1.49,2.03)** | **1.69 (1.38,2.07)** | **1.60 (1.34,1.91)** | **1.58 (1.34,1.85)** | **1.54 (1.35,1.76)** | **1.45 (1.22,1.72)** | **1.33 (1.14,1.54)** | A |

F

| K | 0.91 (0.60,1.37) | 0.91 (0.54,1.52) | 0.88 (0.61,1.28) | 0.81 (0.54,1.22) | 0.80 (0.46,1.38) | 0.79 (0.46,1.37) | 0.77 (0.56,1.07) | **0.71 (0.52,0.97)** | 0.71 (0.48,1.06) | **0.59 (0.43,0.82)** | **0.35 (0.28,0.45)** |
| --- | --- | --- | --- | --- | --- | --- | --- | --- | --- | --- | --- |
| 1.10 (0.73,1.66) | C | 1.00 (0.53,1.87) | 0.97 (0.59,1.62) | 0.89 (0.53,1.50) | 0.88 (0.48,1.63) | 0.87 (0.47,1.61) | 0.85 (0.54,1.35) | 0.78 (0.47,1.30) | 0.78 (0.48,1.29) | 0.66 (0.43,1.00) | **0.39 (0.25,0.60)** |
| 1.10 (0.66,1.85) | 1.00 (0.53,1.88) | L | 0.98 (0.57,1.68) | 0.89 (0.50,1.59) | 0.88 (0.44,1.79) | 0.87 (0.43,1.77) | 0.85 (0.51,1.44) | 0.78 (0.44,1.38) | 0.79 (0.44,1.40) | 0.66 (0.38,1.14) | **0.39 (0.25,0.62)** |
| 1.13 (0.78,1.63) | 1.03 (0.62,1.70) | 1.02 (0.59,1.76) | I | 0.91 (0.64,1.30) | 0.90 (0.50,1.63) | 0.90 (0.50,1.61) | 0.88 (0.60,1.27) | 0.80 (0.52,1.24) | 0.81 (0.52,1.25) | **0.67 (0.46,0.99)** | **0.40 (0.30,0.53)** |
| 1.24 (0.82,1.87) | 1.12 (0.67,1.90) | 1.12 (0.63,2.01) | 1.10 (0.77,1.57) | B | 0.99 (0.56,1.77) | 0.98 (0.55,1.75) | 0.96 (0.64,1.44) | 0.88 (0.55,1.42) | 0.88 (0.56,1.39) | 0.74 (0.51,1.07) | **0.44 (0.30,0.63)** |
| 1.25 (0.72,2.16) | 1.13 (0.62,2.09) | 1.13 (0.56,2.29) | 1.11 (0.61,1.99) | 1.01 (0.56,1.80) | D | 0.99 (0.64,1.53) | 0.97 (0.57,1.63) | 0.89 (0.48,1.63) | 0.89 (0.51,1.54) | 0.74 (0.48,1.16) | **0.44 (0.26,0.75)** |
| 1.26 (0.73,2.18) | 1.15 (0.62,2.11) | 1.14 (0.57,2.32) | 1.12 (0.62,2.01) | 1.02 (0.57,1.82) | 1.01 (0.65,1.56) | E | 0.98 (0.58,1.65) | 0.90 (0.49,1.65) | 0.90 (0.52,1.56) | 0.75 (0.48,1.17) | **0.45 (0.26,0.76)** |
| 1.29 (0.94,1.78) | 1.17 (0.74,1.85) | 1.17 (0.69,1.98) | 1.14 (0.79,1.66) | 1.04 (0.70,1.56) | 1.03 (0.61,1.74) | 1.02 (0.61,1.73) | N | 0.92 (0.61,1.38) | 0.92 (0.70,1.21) | 0.77 (0.58,1.02) | **0.46 (0.35,0.59)** |
| **1.41 (1.03,1.93)** | 1.28 (0.77,2.12) | 1.28 (0.72,2.25) | 1.25 (0.81,1.93) | 1.14 (0.71,1.83) | 1.13 (0.61,2.08) | 1.12 (0.61,2.06) | 1.09 (0.72,1.64) | J | 1.00 (0.63,1.61) | 0.84 (0.55,1.28) | **0.50 (0.36,0.69)** |
| 1.40 (0.95,2.08) | 1.27 (0.77,2.10) | 1.27 (0.71,2.27) | 1.24 (0.80,1.93) | 1.13 (0.72,1.78) | 1.12 (0.65,1.94) | 1.11 (0.64,1.92) | 1.09 (0.83,1.43) | 1.00 (0.62,1.60) | F | 0.83 (0.60,1.15) | **0.50 (0.35,0.70)** |
| **1.68 (1.21,2.33)** | 1.53 (1.00,2.33) | 1.52 (0.88,2.64) | **1.49 (1.01,2.19)** | 1.36 (0.93,1.98) | 1.35 (0.87,2.09) | 1.33 (0.86,2.07) | 1.30 (0.98,1.72) | 1.19 (0.78,1.82) | 1.20 (0.87,1.66) | Q | **0.59 (0.44,0.80)** |
| **2.84 (2.23,3.60)** | **2.57 (1.67,3.96)** | **2.57 (1.62,4.06)** | **2.51 (1.87,3.36)** | **2.29 (1.60,3.28)** | **2.27 (1.33,3.88)** | **2.25 (1.31,3.84)** | **2.20 (1.70,2.84)** | **2.01 (1.44,2.81)** | **2.02 (1.42,2.87)** | **1.69 (1.25,2.28)** | A |

G

| E | 0.93 (0.50,1.73) | 0.90 (0.76,1.06) | 0.76 (0.52,1.12) | 0.75 (0.53,1.07) | 0.74 (0.48,1.13) | 0.74 (0.49,1.12) | 0.69 (0.44,1.10) | **0.61 (0.45,0.83)** | **0.56 (0.40,0.78)** | **0.37 (0.29,0.48)** | **0.22 (0.16,0.31)** |
| --- | --- | --- | --- | --- | --- | --- | --- | --- | --- | --- | --- |
| 1.08 (0.58,2.01) | I | 0.97 (0.52,1.81) | 0.83 (0.46,1.50) | 0.81 (0.47,1.43) | 0.80 (0.45,1.43) | 0.80 (0.44,1.45) | 0.75 (0.38,1.47) | 0.66 (0.38,1.15) | 0.60 (0.34,1.08) | **0.40 (0.23,0.71)** | **0.24 (0.14,0.41)** |
| 1.12 (0.95,1.32) | 1.03 (0.55,1.93) | D | 0.85 (0.58,1.25) | 0.84 (0.59,1.20) | 0.83 (0.54,1.27) | 0.83 (0.55,1.25) | 0.77 (0.48,1.24) | **0.68 (0.50,0.93)** | **0.62 (0.44,0.88)** | **0.42 (0.32,0.54)** | **0.25 (0.18,0.35)** |
| 1.31 (0.90,1.91) | 1.21 (0.67,2.19) | 1.17 (0.80,1.72) | C | 0.99 (0.78,1.25) | 0.97 (0.66,1.42) | 0.97 (0.70,1.35) | 0.91 (0.57,1.45) | 0.80 (0.60,1.07) | 0.73 (0.52,1.02) | **0.49 (0.37,0.64)** | **0.29 (0.22,0.39)** |
| 1.33 (0.94,1.88) | 1.23 (0.70,2.15) | 1.19 (0.83,1.69) | 1.01 (0.80,1.28) | K | 0.98 (0.71,1.36) | 0.98 (0.77,1.25) | 0.92 (0.59,1.43) | 0.81 (0.64,1.03) | **0.74 (0.56,0.99)** | **0.49 (0.39,0.62)** | **0.30 (0.24,0.36)** |
| 1.35 (0.88,2.06) | 1.25 (0.70,2.23) | 1.21 (0.79,1.85) | 1.03 (0.70,1.51) | 1.02 (0.73,1.41) | L | 1.00 (0.68,1.47) | 0.94 (0.57,1.53) | 0.82 (0.60,1.13) | 0.75 (0.53,1.08) | **0.50 (0.36,0.70)** | **0.30 (0.23,0.39)** |
| 1.35 (0.89,2.03) | 1.25 (0.69,2.26) | 1.21 (0.80,1.82) | 1.03 (0.74,1.43) | 1.02 (0.80,1.29) | 1.00 (0.68,1.46) | J | 0.93 (0.57,1.52) | 0.82 (0.60,1.13) | 0.75 (0.53,1.08) | **0.50 (0.36,0.69)** | **0.30 (0.23,0.40)** |
| 1.44 (0.91,2.30) | 1.34 (0.68,2.62) | 1.29 (0.81,2.06) | 1.10 (0.69,1.76) | 1.09 (0.70,1.69) | 1.07 (0.65,1.75) | 1.07 (0.66,1.74) | B | 0.88 (0.58,1.33) | 0.81 (0.52,1.25) | **0.54 (0.36,0.79)** | **0.32 (0.21,0.49)** |
| **1.64 (1.20,2.23)** | 1.51 (0.87,2.64) | **1.47 (1.07,2.00)** | 1.25 (0.93,1.68) | 1.23 (0.97,1.57) | 1.21 (0.88,1.66) | 1.21 (0.88,1.67) | 1.13 (0.75,1.71) | N | 0.91 (0.76,1.10) | **0.61 (0.51,0.72)** | **0.37 (0.30,0.44)** |
| **1.79 (1.28,2.50)** | 1.66 (0.93,2.96) | **1.60 (1.14,2.25)** | 1.37 (0.98,1.91) | **1.35 (1.01,1.80)** | 1.33 (0.92,1.90) | 1.33 (0.93,1.90) | 1.24 (0.80,1.92) | 1.09 (0.91,1.32) | F | **0.67 (0.54,0.83)** | **0.40 (0.31,0.52)** |
| **2.69 (2.08,3.48)** | **2.49 (1.41,4.39)** | **2.41 (1.85,3.13)** | **2.05 (1.56,2.71)** | **2.03 (1.61,2.56)** | **1.99 (1.42,2.79)** | **2.00 (1.45,2.75)** | **1.86 (1.27,2.75)** | **1.64 (1.39,1.95)** | **1.50 (1.21,1.86)** | Q | **0.60 (0.48,0.75)** |
| **4.47 (3.18,6.27)** | **4.13 (2.45,6.97)** | **4.00 (2.84,5.64)** | **3.41 (2.57,4.53)** | **3.37 (2.75,4.13)** | **3.31 (2.56,4.27)** | **3.31 (2.49,4.41)** | **3.09 (2.03,4.73)** | **2.73 (2.26,3.29)** | **2.50 (1.93,3.23)** | **1.66 (1.33,2.07)** | A |

**H**

| K | 0.83 (0.38,1.78) | 0.49 (0.23,1.04) | 0.49 (0.21,1.13) | 0.48 (0.27,0.85) | 0.44 (0.30,0.64) | 0.33 (0.10,1.10) | 0.39 (0.18,0.84) | 0.29 (0.09,0.96) | 0.27 (0.09,0.82) | 0.17 (0.08,0.35) | 0.13 (0.04,0.42) |
| --- | --- | --- | --- | --- | --- | --- | --- | --- | --- | --- | --- |
| 1.21 (0.56,2.61) | L | 0.60 (0.43,0.83) | 0.60 (0.37,0.97) | 0.58 (0.24,1.38) | 0.53 (0.23,1.25) | 0.40 (0.15,1.08) | 0.47 (0.32,0.68) | 0.35 (0.13,0.94) | 0.33 (0.14,0.78) | 0.21 (0.16,0.27) | 0.16 (0.06,0.41) |
| 2.03 (0.96,4.30) | **1.68 (1.21,2.33)** | N | 1.00 (0.63,1.59) | 0.98 (0.42,2.29) | 0.89 (0.39,2.07) | 0.68 (0.26,1.79) | 0.79 (0.66,0.94) | 0.59 (0.22,1.56) | 0.55 (0.24,1.29) | 0.35 (0.28,0.42) | 0.27 (0.11,0.68) |
| 2.03 (0.88,4.66) | **1.67 (1.03,2.73)** | 1.00 (0.63,1.58) | I | 0.97 (0.39,2.46) | 0.89 (0.36,2.22) | 0.68 (0.24,1.91) | 0.79 (0.48,1.29) | 0.59 (0.21,1.67) | 0.55 (0.22,1.39) | 0.34 (0.23,0.52) | 0.27 (0.10,0.73) |
| **2.08 (1.18,3.69)** | 1.72 (0.72,4.10) | 1.03 (0.44,2.41) | 1.03 (0.41,2.59) | J | 0.92 (0.46,1.82) | 0.69 (0.20,2.45) | 0.81 (0.34,1.93) | 0.61 (0.17,2.14) | 0.57 (0.18,1.83) | 0.35 (0.15,0.81) | 0.28 (0.08,0.94) |
| **2.27 (1.56,3.30)** | 1.88 (0.80,4.40) | 1.12 (0.48,2.58) | 1.12 (0.45,2.79) | 1.09 (0.55,2.16) | C | 0.76 (0.22,2.64) | 0.88 (0.37,2.07) | 0.66 (0.19,2.31) | 0.62 (0.19,1.97) | 0.39 (0.17,0.87) | 0.30 (0.09,1.01) |
| 3.00 (0.91,9.89) | 2.48 (0.93,6.63) | 1.48 (0.56,3.90) | 1.48 (0.52,4.17) | 1.44 (0.41,5.08) | 1.32 (0.38,4.61) | E | 1.16 (0.43,3.12) | 0.87 (0.71,1.07) | 0.82 (0.51,1.31) | 0.51 (0.20,1.32) | 0.40 (0.30,0.53) |
| **2.58 (1.19,5.57)** | **2.13 (1.47,3.09)** | **1.27 (1.06,1.52)** | 1.27 (0.78,2.08) | 1.24 (0.52,2.96) | 1.14 (0.48,2.67) | 0.86 (0.32,2.31) | F | 0.75 (0.28,2.02) | 0.70 (0.29,1.67) | 0.44 (0.33,0.57) | 0.34 (0.13,0.88) |
| **3.44 (1.04,11.36)** | **2.84 (1.06,7.62)** | 1.69 (0.64,4.48) | 1.70 (0.60,4.79) | 1.65 (0.47,5.83) | 1.51 (0.43,5.29) | 1.15 (0.94,1.40) | 1.33 (0.50,3.59) | D | 0.94 (0.58,1.51) | 0.58 (0.23,1.51) | 0.45 (0.34,0.61) |
| **3.67 (1.23,10.99)** | **3.03 (1.28,7.20)** | 1.81 (0.77,4.22) | 1.81 (0.72,4.56) | 1.76 (0.55,5.68) | 1.62 (0.51,5.15) | 1.22 (0.76,1.96) | 1.42 (0.60,3.39) | 1.07 (0.66,1.72) | B | 0.62 (0.27,1.42) | 0.49 (0.33,0.71) |
| **5.89 (2.86,12.12)** | **4.86 (3.76,6.30)** | **2.90 (2.37,3.54)** | **2.90 (1.92,4.39)** | **2.83 (1.23,6.48)** | **2.59 (1.15,5.84)** | 1.96 (0.76,5.08) | **2.28 (1.75,2.99)** | 1.71 (0.66,4.44) | 1.60 (0.70,3.66) | A | 4.50 (1.36,14.85) |
| **7.56 (2.37,24.07)** | **6.24 (2.44,16.01)** | **3.72 (1.47,9.40)** | **3.73 (1.38,10.09)** | **3.63 (1.06,12.39)** | 3.33 (0.99,11.24) | **2.52 (1.89,3.36)** | **2.93 (1.14,7.54)** | **2.20 (1.64,2.95)** | **2.06 (1.42,2.99)** | 0.22 (0.07,0.73) | Q |

**Supplementary material 3. League plots of ACR20（A）、ACR50（B）、ACR70（C）、DAS28(CRP)＜2.6（D）response rates at 12 week. League plots of ACR20（E）、ACR50（F）、ACR70（G）、DAS28(CRP)＜2.6（H）response rates at 24 week.** Bold texts indicated that the comparison between the two groups was statistically significant. ACR:American College of Rheumatology; SUCRA:surface under the cumulative ranking curve; A:Placebo; B:tofacitinib 5mg; C:baricitinib 4mg; D:upadacitinib 15mg; E:upadacitinib 30mg; F:filgotinib 200mg; G:peficitinib 100mg; H:peficitinib 150mg; I:tofacitinib 5mg+csDMARD; J:baricitinib 2mg+csDMARD; K:baricitinib 4mg+csDMARD; L:upadacitinib 15mg+csDMARD; M:upadacitinib 30mg+csDMARD; N:filgotinib 200mg+csDMARD; O:peficitinib 100mg+csDMARD; P:peficitinib 150mg+csDMARD; Q:Conventional synthetic disease-modifying antirheumatic drugs(csDMARD)

**A**

**
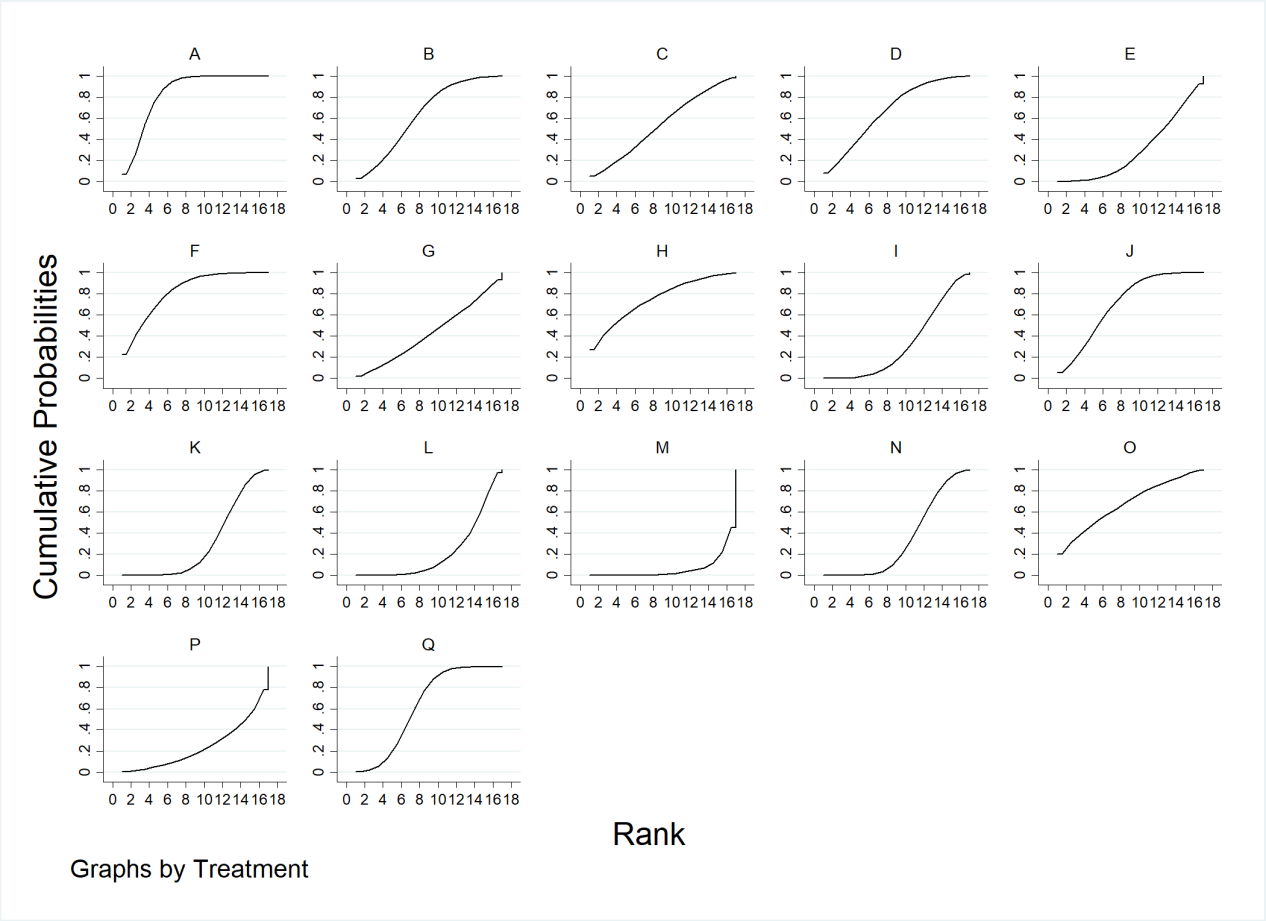
**

**B**

**
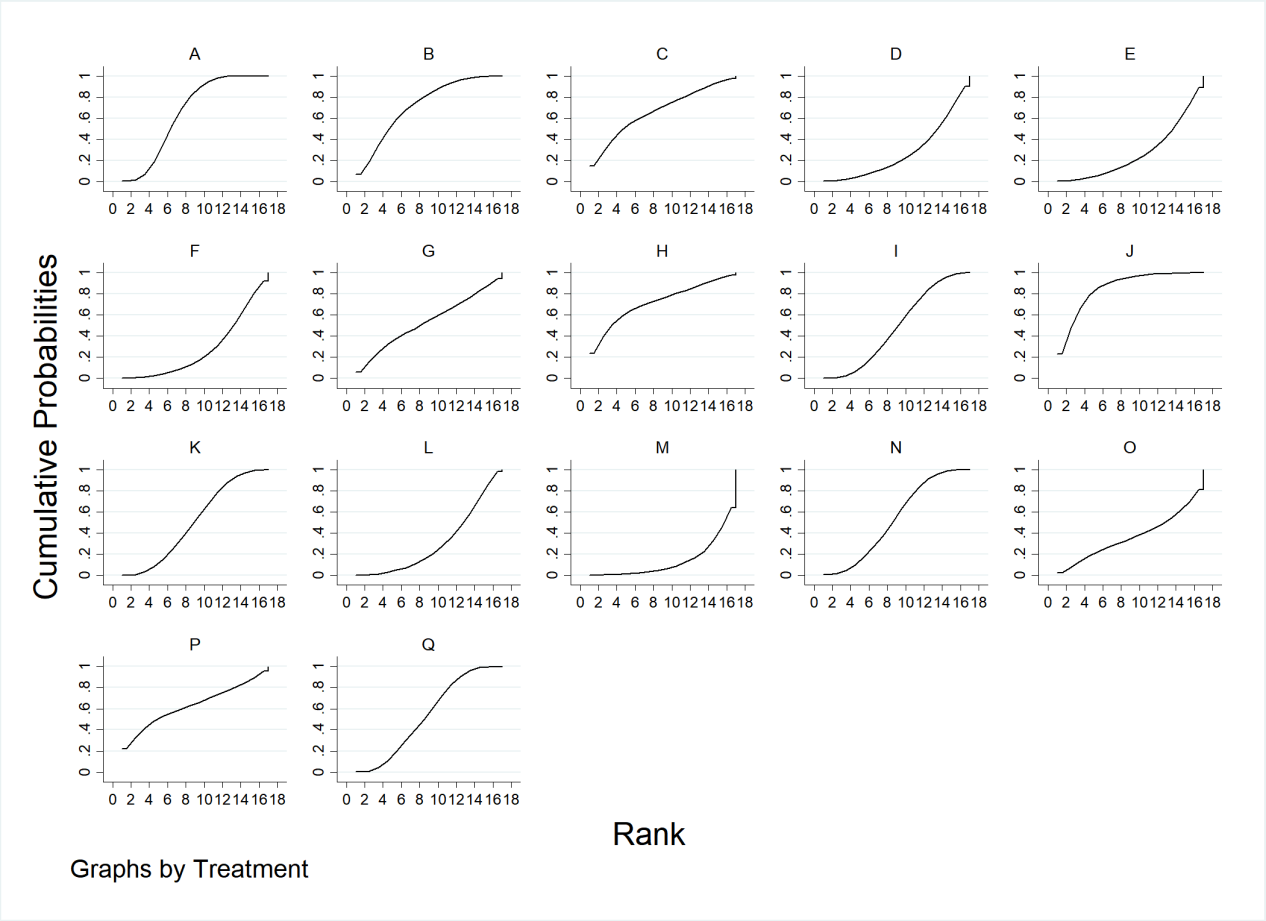
**

**C**

**
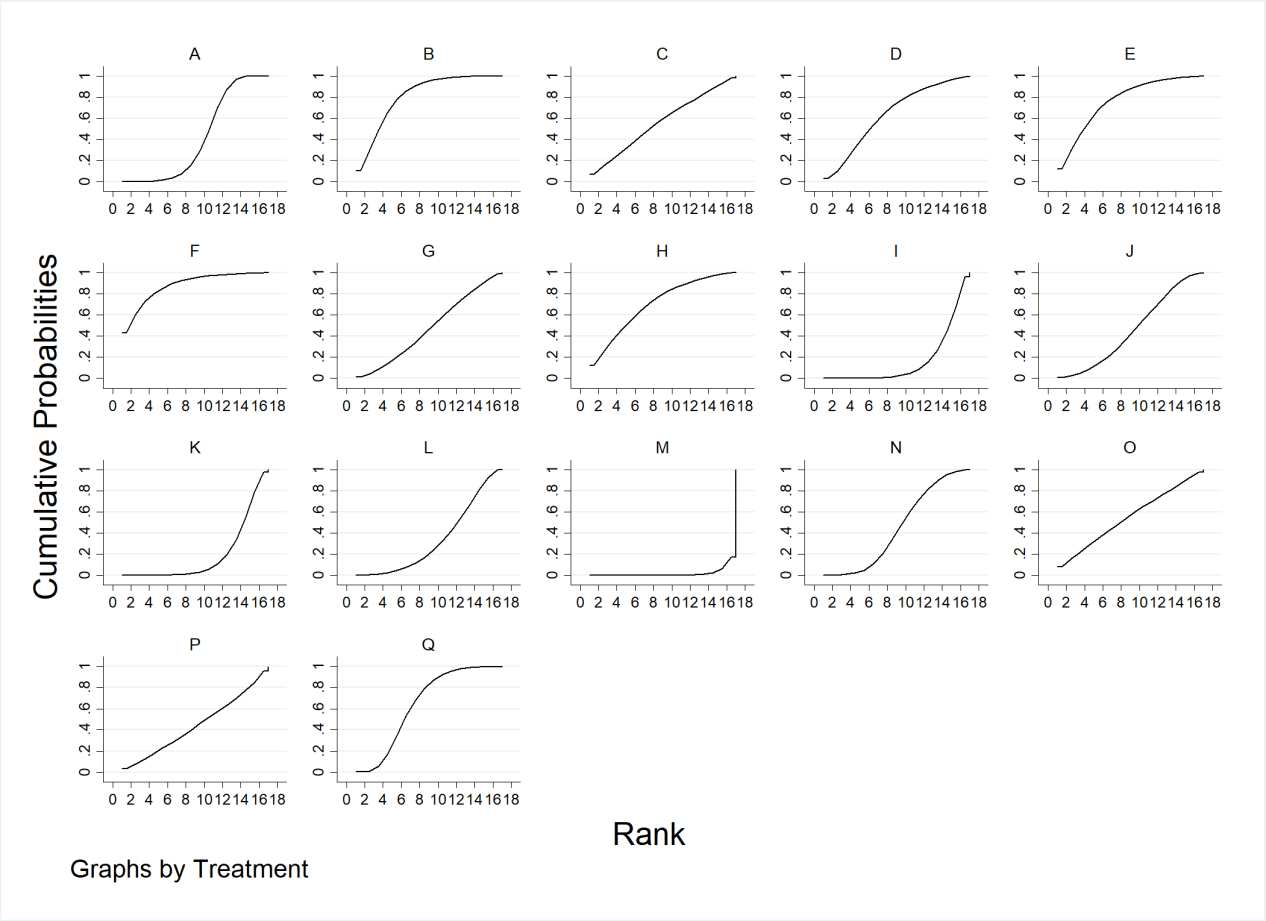
**

**D**

**
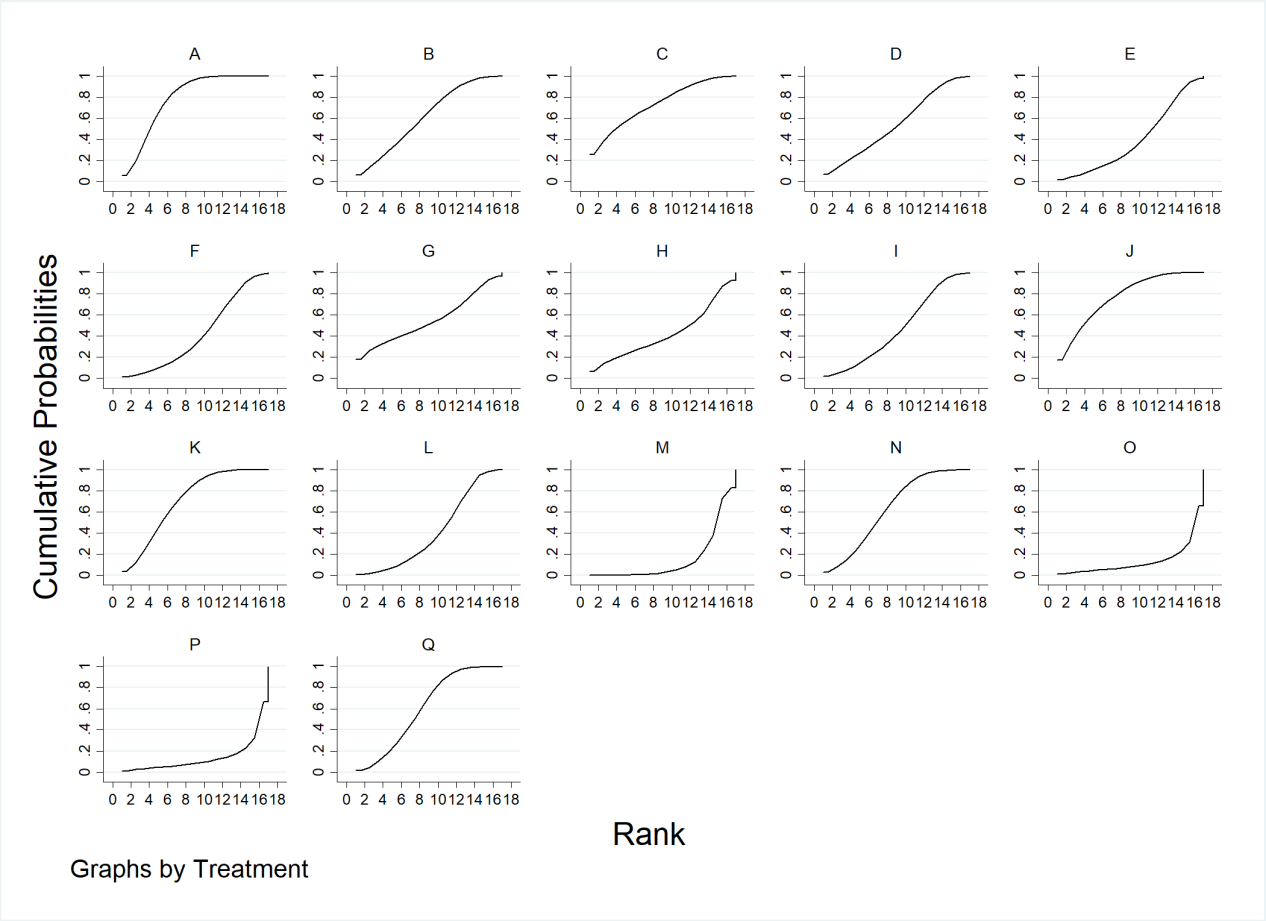
**

**E**

**
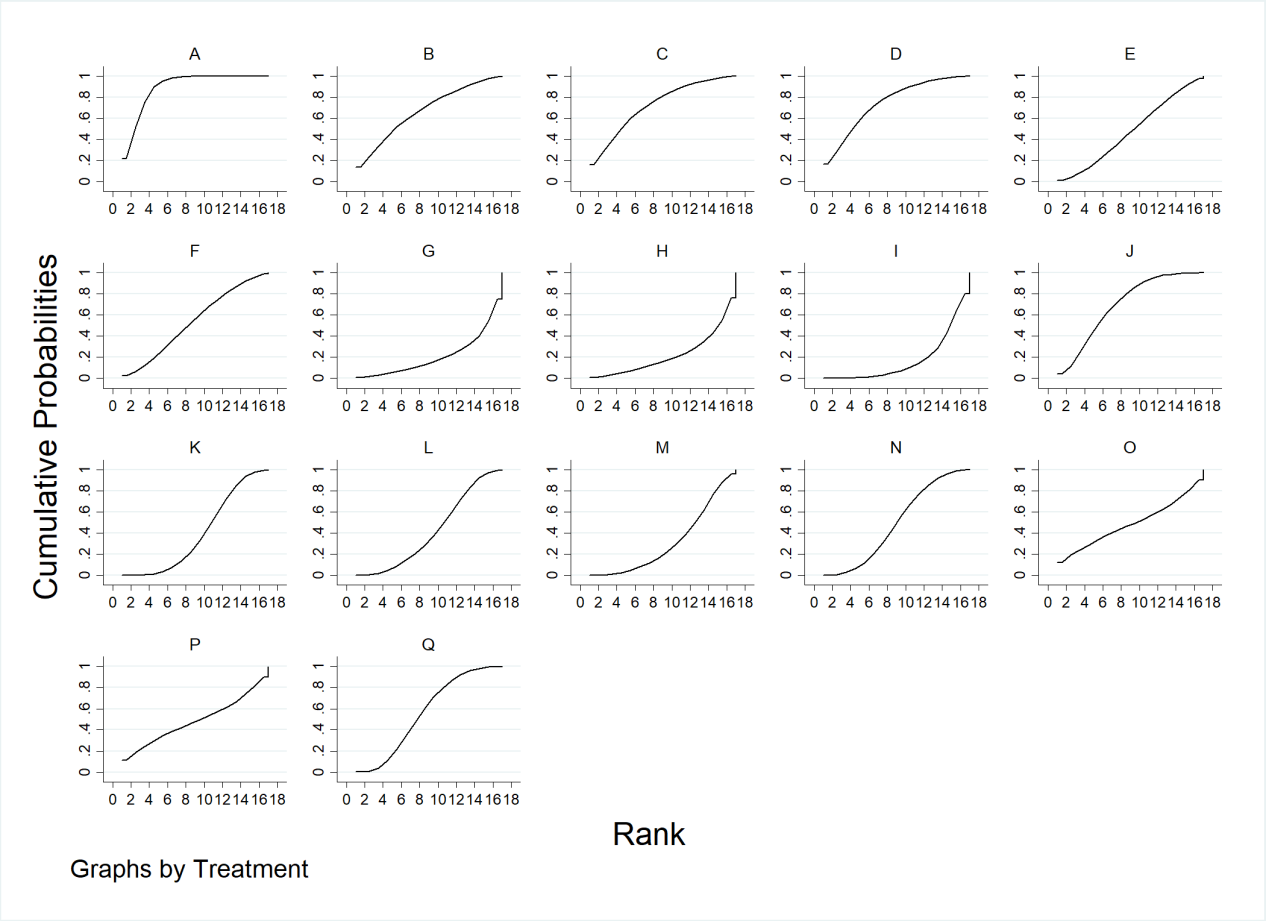
**

**F**


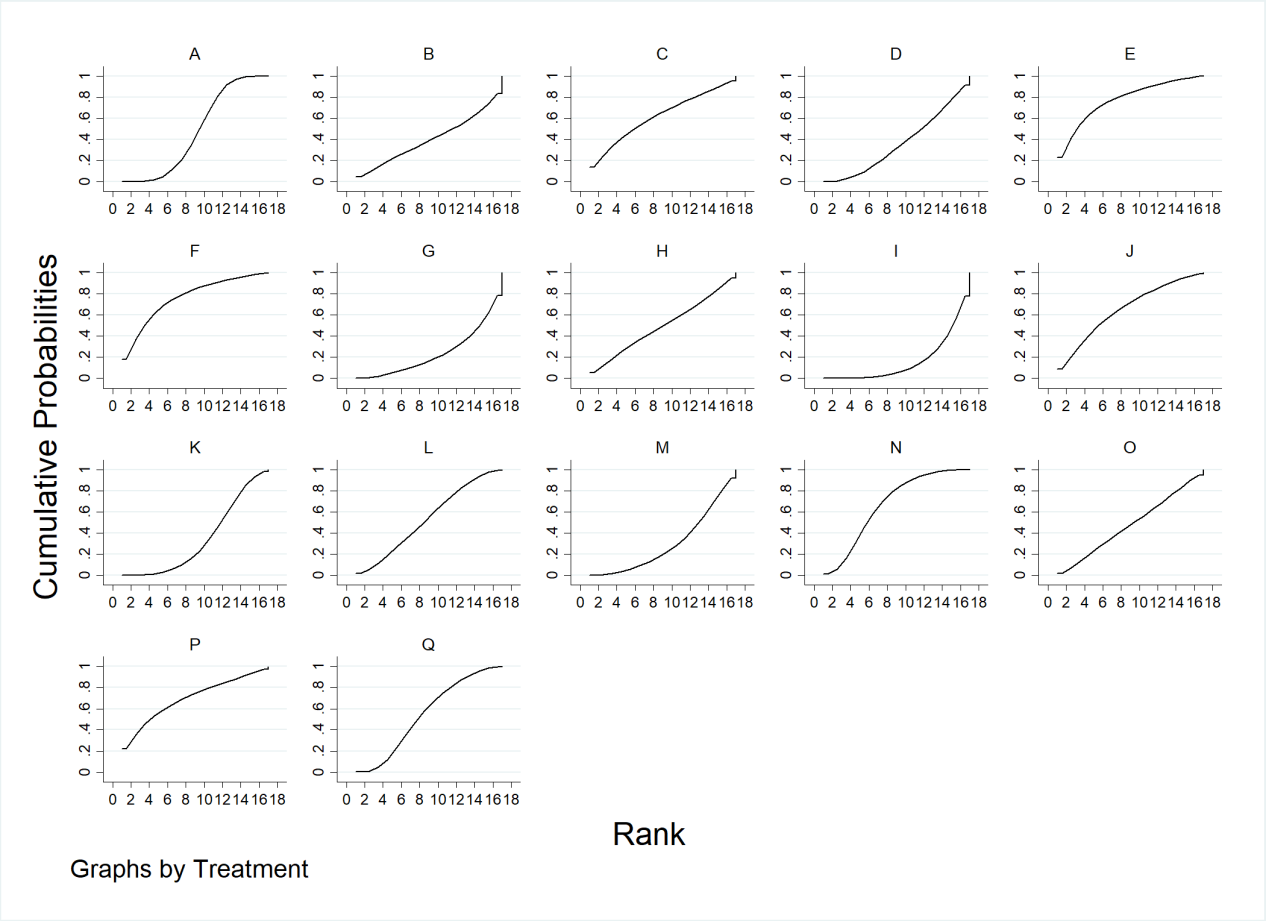


**Supplementary appendix 4. SUCRA ranking of adverse event（A）, serious adverse event（B）, adverse event leading to discontinuation of study（C）, infection（D）, serious infection（E）, malignancy（F）.** SUCRA:surface under the cumulative ranking curve; A:Placebo; B:tofacitinib 5mg; C:baricitinib 4mg; D:upadacitinib 15mg; E:upadacitinib 30mg; F:filgotinib 200mg; G:peficitinib 100mg; H:peficitinib 150mg; I:tofacitinib 5mg+csDMARD; J:baricitinib 2mg+csDMARD; K:baricitinib 4mg+csDMARD; L:upadacitinib 15mg+csDMARD; M:upadacitinib 30mg+csDMARD; N:filgotinib 200mg+csDMARD; O:peficitinib 100mg+csDMARD; P:peficitinib 150mg+csDMARD; Q:Conventional synthetic disease-modifying antirheumatic drugs(csDMARD)

**A**

| A | 0.99 (0.89,1.11) | 1.00 (0.84,1.19) | 1.03 (0.95,1.12) | 1.03 (0.86,1.23) | 1.04 (0.91,1.18) | 1.05 (0.95,1.16) | 1.05 (0.97,1.14) | 1.07 (0.93,1.24) | 1.10 (0.93,1.30) | 1.12 (1.04,1.20) | 1.13 (1.05,1.21) | 1.13 (1.07,1.20) | 1.13 (1.00,1.29) | 1.17 (0.99,1.38) | 1.16 (1.07,1.26) | 1.24 (1.12,1.38) |
| --- | --- | --- | --- | --- | --- | --- | --- | --- | --- | --- | --- | --- | --- | --- | --- | --- |
| 1.01 (0.90,1.13) | F | 1.01 (0.82,1.24) | 1.04 (0.90,1.19) | 1.03 (0.84,1.28) | 1.04 (0.90,1.21) | 1.05 (0.93,1.20) | 1.06 (0.95,1.17) | 1.08 (0.91,1.28) | 1.11 (0.91,1.35) | 1.13 (1.02,1.24) | 1.13 (0.99,1.30) | 1.14 (1.01,1.28) | 1.14 (0.99,1.31) | 1.18 (0.96,1.44) | 1.17 (1.02,1.34) | 1.25 (1.07,1.46) |
| 1.00 (0.84,1.19) | 0.99 (0.81,1.22) | H | 1.03 (0.85,1.25) | 1.03 (0.80,1.31) | 1.04 (0.83,1.28) | 1.05 (0.86,1.28) | 1.05 (0.87,1.27) | 1.07 (0.85,1.34) | 1.10 (0.93,1.29) | 1.12 (0.93,1.34) | 1.12 (0.93,1.35) | 1.13 (0.94,1.35) | 1.13 (0.91,1.40) | 1.17 (0.92,1.48) | 1.16 (0.96,1.40) | 1.24 (1.01,1.52) |
| 0.97 (0.89,1.05) | 0.96 (0.84,1.11) | 0.97 (0.80,1.18) | J | 1.00 (0.82,1.21) | 1.01 (0.87,1.17) | 1.02 (0.89,1.16) | 1.02 (0.91,1.14) | 1.04 (0.89,1.22) | 1.07 (0.89,1.28) | 1.08 (0.98,1.21) | 1.09 (0.98,1.22) | 1.10 (1.01,1.19) | 1.10 (0.95,1.28) | 1.13 (0.94,1.37) | 1.12 (1.00,1.26) | 1.20 (1.05,1.38) |
| 0.97 (0.81,1.16) | 0.97 (0.78,1.19) | 0.97 (0.76,1.25) | 1.00 (0.82,1.22) | O | 1.01 (0.81,1.26) | 1.02 (0.83,1.25) | 1.02 (0.84,1.24) | 1.04 (0.83,1.31) | 1.07 (0.84,1.36) | 1.09 (0.90,1.32) | 1.10 (0.90,1.33) | 1.10 (0.91,1.32) | 1.10 (0.89,1.37) | 1.14 (0.97,1.34) | 1.13 (0.93,1.37) | 1.21 (0.98,1.49) |
| 0.96 (0.85,1.10) | 0.96 (0.83,1.11) | 0.97 (0.78,1.20) | 0.99 (0.85,1.16) | 0.99 (0.79,1.24) | D | 1.01 (0.89,1.15) | 1.01 (0.91,1.12) | 1.03 (0.87,1.23) | 1.06 (0.86,1.31) | 1.08 (0.95,1.22) | 1.09 (0.94,1.26) | 1.09 (0.95,1.24) | 1.09 (0.99,1.21) | 1.13 (0.91,1.39) | 1.12 (0.96,1.30) | 1.20 (1.01,1.41) |
| 0.95 (0.86,1.06) | 0.95 (0.83,1.08) | 0.96 (0.78,1.17) | 0.98 (0.87,1.12) | 0.98 (0.80,1.21) | 0.99 (0.87,1.13) | B | 1.00 (0.92,1.09) | 1.02 (0.87,1.20) | 1.05 (0.87,1.27) | 1.07 (0.96,1.18) | 1.07 (0.95,1.22) | 1.08 (0.97,1.20) | 1.08 (0.95,1.23) | 1.12 (0.92,1.36) | 1.11 (0.98,1.26) | 1.19 (1.02,1.37) |
| 0.95 (0.88,1.03) | 0.95 (0.85,1.05) | 0.95 (0.79,1.15) | 0.98 (0.88,1.10) | 0.98 (0.80,1.19) | 0.99 (0.89,1.09) | 1.00 (0.92,1.08) | Q | 1.02 (0.88,1.18) | 1.05 (0.87,1.26) | 1.06 (0.99,1.14) | 1.07 (0.96,1.19) | 1.07 (0.98,1.17) | 1.08 (0.98,1.19) | 1.11 (0.92,1.34) | 1.10 (0.99,1.23) | 1.18 (1.04,1.35) |
| 0.93 (0.81,1.08) | 0.93 (0.78,1.10) | 0.94 (0.75,1.17) | 0.96 (0.82,1.13) | 0.96 (0.76,1.21) | 0.97 (0.81,1.15) | 0.98 (0.83,1.15) | 0.98 (0.85,1.13) | C | 1.03 (0.83,1.28) | 1.04 (0.90,1.21) | 1.05 (0.90,1.24) | 1.05 (0.92,1.21) | 1.06 (0.89,1.26) | 1.09 (0.87,1.36) | 1.08 (0.92,1.28) | 1.16 (0.97,1.39) |
| 0.91 (0.77,1.07) | 0.90 (0.74,1.10) | 0.91 (0.77,1.07) | 0.94 (0.78,1.13) | 0.93 (0.73,1.19) | 0.94 (0.76,1.16) | 0.95 (0.78,1.16) | 0.95 (0.79,1.15) | 0.97 (0.78,1.21) | G | 1.02 (0.85,1.21) | 1.02 (0.86,1.22) | 1.03 (0.86,1.22) | 1.03 (0.84,1.27) | 1.06 (0.84,1.34) | 1.05 (0.88,1.26) | 1.13 (0.93,1.37) |
| **0.89 (0.83,0.96)** | **0.89 (0.81,0.98)** | 0.90 (0.74,1.08) | 0.92 (0.83,1.02) | 0.92 (0.76,1.11) | 0.93 (0.82,1.05) | 0.94 (0.85,1.04) | 0.94 (0.88,1.01) | 0.96 (0.82,1.11) | 0.98 (0.82,1.18) | N | 1.01 (0.91,1.11) | 1.01 (0.93,1.10) | 1.01 (0.90,1.14) | 1.05 (0.87,1.25) | 1.04 (0.93,1.15) | 1.11 (0.98,1.26) |
| **0.89 (0.83,0.95)** | 0.88 (0.77,1.01) | 0.89 (0.74,1.07) | 0.92 (0.82,1.02) | 0.91 (0.75,1.11) | 0.92 (0.79,1.07) | 0.93 (0.82,1.05) | 0.93 (0.84,1.04) | 0.95 (0.81,1.12) | 0.98 (0.82,1.17) | 0.99 (0.90,1.10) | I | 1.00 (0.91,1.10) | 1.01 (0.87,1.16) | 1.04 (0.86,1.25) | 1.03 (0.93,1.14) | 1.10 (0.97,1.25) |
| **0.89 (0.84,0.94)** | **0.88 (0.78,0.99)** | 0.89 (0.74,1.06) | **0.91 (0.84,0.99)** | 0.91 (0.75,1.10) | 0.92 (0.80,1.05) | 0.93 (0.83,1.04) | 0.93 (0.85,1.02) | 0.95 (0.82,1.09) | 0.97 (0.82,1.16) | 0.99 (0.91,1.08) | 1.00 (0.91,1.09) | K | 1.00 (0.88,1.14) | 1.04 (0.87,1.24) | 1.03 (0.93,1.13) | 1.10 (0.98,1.24) |
| 0.88 (0.78,1.00) | 0.88 (0.76,1.01) | 0.88 (0.71,1.09) | 0.91 (0.78,1.05) | 0.91 (0.73,1.13) | 0.91 (0.83,1.01) | 0.92 (0.81,1.05) | 0.93 (0.84,1.02) | 0.94 (0.79,1.12) | 0.97 (0.79,1.19) | 0.99 (0.87,1.11) | 0.99 (0.86,1.15) | 1.00 (0.87,1.13) | E | 1.03 (0.83,1.27) | 1.02 (0.88,1.18) | 1.10 (0.93,1.29) |
| 0.86 (0.72,1.01) | 0.85 (0.69,1.04) | 0.86 (0.67,1.09) | 0.88 (0.73,1.06) | 0.88 (0.75,1.04) | 0.89 (0.72,1.10) | 0.90 (0.74,1.09) | 0.90 (0.75,1.08) | 0.92 (0.73,1.14) | 0.94 (0.74,1.19) | 0.96 (0.80,1.15) | 0.96 (0.80,1.16) | 0.97 (0.81,1.15) | 0.97 (0.79,1.20) | P | 0.99 (0.82,1.20) | 1.06 (0.87,1.30) |
| **0.86 (0.80,0.93)** | **0.86 (0.75,0.98)** | 0.86 (0.71,1.04) | 0.89 (0.79,1.00) | 0.89 (0.73,1.08) | 0.89 (0.77,1.04) | 0.90 (0.80,1.03) | 0.91 (0.81,1.01) | 0.92 (0.78,1.09) | 0.95 (0.79,1.14) | 0.96 (0.87,1.07) | 0.97 (0.87,1.08) | 0.97 (0.88,1.07) | 0.98 (0.84,1.13) | 1.01 (0.84,1.21) | L | 1.07 (0.96,1.19) |
| **0.81 (0.72,0.89)** | **0.80 (0.69,0.93)** | **0.81 (0.66,0.99)** | **0.83 (0.73,0.95)** | 0.83 (0.67,1.02) | **0.84 (0.71,0.99)** | **0.84 (0.73,0.98)** | **0.85 (0.74,0.97)** | 0.86 (0.72,1.03) | 0.89 (0.73,1.08) | 0.90 (0.79,1.02) | 0.91 (0.80,1.03) | 0.91 (0.81,1.02) | 0.91 (0.77,1.08) | 0.94 (0.77,1.15) | 0.93 (0.84,1.04) | M |

**B**

| J | 1.53 (0.81,2.90) | 0.79 (0.26,2.37) | 0.70 (0.40,1.22) | 1.45 (0.45,4.72) | 1.55 (0.71,3.38) | 0.90 (0.47,1.72) | 1.12 (0.67,1.88) | 0.89 (0.33,2.43) | 1.08 (0.39,2.98) | 1.12 (0.75,1.68) | 1.16 (0.87,1.56) | 1.14 (0.81,1.60) | 1.58 (0.73,3.44) | 0.89 (0.24,3.33) | 1.47 (0.89,2.42) | 2.03 (1.00,4.11) |
| --- | --- | --- | --- | --- | --- | --- | --- | --- | --- | --- | --- | --- | --- | --- | --- | --- |
| 0.65 (0.34,1.23) | H | 0.51 (0.14,1.83) | 0.46 (0.20,1.05) | 0.95 (0.25,3.62) | 1.01 (0.46,2.22) | 0.58 (0.29,1.17) | 0.73 (0.43,1.24) | 0.58 (0.20,1.73) | 0.71 (0.21,2.34) | 0.73 (0.43,1.25) | 0.76 (0.38,1.53) | 0.74 (0.37,1.47) | 1.03 (0.47,2.25) | 0.58 (0.13,2.51) | 0.96 (0.43,2.15) | 1.32 (0.51,3.42) |
| 1.27 (0.42,3.83) | 1.95 (0.55,6.97) | B | 0.89 (0.26,3.06) | 1.85 (0.37,9.29) | 1.98 (0.51,7.62) | 1.14 (0.32,4.10) | 1.43 (0.42,4.83) | 1.14 (0.26,5.04) | 1.38 (0.47,4.01) | 1.43 (0.44,4.62) | 1.48 (0.47,4.63) | 1.45 (0.46,4.59) | 2.01 (0.52,7.75) | 1.14 (0.20,6.33) | 1.87 (0.56,6.28) | 2.58 (0.70,9.56) |
| 1.43 (0.82,2.48) | 2.19 (0.96,5.02) | 1.12 (0.33,3.85) | C | 2.07 (0.56,7.63) | 2.22 (0.87,5.66) | 1.28 (0.55,2.96) | 1.60 (0.77,3.35) | 1.27 (0.42,3.89) | 1.55 (0.49,4.90) | 1.60 (0.82,3.14) | 1.66 (0.89,3.11) | 1.62 (0.93,2.83) | 2.26 (0.89,5.76) | 1.27 (0.31,5.32) | 2.10 (1.00,4.42) | 2.90 (1.18,7.10) |
| 0.69 (0.21,2.24) | 1.06 (0.28,4.04) | 0.54 (0.11,2.72) | 0.48 (0.13,1.78) | A | 1.07 (0.26,4.40) | 0.62 (0.16,2.38) | 0.77 (0.21,2.81) | 0.61 (0.13,2.89) | 0.75 (0.16,3.53) | 0.77 (0.22,2.69) | 0.80 (0.24,2.71) | 0.78 (0.23,2.68) | 1.09 (0.27,4.47) | 0.61 (0.19,1.95) | 1.01 (0.28,3.65) | 1.40 (0.35,5.53) |
| 0.64 (0.30,1.40) | 0.99 (0.45,2.16) | 0.51 (0.13,1.95) | 0.45 (0.18,1.15) | 0.93 (0.23,3.84) | P | 0.58 (0.27,1.21) | 0.72 (0.40,1.29) | 0.57 (0.18,1.81) | 0.70 (0.19,2.50) | 0.72 (0.35,1.49) | 0.75 (0.33,1.72) | 0.73 (0.33,1.64) | 1.02 (0.59,1.74) | 0.57 (0.12,2.65) | 0.95 (0.38,2.39) | 1.30 (0.46,3.73) |
| 1.12 (0.58,2.14) | 1.71 (0.86,3.42) | 0.88 (0.24,3.16) | 0.78 (0.34,1.81) | 1.62 (0.42,6.24) | 1.73 (0.82,3.65) | G | 1.25 (0.79,2.00) | 1.00 (0.34,2.94) | 1.21 (0.36,4.03) | 1.25 (0.68,2.31) | 1.30 (0.64,2.66) | 1.27 (0.63,2.54) | 1.76 (0.84,3.71) | 1.00 (0.23,4.33) | 1.64 (0.72,3.73) | 2.26 (0.87,5.92) |
| 0.89 (0.53,1.49) | 1.36 (0.81,2.31) | 0.70 (0.21,2.36) | 0.62 (0.30,1.30) | 1.29 (0.36,4.69) | 1.38 (0.77,2.47) | 0.80 (0.50,1.27) | Q | 0.79 (0.30,2.14) | 0.96 (0.31,3.01) | 1.00 (0.65,1.55) | 1.04 (0.57,1.88) | 1.01 (0.58,1.78) | 1.41 (0.79,2.51) | 0.79 (0.19,3.27) | 1.31 (0.64,2.69) | 1.80 (0.75,4.33) |
| 1.12 (0.41,3.06) | 1.72 (0.58,5.13) | 0.88 (0.20,3.91) | 0.78 (0.26,2.40) | 1.63 (0.35,7.66) | 1.74 (0.55,5.49) | 1.00 (0.34,2.96) | 1.26 (0.47,3.39) | N | 1.21 (0.29,5.05) | 1.26 (0.45,3.49) | 1.30 (0.46,3.71) | 1.27 (0.47,3.44) | 1.77 (0.56,5.58) | 1.00 (0.19,5.24) | 1.65 (0.54,5.06) | 2.27 (0.67,7.76) |
| 0.92 (0.34,2.55) | 1.42 (0.43,4.69) | 0.73 (0.25,2.11) | 0.65 (0.20,2.05) | 1.34 (0.28,6.36) | 1.44 (0.40,5.16) | 0.83 (0.25,2.76) | 1.04 (0.33,3.24) | 0.82 (0.20,3.43) | K | 1.04 (0.35,3.09) | 1.08 (0.37,3.09) | 1.05 (0.36,3.06) | 1.46 (0.41,5.24) | 0.82 (0.16,4.35) | 1.36 (0.44,4.21) | 1.87 (0.54,6.45) |
| 0.89 (0.60,1.33) | 1.37 (0.80,2.34) | 0.70 (0.22,2.26) | 0.62 (0.32,1.22) | 1.29 (0.37,4.50) | 1.38 (0.67,2.86) | 0.80 (0.43,1.47) | 1.00 (0.65,1.55) | 0.79 (0.29,2.20) | 0.96 (0.32,2.87) | I | 1.04 (0.63,1.71) | 1.01 (0.61,1.67) | 1.41 (0.68,2.91) | 0.79 (0.20,3.15) | 1.31 (0.69,2.49) | 1.81 (0.80,4.08) |
| 0.86 (0.64,1.15) | 1.32 (0.65,2.66) | 0.68 (0.22,2.11) | 0.60 (0.32,1.13) | 1.25 (0.37,4.21) | 1.34 (0.58,3.07) | 0.77 (0.38,1.57) | 0.97 (0.53,1.75) | 0.77 (0.27,2.18) | 0.93 (0.32,2.67) | 0.96 (0.59,1.59) | O | 0.98 (0.62,1.53) | 1.36 (0.59,3.12) | 0.77 (0.20,2.96) | 1.26 (0.71,2.26) | 1.74 (0.81,3.75) |
| 0.88 (0.62,1.24) | 1.35 (0.68,2.68) | 0.69 (0.22,2.19) | 0.62 (0.35,1.08) | 1.28 (0.37,4.37) | 1.37 (0.61,3.07) | 0.79 (0.39,1.58) | 0.99 (0.56,1.74) | 0.79 (0.29,2.12) | 0.95 (0.33,2.78) | 0.99 (0.60,1.63) | 1.02 (0.65,1.61) | L | 1.39 (0.62,3.12) | 0.79 (0.20,3.06) | 1.29 (0.70,2.37) | 1.78 (0.81,3.91) |
| 0.63 (0.29,1.38) | 0.97 (0.44,2.12) | 0.50 (0.13,1.92) | 0.44 (0.17,1.13) | 0.92 (0.22,3.77) | 0.98 (0.57,1.68) | 0.57 (0.27,1.19) | 0.71 (0.40,1.27) | 0.56 (0.18,1.78) | 0.68 (0.19,2.46) | 0.71 (0.34,1.47) | 0.74 (0.32,1.69) | 0.72 (0.32,1.61) | F | 0.56 (0.12,2.60) | 0.93 (0.37,2.34) | 1.28 (0.45,3.67) |
| 1.12 (0.30,4.18) | 1.72 (0.40,7.42) | 0.88 (0.16,4.91) | 0.78 (0.19,3.27) | 1.63 (0.51,5.17) | 1.74 (0.38,8.04) | 1.00 (0.23,4.36) | 1.26 (0.31,5.18) | 1.00 (0.19,5.24) | 1.21 (0.23,6.39) | 1.26 (0.32,4.99) | 1.30 (0.34,5.03) | 1.27 (0.33,4.97) | 1.77 (0.38,8.17) | D | 1.65 (0.40,6.74) | 2.27 (0.51,10.13) |
| 0.68 (0.41,1.12) | 1.04 (0.46,2.35) | 0.53 (0.16,1.79) | 0.48 (0.23,1.00) | 0.99 (0.27,3.56) | 1.06 (0.42,2.67) | 0.61 (0.27,1.39) | 0.76 (0.37,1.57) | 0.61 (0.20,1.86) | 0.74 (0.24,2.28) | 0.76 (0.40,1.45) | 0.79 (0.44,1.41) | 0.77 (0.42,1.42) | 1.08 (0.43,2.71) | 0.61 (0.15,2.48) | E | 1.38 (0.77,2.48) |
| **0.49 (0.24,1.00)** | 0.76 (0.29,1.96) | 0.39 (0.10,1.44) | **0.35 (0.14,0.85)** | 0.72 (0.18,2.83) | 0.77 (0.27,2.19) | 0.44 (0.17,1.16) | 0.55 (0.23,1.33) | 0.44 (0.13,1.50) | 0.53 (0.16,1.84) | 0.55 (0.25,1.25) | 0.57 (0.27,1.23) | 0.56 (0.26,1.23) | 0.78 (0.27,2.23) | 0.44 (0.10,1.96) | 0.72 (0.40,1.30) | M |

**C**

| F | 1.28 (0.52,3.16) | 1.34 (0.49,3.69) | 1.47 (0.45,4.82) | 1.65 (0.74,3.69) | 1.63 (0.61,4.39) | 1.84 (0.53,6.40) | 1.89 (0.50,7.13) | 2.09 (0.68,6.45) | 2.14 (0.76,6.04) | 2.14 (0.96,4.75) | 2.24 (0.60,8.36) | 2.28 (0.94,5.52) | 2.52 (0.92,6.91) | 3.05 (1.20,7.76) | 3.22 (1.23,8.41) | 5.40 (1.92,15.18) |
| --- | --- | --- | --- | --- | --- | --- | --- | --- | --- | --- | --- | --- | --- | --- | --- | --- |
| 0.78 (0.32,1.93) | B | 1.05 (0.49,2.23) | 1.15 (0.42,3.15) | 1.29 (0.83,2.00) | 1.27 (0.62,2.64) | 1.44 (0.50,4.18) | 1.48 (0.46,4.77) | 1.63 (0.64,4.17) | 1.68 (0.74,3.82) | 1.67 (0.93,3.00) | 1.75 (0.55,5.58) | 1.78 (0.95,3.33) | 1.97 (0.89,4.35) | 2.38 (1.20,4.73) | 2.51 (1.21,5.22) | 4.22 (1.86,9.60) |
| 0.74 (0.27,2.04) | 0.95 (0.45,2.02) | E | 1.10 (0.35,3.39) | 1.23 (0.67,2.27) | 1.21 (0.66,2.24) | 1.37 (0.43,4.43) | 1.41 (0.39,5.06) | 1.56 (0.53,4.53) | 1.60 (0.61,4.20) | 1.59 (0.75,3.38) | 1.67 (0.47,5.93) | 1.70 (0.75,3.81) | 1.88 (0.73,4.82) | 2.27 (0.97,5.31) | 2.40 (0.98,5.85) | 4.02 (1.53,10.60) |
| 0.68 (0.21,2.22) | 0.87 (0.32,2.37) | 0.91 (0.29,2.82) | H | 1.12 (0.43,2.89) | 1.11 (0.36,3.37) | 1.25 (0.36,4.31) | 1.28 (0.36,4.54) | 1.42 (0.65,3.09) | 1.45 (0.55,3.84) | 1.45 (0.59,3.58) | 1.52 (0.43,5.32) | 1.54 (0.70,3.39) | 1.71 (0.68,4.30) | 2.07 (0.86,4.96) | 2.18 (0.91,5.21) | 3.67 (1.42,9.47) |
| 0.61 (0.27,1.35) | 0.78 (0.50,1.20) | 0.81 (0.44,1.50) | 0.89 (0.35,2.31) | Q | 0.99 (0.55,1.76) | 1.12 (0.41,3.03) | 1.15 (0.37,3.52) | 1.26 (0.53,3.04) | 1.30 (0.61,2.75) | 1.30 (0.84,2.01) | 1.36 (0.45,4.12) | 1.38 (0.81,2.35) | 1.53 (0.74,3.13) | 1.85 (1.02,3.33) | 1.95 (1.02,3.73) | 3.27 (1.54,6.94) |
| 0.61 (0.23,1.65) | 0.78 (0.38,1.62) | 0.82 (0.45,1.52) | 0.90 (0.30,2.75) | 1.01 (0.57,1.81) | D | 1.13 (0.36,3.59) | 1.16 (0.33,4.10) | 1.28 (0.45,3.67) | 1.31 (0.51,3.39) | 1.31 (0.63,2.71) | 1.37 (0.39,4.81) | 1.40 (0.64,3.07) | 1.55 (0.61,3.89) | 1.87 (0.82,4.28) | 1.97 (0.83,4.71) | 3.31 (1.28,8.56) |
| 0.54 (0.16,1.88) | 0.69 (0.24,2.02) | 0.73 (0.23,2.35) | 0.80 (0.23,2.76) | 0.90 (0.33,2.43) | 0.88 (0.28,2.81) | C | 1.03 (0.26,4.06) | 1.13 (0.35,3.70) | 1.16 (0.41,3.33) | 1.16 (0.42,3.17) | 1.21 (0.31,4.76) | 1.24 (0.48,3.21) | 1.37 (0.47,3.99) | 1.65 (0.67,4.08) | 1.75 (0.63,4.87) | 2.93 (0.98,8.75) |
| 0.53 (0.14,1.99) | 0.68 (0.21,2.18) | 0.71 (0.20,2.55) | 0.78 (0.22,2.75) | 0.87 (0.28,2.68) | 0.86 (0.24,3.05) | 0.97 (0.25,3.85) | O | 1.10 (0.33,3.70) | 1.13 (0.36,3.54) | 1.13 (0.38,3.34) | 1.18 (0.46,3.02) | 1.20 (0.45,3.23) | 1.33 (0.44,4.00) | 1.61 (0.56,4.65) | 1.70 (0.59,4.89) | 2.86 (0.93,8.76) |
| 0.48 (0.15,1.48) | 0.61 (0.24,1.57) | 0.64 (0.22,1.87) | 0.71 (0.32,1.54) | 0.79 (0.33,1.90) | 0.78 (0.27,2.24) | 0.88 (0.27,2.88) | 0.91 (0.27,3.04) | G | 1.03 (0.42,2.52) | 1.02 (0.45,2.35) | 1.07 (0.32,3.56) | 1.09 (0.54,2.19) | 1.21 (0.52,2.82) | 1.46 (0.66,3.24) | 1.54 (0.70,3.40) | 2.59 (1.08,6.22) |
| 0.47 (0.17,1.32) | 0.60 (0.26,1.36) | 0.63 (0.24,1.65) | 0.69 (0.26,1.81) | 0.77 (0.36,1.63) | 0.76 (0.29,1.96) | 0.86 (0.30,2.46) | 0.88 (0.28,2.76) | 0.97 (0.40,2.40) | J | 1.00 (0.49,2.02) | 1.04 (0.34,3.23) | 1.06 (0.60,1.87) | 1.18 (0.56,2.48) | 1.42 (0.81,2.48) | 1.50 (0.76,2.96) | 2.52 (1.16,5.48) |
| 0.47 (0.21,1.04) | 0.60 (0.33,1.08) | 0.63 (0.30,1.33) | 0.69 (0.28,1.70) | 0.77 (0.50,1.20) | 0.76 (0.37,1.58) | 0.86 (0.32,2.36) | 0.89 (0.30,2.62) | 0.98 (0.43,2.24) | 1.00 (0.49,2.03) | N | 1.05 (0.36,3.06) | 1.06 (0.68,1.67) | 1.18 (0.61,2.28) | 1.43 (0.82,2.48) | 1.50 (0.84,2.69) | 2.53 (1.26,5.06) |
| 0.45 (0.12,1.67) | 0.57 (0.18,1.82) | 0.60 (0.17,2.13) | 0.66 (0.19,2.31) | 0.74 (0.24,2.24) | 0.73 (0.21,2.55) | 0.82 (0.21,3.23) | 0.85 (0.33,2.16) | 0.93 (0.28,3.10) | 0.96 (0.31,2.96) | 0.96 (0.33,2.80) | P | 1.02 (0.38,2.70) | 1.13 (0.38,3.35) | 1.36 (0.48,3.89) | 1.44 (0.51,4.09) | 2.42 (0.80,7.33) |
| 0.44 (0.18,1.07) | 0.56 (0.30,1.05) | 0.59 (0.26,1.33) | 0.65 (0.29,1.42) | 0.73 (0.43,1.23) | 0.72 (0.33,1.57) | 0.81 (0.31,2.10) | 0.83 (0.31,2.23) | 0.92 (0.46,1.84) | 0.94 (0.53,1.66) | 0.94 (0.60,1.47) | 0.98 (0.37,2.61) | A | 1.11 (0.68,1.79) | 1.34 (0.91,1.97) | 1.41 (0.97,2.05) | 2.37 (1.40,4.03) |
| 0.40 (0.14,1.09) | 0.51 (0.23,1.12) | 0.53 (0.21,1.37) | 0.58 (0.23,1.47) | 0.65 (0.32,1.34) | 0.65 (0.26,1.63) | 0.73 (0.25,2.13) | 0.75 (0.25,2.25) | 0.83 (0.35,1.94) | 0.85 (0.40,1.79) | 0.85 (0.44,1.64) | 0.89 (0.30,2.64) | 0.90 (0.56,1.46) | L | 1.21 (0.65,2.24) | 1.28 (0.69,2.35) | 2.14 (1.23,3.75) |
| **0.33 (0.13,0.83)** | **0.42 (0.21,0.83)** | 0.44 (0.19,1.03) | 0.48 (0.20,1.16) | **0.54 (0.30,0.98)** | 0.53 (0.23,1.22) | 0.60 (0.24,1.49) | 0.62 (0.22,1.79) | 0.69 (0.31,1.52) | 0.70 (0.40,1.23) | 0.70 (0.40,1.22) | 0.73 (0.26,2.10) | 0.75 (0.51,1.10) | 0.83 (0.45,1.53) | K | 1.06 (0.62,1.80) | 1.77 (0.92,3.41) |
| **0.31 (0.12,0.81)** | **0.40 (0.19,0.82)** | 0.42 (0.17,1.02) | 0.46 (0.19,1.09) | **0.51 (0.27,0.98)** | 0.51 (0.21,1.21) | 0.57 (0.21,1.60) | 0.59 (0.20,1.69) | 0.65 (0.29,1.43) | 0.67 (0.34,1.31) | 0.66 (0.37,1.19) | 0.70 (0.24,1.98) | 0.71 (0.49,1.03) | 0.78 (0.43,1.44) | 0.95 (0.55,1.62) | I | 1.68 (0.88,3.21) |
| **0.19 (0.07,0.52)** | **0.24 (0.10,0.54)** | **0.25 (0.09,0.65)** | **0.27 (0.11,0.70)** | **0.31 (0.14,0.65)** | **0.30 (0.12,0.78)** | 0.34 (0.11,1.02) | 0.35 (0.11,1.07) | **0.39 (0.16,0.93)** | **0.40 (0.18,0.86)** | **0.40 (0.20,0.79)** | 0.41 (0.14,1.26) | **0.42 (0.25,0.72)** | **0.47 (0.27,0.82)** | 0.56 (0.29,1.09) | 0.60 (0.31,1.14) | M |

**D**

| A | 1.10 (0.77,1.57) | 1.10 (0.77,1.58) | 1.14 (0.92,1.41) | 1.13 (0.78,1.65) | 1.23 (0.96,1.58) | 1.24 (0.90,1.72) | 1.28 (1.04,1.58) | 1.30 (0.70,2.41) | 1.30 (0.70,2.41) | 1.29 (0.91,1.83) | 1.34 (1.10,1.62) | 1.34 (1.15,1.57) | 1.43 (1.13,1.80) | 1.62 (0.99,2.62) | 1.63 (1.01,2.64) | 1.60 (1.23,2.08) |
| --- | --- | --- | --- | --- | --- | --- | --- | --- | --- | --- | --- | --- | --- | --- | --- | --- |
| 0.91 (0.64,1.30) | D | 1.01 (0.65,1.57) | 1.04 (0.69,1.55) | 1.03 (0.63,1.69) | 1.12 (0.87,1.45) | 1.13 (0.78,1.66) | 1.17 (0.83,1.64) | 1.19 (0.58,2.42) | 1.19 (0.58,2.42) | 1.17 (0.91,1.51) | 1.22 (0.81,1.83) | 1.22 (0.85,1.76) | 1.30 (0.85,2.00) | 1.47 (0.81,2.69) | 1.49 (0.82,2.71) | 1.46 (0.93,2.28) |
| 0.91 (0.63,1.30) | 0.99 (0.64,1.55) | C | 1.03 (0.69,1.54) | 1.03 (0.62,1.71) | 1.11 (0.78,1.60) | 1.13 (0.73,1.75) | 1.16 (0.79,1.71) | 1.18 (0.58,2.41) | 1.18 (0.58,2.41) | 1.17 (0.75,1.81) | 1.21 (0.81,1.82) | 1.22 (0.86,1.73) | 1.29 (0.84,1.99) | 1.46 (0.80,2.68) | 1.48 (0.81,2.70) | 1.45 (0.93,2.27) |
| 0.88 (0.71,1.09) | 0.96 (0.64,1.44) | 0.97 (0.65,1.45) | J | 1.00 (0.65,1.54) | 1.08 (0.79,1.48) | 1.09 (0.75,1.60) | 1.13 (0.84,1.51) | 1.15 (0.60,2.20) | 1.15 (0.60,2.20) | 1.13 (0.76,1.69) | 1.18 (0.88,1.57) | 1.18 (0.95,1.46) | 1.26 (0.91,1.73) | 1.42 (0.84,2.42) | 1.44 (0.85,2.43) | 1.41 (1.00,1.98) |
| 0.88 (0.60,1.29) | 0.97 (0.59,1.58) | 0.97 (0.59,1.62) | 1.00 (0.65,1.55) | B | 1.09 (0.71,1.65) | 1.10 (0.68,1.77) | 1.13 (0.74,1.71) | 1.15 (0.56,2.37) | 1.15 (0.56,2.37) | 1.14 (0.70,1.85) | 1.18 (0.77,1.80) | 1.19 (0.79,1.77) | 1.26 (0.81,1.97) | 1.43 (0.77,2.64) | 1.44 (0.78,2.65) | 1.41 (0.89,2.24) |
| 0.81 (0.63,1.04) | 0.89 (0.69,1.15) | 0.90 (0.62,1.29) | 0.92 (0.68,1.27) | 0.92 (0.61,1.40) | Q | 1.01 (0.77,1.33) | 1.04 (0.83,1.30) | 1.06 (0.54,2.06) | 1.06 (0.54,2.06) | 1.05 (0.82,1.34) | 1.09 (0.79,1.49) | 1.09 (0.84,1.41) | 1.16 (0.82,1.64) | 1.31 (0.76,2.27) | 1.33 (0.77,2.28) | 1.30 (0.91,1.87) |
| 0.80 (0.58,1.11) | 0.88 (0.60,1.29) | 0.89 (0.57,1.38) | 0.91 (0.62,1.34) | 0.91 (0.57,1.47) | 0.99 (0.75,1.30) | F | 1.03 (0.78,1.36) | 1.05 (0.52,2.10) | 1.05 (0.52,2.10) | 1.03 (0.71,1.50) | 1.07 (0.74,1.57) | 1.08 (0.77,1.51) | 1.15 (0.77,1.71) | 1.30 (0.72,2.33) | 1.31 (0.74,2.34) | 1.29 (0.85,1.95) |
| **0.78 (0.63,0.96)** | 0.86 (0.61,1.21) | 0.86 (0.59,1.27) | 0.89 (0.66,1.19) | 0.89 (0.58,1.34) | 0.96 (0.77,1.20) | 0.97 (0.74,1.28) | N | 1.02 (0.53,1.95) | 1.02 (0.53,1.95) | 1.01 (0.72,1.41) | 1.05 (0.78,1.39) | 1.05 (0.83,1.34) | 1.12 (0.82,1.53) | 1.26 (0.74,2.14) | 1.28 (0.76,2.15) | 1.25 (0.90,1.74) |
| 0.77 (0.41,1.42) | 0.84 (0.41,1.72) | 0.85 (0.41,1.73) | 0.87 (0.45,1.68) | 0.87 (0.42,1.79) | 0.94 (0.49,1.84) | 0.96 (0.48,1.92) | 0.98 (0.51,1.88) | O | 1.00 (0.56,1.77) | 0.99 (0.49,2.01) | 1.03 (0.54,1.96) | 1.03 (0.55,1.95) | 1.10 (0.57,2.12) | 1.24 (0.57,2.72) | 1.25 (0.57,2.74) | 1.23 (0.63,2.40) |
| 0.77 (0.41,1.42) | 0.84 (0.41,1.72) | 0.85 (0.41,1.73) | 0.87 (0.45,1.68) | 0.87 (0.42,1.79) | 0.94 (0.49,1.84) | 0.96 (0.48,1.92) | 0.98 (0.51,1.88) | 1.00 (0.56,1.77) | P | 0.99 (0.49,2.01) | 1.03 (0.54,1.96) | 1.03 (0.55,1.95) | 1.10 (0.57,2.12) | 1.24 (0.57,2.72) | 1.25 (0.57,2.74) | 1.23 (0.63,2.40) |
| 0.78 (0.55,1.10) | 0.85 (0.66,1.10) | 0.86 (0.55,1.33) | 0.88 (0.59,1.31) | 0.88 (0.54,1.43) | 0.95 (0.75,1.22) | 0.97 (0.67,1.40) | 0.99 (0.71,1.39) | 1.01 (0.50,2.06) | 1.01 (0.50,2.06) | E | 1.04 (0.70,1.55) | 1.04 (0.73,1.49) | 1.11 (0.73,1.69) | 1.25 (0.69,2.28) | 1.27 (0.70,2.30) | 1.24 (0.80,1.93) |
| **0.75 (0.62,0.91)** | 0.82 (0.55,1.23) | 0.82 (0.55,1.24) | 0.85 (0.64,1.13) | 0.85 (0.55,1.29) | 0.92 (0.67,1.26) | 0.93 (0.64,1.36) | 0.96 (0.72,1.27) | 0.97 (0.51,1.86) | 0.97 (0.51,1.86) | 0.96 (0.65,1.43) | L | 1.00 (0.78,1.29) | 1.07 (0.85,1.34) | 1.21 (0.72,2.03) | 1.22 (0.73,2.05) | 1.20 (0.86,1.66) |
| **0.74 (0.64,0.87)** | 0.82 (0.57,1.17) | 0.82 (0.58,1.17) | 0.85 (0.68,1.05) | 0.84 (0.56,1.26) | 0.92 (0.71,1.18) | 0.93 (0.66,1.30) | 0.95 (0.75,1.21) | 0.97 (0.51,1.83) | 0.97 (0.51,1.83) | 0.96 (0.67,1.37) | 1.00 (0.78,1.27) | K | 1.06 (0.80,1.41) | 1.20 (0.72,2.00) | 1.22 (0.74,2.01) | 1.19 (0.88,1.61) |
| **0.70 (0.55,0.89)** | 0.77 (0.50,1.18) | 0.77 (0.50,1.19) | 0.80 (0.58,1.10) | 0.79 (0.51,1.24) | 0.86 (0.61,1.21) | 0.87 (0.58,1.30) | 0.90 (0.65,1.23) | 0.91 (0.47,1.76) | 0.91 (0.47,1.76) | 0.90 (0.59,1.37) | 0.94 (0.75,1.18) | 0.94 (0.71,1.24) | M | 1.13 (0.66,1.94) | 1.14 (0.67,1.95) | 1.12 (0.79,1.59) |
| 0.62 (0.38,1.01) | 0.68 (0.37,1.24) | 0.68 (0.37,1.25) | 0.70 (0.41,1.20) | 0.70 (0.38,1.30) | 0.76 (0.44,1.31) | 0.77 (0.43,1.38) | 0.79 (0.47,1.34) | 0.81 (0.37,1.77) | 0.81 (0.37,1.77) | 0.80 (0.44,1.45) | 0.83 (0.49,1.39) | 0.83 (0.50,1.38) | 0.88 (0.52,1.51) | H | 1.01 (0.67,1.53) | 0.99 (0.57,1.72) |
| **0.61 (0.38,0.99)** | 0.67 (0.37,1.22) | 0.68 (0.37,1.23) | 0.70 (0.41,1.18) | 0.69 (0.38,1.28) | 0.75 (0.44,1.29) | 0.76 (0.43,1.36) | 0.78 (0.46,1.32) | 0.80 (0.37,1.74) | 0.80 (0.37,1.74) | 0.79 (0.44,1.43) | 0.82 (0.49,1.37) | 0.82 (0.50,1.36) | 0.87 (0.51,1.49) | 0.99 (0.65,1.50) | G | 0.98 (0.57,1.69) |
| **0.62 (0.48,0.81)** | 0.69 (0.44,1.07) | 0.69 (0.44,1.08) | 0.71 (0.50,1.00) | 0.71 (0.45,1.12) | 0.77 (0.53,1.10) | 0.78 (0.51,1.18) | 0.80 (0.57,1.11) | 0.81 (0.42,1.59) | 0.81 (0.42,1.59) | 0.80 (0.52,1.25) | 0.84 (0.60,1.16) | 0.84 (0.62,1.14) | 0.89 (0.63,1.27) | 1.01 (0.58,1.75) | 1.02 (0.59,1.76) | I |

**E**

| A | 0.99 (0.39,2.52) | 1.00 (0.02,48.87) | 0.74 (0.19,2.79) | 0.77 (0.10,5.72) | 0.90 (0.13,6.35) | 1.72 (0.72,4.11) | 1.61 (0.14,18.48) | 0.95 (0.08,11.42) | 1.91 (0.74,4.93) | 2.48 (0.24,25.80) | 3.39 (0.82,13.96) | 1.26 (0.02,94.80) | 1.36 (0.12,16.06) | 1.18 (0.50,2.74) | 2.41 (0.85,6.79) | 5.05 (1.10,23.05) |
| --- | --- | --- | --- | --- | --- | --- | --- | --- | --- | --- | --- | --- | --- | --- | --- | --- |
| 1.01 (0.40,2.57) | J | 1.01 (0.02,55.16) | 1.19 (0.48,2.97) | 1.74 (0.48,6.23) | 0.91 (0.11,7.93) | 0.83 (0.08,9.17) | 1.63 (0.12,22.20) | 0.96 (0.07,13.67) | 1.93 (0.51,7.30) | 2.51 (0.20,31.20) | 3.42 (0.63,18.68) | 2.43 (0.60,9.83) | 1.38 (0.10,19.26) | 5.10 (0.86,30.33) | 12.84 (0.63,262.2) | 12.84 (0.63,262.2) |
| 1.00 (0.02,48.87) | 0.99 (0.02,54.01) | C | 1.18 (0.02,62.94) | 1.72 (0.03,92.48) | 0.90 (0.01,70.04) | 0.82 (0.01,72.21) | 1.61 (0.02,159.0) | 0.95 (0.01,96.22) | 1.91 (0.03,104.4) | 2.48 (0.03,232.4) | 3.39 (0.05,212.5) | 2.41 (0.04,134.8) | 1.36 (0.01,136.4) | 5.05 (0.08,328.2) | 12.70 (0.10,1594) | 12.70 (0.10,1594) |
| 1.36 (0.36,5.15) | 0.84 (0.34,2.10) | 0.85 (0.02,45.53) | K | 1.46 (0.43,4.92) | 0.77 (0.09,6.44) | 0.70 (0.07,7.47) | 1.37 (0.10,18.13) | 0.81 (0.06,11.17) | 1.62 (0.45,5.79) | 2.11 (0.18,25.45) | 2.88 (0.55,15.00) | 2.05 (0.54,7.81) | 1.16 (0.09,15.74) | 4.29 (0.75,24.43) | 10.80 (0.54,215.0) | 10.80 (0.54,215.0) |
| 1.29 (0.17,9.56) | 0.58 (0.16,2.06) | 0.58 (0.01,31.31) | 0.68 (0.20,2.31) | N | 0.53 (0.06,4.45) | 0.48 (0.04,5.16) | 0.94 (0.07,12.51) | 0.56 (0.04,7.71) | 1.11 (0.31,4.02) | 1.44 (0.12,17.56) | 1.97 (0.65,6.02) | 1.40 (0.36,5.43) | 0.79 (0.06,10.85) | 2.94 (0.51,16.91) | 7.39 (0.37,148.1) | 7.39 (0.37,148.1) |
| 1.11 (0.16,7.77) | 1.09 (0.13,9.50) | 1.11 (0.01,85.74) | 1.30 (0.16,10.89) | 1.90 (0.22,16.07) | B | 0.91 (0.32,2.59) | 1.79 (0.08,40.50) | 1.06 (0.23,4.91) | 2.11 (0.24,18.44) | 2.75 (0.13,57.77) | 3.75 (0.34,41.70) | 2.66 (0.29,24.23) | 1.51 (0.33,6.84) | 5.58 (0.47,66.06) | 14.05 (0.44,450.8) | 14.05 (0.44,450.8) |
| 0.58 (0.24,1.39) | 1.20 (0.11,13.23) | 1.21 (0.01,106.3) | 1.43 (0.13,15.22) | 2.09 (0.19,22.44) | 1.10 (0.39,3.11) | Q | 1.96 (0.07,52.63) | 1.16 (0.37,3.58) | 2.31 (0.21,25.65) | 3.01 (0.12,75.38) | 4.11 (0.30,56.76) | 2.92 (0.25,33.58) | 1.66 (0.55,4.94) | 6.12 (0.42,89.48) | 15.42 (0.41,576.4) | 15.42 (0.41,576.4) |
| 0.62 (0.05,7.09) | 0.61 (0.05,8.34) | 0.62 (0.01,61.00) | 0.73 (0.06,9.61) | 1.06 (0.08,14.17) | 0.56 (0.02,12.69) | 0.51 (0.02,13.70) | G | 0.59 (0.02,19.16) | 1.18 (0.09,16.16) | 1.54 (0.26,9.13) | 2.10 (0.13,35.17) | 1.49 (0.11,21.09) | 0.84 (0.03,27.08) | 3.12 (0.18,55.23) | 7.87 (0.18,339.4) | 7.87 (0.18,339.4) |
| 1.05 (0.09,12.54) | 1.04 (0.07,14.71) | 1.05 (0.01,105.7) | 1.23 (0.09,16.97) | 1.80 (0.13,25.01) | 0.95 (0.20,4.41) | 0.86 (0.28,2.67) | 1.69 (0.05,54.89) | D | 2.00 (0.14,28.50) | 2.60 (0.09,78.91) | 3.55 (0.20,61.87) | 2.52 (0.17,37.19) | 1.43 (0.51,4.01) | 5.29 (0.29,97.09) | 13.32 (0.30,591.3) | 13.32 (0.30,591.3) |
| 0.52 (0.20,1.36) | 0.52 (0.14,1.97) | 0.52 (0.01,28.73) | 0.62 (0.17,2.20) | 0.90 (0.25,3.27) | 0.47 (0.05,4.14) | 0.43 (0.04,4.79) | 0.85 (0.06,11.59) | 0.50 (0.04,7.13) | I | 1.30 (0.10,16.28) | 1.78 (0.32,9.78) | 1.26 (0.31,5.15) | 0.72 (0.05,10.05) | 2.65 (0.44,15.87) | 6.66 (0.32,136.7) | 6.66 (0.32,136.7) |
| 0.40 (0.04,4.19) | 0.40 (0.03,4.96) | 0.40 (0.00,37.72) | 0.47 (0.04,5.71) | 0.69 (0.06,8.42) | 0.36 (0.02,7.66) | 0.33 (0.01,8.30) | 0.65 (0.11,3.86) | 0.38 (0.01,11.65) | 0.77 (0.06,9.61) | H | 1.36 (0.09,21.06) | 0.97 (0.07,12.55) | 0.55 (0.02,16.47) | 2.03 (0.12,33.12) | 5.12 (0.13,207.5) | 5.12 (0.13,207.5) |
| 0.30 (0.07,1.22) | 0.29 (0.05,1.59) | 0.30 (0.00,18.52) | 0.35 (0.07,1.81) | 0.51 (0.17,1.55) | 0.27 (0.02,2.97) | 0.24 (0.02,3.36) | 0.48 (0.03,7.99) | 0.28 (0.02,4.91) | 0.56 (0.10,3.10) | 0.73 (0.05,11.31) | F | 0.71 (0.12,4.11) | 0.40 (0.02,6.92) | 1.49 (0.19,11.88) | 3.75 (0.15,91.93) | 3.75 (0.15,91.93) |
| 0.79 (0.01,59.90) | 0.41 (0.10,1.66) | 0.42 (0.01,23.26) | 0.49 (0.13,1.86) | 0.71 (0.18,2.77) | 0.38 (0.04,3.42) | 0.34 (0.03,3.93) | 0.67 (0.05,9.49) | 0.40 (0.03,5.84) | 0.79 (0.19,3.23) | 1.03 (0.08,13.35) | 1.41 (0.24,8.14) | L | 0.57 (0.04,8.23) | 2.10 (0.33,13.19) | 5.28 (0.25,111.4) | 5.28 (0.25,111.4) |
| 0.73 (0.06,8.63) | 0.73 (0.05,10.13) | 0.73 (0.01,73.29) | 0.86 (0.06,11.69) | 1.26 (0.09,17.22) | 0.66 (0.15,3.00) | 0.60 (0.20,1.80) | 1.18 (0.04,37.94) | 0.70 (0.25,1.96) | 1.40 (0.10,19.63) | 1.82 (0.06,54.53) | 2.48 (0.14,42.66) | 1.76 (0.12,25.62) | E | 3.70 (0.20,66.95) | 9.31 (0.21,409.1) | 9.31 (0.21,409.1) |
| 0.85 (0.36,1.98) | 0.20 (0.03,1.17) | 0.20 (0.00,12.90) | 0.23 (0.04,1.33) | 0.34 (0.06,1.96) | 0.18 (0.02,2.12) | 0.16 (0.01,2.39) | 0.32 (0.02,5.66) | 0.19 (0.01,3.47) | 0.38 (0.06,2.27) | 0.49 (0.03,8.02) | 0.67 (0.08,5.36) | 0.48 (0.08,3.00) | 0.27 (0.01,4.90) | M | 2.52 (0.10,64.68) | 2.52 (0.10,64.68) |
| 0.42 (0.15,1.17) | 0.08 (0.00,1.59) | 0.08 (0.00,9.88) | 0.09 (0.00,1.84) | 0.14 (0.01,2.71) | 0.07 (0.00,2.28) | 0.06 (0.00,2.42) | 0.13 (0.00,5.48) | 0.08 (0.00,3.34) | 0.15 (0.01,3.08) | 0.20 (0.00,7.93) | 0.27 (0.01,6.54) | 0.19 (0.01,4.00) | 0.11 (0.00,4.72) | 0.40 (0.02,10.20) | O | 1.00 (0.34,2.91) |
| **0.20 (0.04,0.91)** | 0.08 (0.00,1.59) | 0.08 (0.00,9.88) | 0.09 (0.00,1.84) | 0.14 (0.01,2.71) | 0.07 (0.00,2.28) | 0.06 (0.00,2.42) | 0.13 (0.00,5.48) | 0.08 (0.00,3.34) | 0.15 (0.01,3.08) | 0.20 (0.00,7.93) | 0.27 (0.01,6.54) | 0.19 (0.01,4.00) | 0.11 (0.00,4.72) | 0.40 (0.02,10.20) | 1.00 (0.34,2.91) | P |

**F**

| E | 1.08 (0.05,23.78) | 1.38 (0.02,105.0) | 2.04 (0.16,25.91) | 1.99 (0.05,76.38) | 1.99 (0.03,119.5) | 2.99 (0.31,28.68) | 3.49 (0.12,103.2) | 3.76 (0.07,211.0) | 4.13 (0.10,166.5) | 4.23 (0.23,79.22) | 6.40 (0.08,487.7) | 5.93 (0.71,49.19) | 6.45 (0.32,129.9) | 7.91 (0.26,239.0) | 11.71 (0.27,502.0) | 14.76 (0.56,391.4) |
| --- | --- | --- | --- | --- | --- | --- | --- | --- | --- | --- | --- | --- | --- | --- | --- | --- |
| 0.92 (0.04,20.34) | F | 1.27 (0.02,83.26) | 1.88 (0.20,17.37) | 1.84 (0.06,59.39) | 1.84 (0.04,96.20) | 2.77 (0.34,22.80) | 3.23 (0.13,78.19) | 3.48 (0.07,165.2) | 3.82 (0.11,128.3) | 3.91 (0.26,57.99) | 5.92 (0.09,386.4) | 5.48 (0.44,68.30) | 5.97 (0.37,97.51) | 7.31 (0.30,181.2) | 10.83 (0.30,388.0) | 13.65 (0.63,294.2) |
| 0.73 (0.01,55.34) | 0.78 (0.01,51.27) | P | 1.48 (0.04,54.31) | 1.45 (0.03,76.75) | 1.45 (0.02,132.9) | 2.17 (0.05,87.71) | 2.53 (0.07,94.38) | 2.73 (0.04,186.3) | 3.00 (0.12,73.14) | 3.07 (0.13,74.84) | 4.64 (0.05,424.7) | 4.30 (0.08,223.1) | 4.68 (0.15,143.6) | 5.74 (0.15,218.1) | 8.50 (0.16,449.0) | 10.71 (0.32,360.2) |
| 0.49 (0.04,6.25) | 0.53 (0.06,4.90) | 0.68 (0.02,24.88) | N | 0.98 (0.06,15.86) | 0.98 (0.03,29.41) | 1.47 (0.46,4.73) | 1.71 (0.16,18.59) | 1.85 (0.07,46.66) | 2.03 (0.12,33.57) | 2.08 (0.39,11.05) | 3.14 (0.09,115.4) | 2.91 (0.48,17.81) | 3.17 (0.48,20.84) | 3.89 (0.35,43.43) | 5.75 (0.32,103.1) | 7.25 (0.78,67.11) |
| 0.50 (0.01,19.26) | 0.54 (0.02,17.51) | 0.69 (0.01,36.76) | 1.02 (0.06,16.58) | J | 1.00 (0.02,40.30) | 1.50 (0.09,26.31) | 1.75 (0.10,32.19) | 1.89 (0.05,71.62) | 2.08 (0.08,54.34) | 2.12 (0.20,22.56) | 3.21 (0.06,170.6) | 2.98 (0.12,71.55) | 3.24 (0.37,28.22) | 3.97 (0.21,74.78) | 5.88 (0.21,165.0) | 7.41 (0.46,119.7) |
| 0.50 (0.01,30.07) | 0.54 (0.01,28.28) | 0.69 (0.01,63.47) | 1.02 (0.03,30.65) | 1.00 (0.02,40.17) | C | 1.50 (0.05,45.58) | 1.75 (0.05,65.52) | 1.89 (0.03,129.2) | 2.07 (0.04,103.7) | 2.12 (0.09,51.99) | 3.21 (0.03,294.6) | 2.97 (0.07,118.2) | 3.24 (0.16,65.34) | 3.97 (0.10,151.4) | 5.87 (0.11,311.5) | 7.40 (0.22,250.1) |
| 0.33 (0.03,3.20) | 0.36 (0.04,2.98) | 0.46 (0.01,18.59) | 0.68 (0.21,2.19) | 0.67 (0.04,11.64) | 0.67 (0.02,20.23) | Q | 1.17 (0.09,14.54) | 1.26 (0.04,35.23) | 1.38 (0.07,25.73) | 1.41 (0.22,9.12) | 2.14 (0.05,86.27) | 1.98 (0.50,7.91) | 2.16 (0.30,15.58) | 2.64 (0.21,33.91) | 3.91 (0.19,78.79) | 4.93 (0.46,52.97) |
| 0.29 (0.01,8.48) | 0.31 (0.01,7.50) | 0.39 (0.01,14.71) | 0.58 (0.05,6.33) | 0.57 (0.03,10.48) | 0.57 (0.02,21.39) | 0.86 (0.07,10.69) | L | 1.08 (0.04,27.63) | 1.18 (0.07,19.92) | 1.21 (0.22,6.63) | 1.83 (0.05,68.27) | 1.70 (0.10,30.20) | 1.85 (0.23,15.11) | 2.27 (0.51,10.13) | 3.36 (0.18,61.17) | 4.23 (0.45,40.00) |
| 0.27 (0.00,14.90) | 0.29 (0.01,13.64) | 0.37 (0.01,24.97) | 0.54 (0.02,13.65) | 0.53 (0.01,20.05) | 0.53 (0.01,36.29) | 0.80 (0.03,22.29) | 0.93 (0.04,23.77) | H | 1.10 (0.03,38.81) | 1.12 (0.07,17.80) | 1.70 (0.02,115.9) | 1.57 (0.04,58.16) | 1.71 (0.08,35.32) | 2.10 (0.08,55.07) | 3.11 (0.30,32.76) | 3.92 (0.17,89.62) |
| 0.24 (0.01,9.75) | 0.26 (0.01,8.78) | 0.33 (0.01,8.13) | 0.49 (0.03,8.14) | 0.48 (0.02,12.61) | 0.48 (0.01,24.14) | 0.72 (0.04,13.49) | 0.84 (0.05,14.20) | 0.91 (0.03,32.17) | O | 1.02 (0.11,9.74) | 1.55 (0.03,77.09) | 1.43 (0.06,36.46) | 1.56 (0.12,20.37) | 1.91 (0.11,33.02) | 2.83 (0.11,73.67) | 3.57 (0.24,52.61) |
| 0.24 (0.01,4.43) | 0.26 (0.02,3.79) | 0.33 (0.01,7.94) | 0.48 (0.09,2.56) | 0.47 (0.04,5.00) | 0.47 (0.02,11.55) | 0.71 (0.11,4.57) | 0.83 (0.15,4.52) | 0.89 (0.06,14.09) | 0.98 (0.10,9.30) | A | 1.51 (0.06,36.85) | 1.40 (0.14,14.29) | 1.52 (0.44,5.24) | 1.87 (0.33,10.67) | 2.77 (0.26,29.12) | 3.49 (0.80,15.17) |
| 0.16 (0.00,11.91) | 0.17 (0.00,11.04) | 0.22 (0.00,19.71) | 0.32 (0.01,11.69) | 0.31 (0.01,16.53) | 0.31 (0.00,28.62) | 0.47 (0.01,18.88) | 0.55 (0.01,20.32) | 0.59 (0.01,40.11) | 0.65 (0.01,32.19) | 0.66 (0.03,16.11) | B | 0.93 (0.02,48.04) | 1.01 (0.03,30.93) | 1.24 (0.03,46.97) | 1.83 (0.03,96.68) | 2.31 (0.07,77.57) |
| 0.17 (0.02,1.40) | 0.18 (0.01,2.27) | 0.23 (0.00,12.06) | 0.34 (0.06,2.10) | 0.34 (0.01,8.08) | 0.34 (0.01,13.39) | 0.51 (0.13,2.02) | 0.59 (0.03,10.47) | 0.63 (0.02,23.45) | 0.70 (0.03,17.74) | 0.71 (0.07,7.28) | 1.08 (0.02,55.98) | D | 1.09 (0.10,12.17) | 1.33 (0.07,24.33) | 1.98 (0.07,53.92) | 2.49 (0.16,38.90) |
| 0.16 (0.01,3.12) | 0.17 (0.01,2.74) | 0.21 (0.01,6.55) | 0.32 (0.05,2.08) | 0.31 (0.04,2.69) | 0.31 (0.02,6.24) | 0.46 (0.06,3.35) | 0.54 (0.07,4.42) | 0.58 (0.03,12.02) | 0.64 (0.05,8.37) | 0.66 (0.19,2.25) | 0.99 (0.03,30.42) | 0.92 (0.08,10.27) | K | 1.23 (0.14,10.37) | 1.82 (0.13,25.88) | 2.29 (0.34,15.59) |
| 0.13 (0.00,3.82) | 0.14 (0.01,3.39) | 0.17 (0.00,6.62) | 0.26 (0.02,2.88) | 0.25 (0.01,4.74) | 0.25 (0.01,9.63) | 0.38 (0.03,4.86) | 0.44 (0.10,1.97) | 0.48 (0.02,12.47) | 0.52 (0.03,9.02) | 0.53 (0.09,3.05) | 0.81 (0.02,30.73) | 0.75 (0.04,13.66) | 0.82 (0.10,6.90) | M | 1.48 (0.08,27.67) | 1.87 (0.19,18.23) |
| 0.09 (0.00,3.66) | 0.09 (0.00,3.31) | 0.12 (0.00,6.21) | 0.17 (0.01,3.12) | 0.17 (0.01,4.77) | 0.17 (0.00,9.03) | 0.26 (0.01,5.15) | 0.30 (0.02,5.43) | 0.32 (0.03,3.38) | 0.35 (0.01,9.18) | 0.36 (0.03,3.80) | 0.55 (0.01,28.85) | 0.51 (0.02,13.81) | 0.55 (0.04,7.85) | 0.68 (0.04,12.62) | G | 1.26 (0.08,20.20) |
| 0.07 (0.00,1.80) | 0.07 (0.00,1.58) | 0.09 (0.00,3.14) | 0.14 (0.01,1.28) | 0.13 (0.01,2.18) | 0.14 (0.00,4.57) | 0.20 (0.02,2.18) | 0.24 (0.02,2.24) | 0.25 (0.01,5.83) | 0.28 (0.02,4.13) | 0.29 (0.07,1.25) | 0.43 (0.01,14.57) | 0.40 (0.03,6.27) | 0.44 (0.06,2.98) | 0.54 (0.05,5.23) | 0.79 (0.05,12.72) | I |

**Supplementary material 5. League plots of adverse event（A）, serious adverse event（B）, adverse event leading to discontinuation of study（C）, infection（D）, serious infection（E）, malignancy（F）.** Bold texts indicated that the comparison between the two groups was statistically significant. SUCRA:surface under the cumulative ranking curve; A:Placebo; B:tofacitinib 5mg; C:baricitinib 4mg; D:upadacitinib 15mg; E:upadacitinib 30mg; F:filgotinib 200mg; G:peficitinib 100mg; H:peficitinib 150mg; I:tofacitinib 5mg+csDMARD; J:baricitinib 2mg+csDMARD; K:baricitinib 4mg+csDMARD; L:upadacitinib 15mg+csDMARD; M:upadacitinib 30mg+csDMARD; N:filgotinib 200mg+csDMARD; O:peficitinib 100mg+csDMARD; P:peficitinib 150mg+csDMARD; Q:Conventional synthetic disease-modifying antirheumatic drugs(csDMARD)
